# Substrate dependent reaction channels of the Wolff–Kishner reduction reaction: A theoretical study

**DOI:** 10.3762/bjoc.10.21

**Published:** 2014-01-23

**Authors:** Shinichi Yamabe, Guixiang Zeng, Wei Guan, Shigeyoshi Sakaki

**Affiliations:** 1 Fukui Institute for Fundamental Chemistry, Kyoto University, Takano-Nishihiraki-cho 34-4, Sakyo-ku, Kyoto 606-8103, JAPAN. Phone: +81-075-711-7907

**Keywords:** acetone, acetophenone, DFT calculations, diimine intermediate, reduction reaction, transition states, Wolff–Kishner

## Abstract

Wolff–Kishner reduction reactions were investigated by DFT calculations for the first time. B3LYP/6-311+G(d,p) SCRF=(PCM, solvent = 1,2-ethanediol) optimizations were carried out. To investigate the role of the base catalyst, the base-free reaction was examined by the use of acetone, hydrazine (H_2_N–NH_2_) and (H_2_O)_8_. A ready reaction channel of acetone → acetone hydrazine (Me_2_C=N–NH_2_) was obtained. The channel involves two likely proton-transfer routes. However, it was found that the base-free reaction was unlikely at the N_2_ extrusion step from the isopropyl diimine intermediate (Me_2_C(H)–N=N–H). Two base-catalyzed reactions were investigated by models of the ketone, H_2_N–NH_2_ and OH^−^(H_2_O)_7_. Here, ketones are acetone and acetophenone. While routes of the ketone → hydrazone → diimine are similar, those from the diimines are different. From the isopropyl diimine, the N_2_ extrusion and the C–H bond formation takes place concomitantly. The concomitance leads to the propane product concertedly. From the (1-phenyl)ethyl substituted diimine, a carbanion intermediate is formed. The para carbon of the phenyl ring of the anion is subject to the protonation, which leads to a 3-ethylidene-1,4-cyclohexadiene intermediate. Its [1,5]-hydrogen migration gives the ethylbenzene product. For both ketone substrates, the diimines undergoing E2 reactions were found to be key intermediates.

## Introduction

**Scheme 1:**
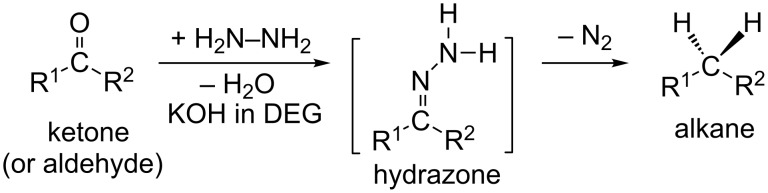
The Wolff–Kishner (W-K) reduction. DEG, diethylene glycol (HO–C_2_H_4_–O–C_2_H_4_–OH), is usually used as a solvent.

The Wolff–Kishner (W-K) reduction is an organic reaction to convert an aldehyde or ketone to an alkane by the use of hydrazine (H_2_N–NH_2_) and a base (OH^−^ or alkoxide RO^−^) [1–2]. The reaction is illustrated in Scheme 1.

It is described as a "homogeneous" reaction [3] because the platinum catalyst used initially is not included. The formal change of the W-K reduction in the former step is the conversion of the C=O bond to the C=N–NH_2_ group. The latter step involves a shift of two hydrogen atoms from the terminal nitrogen atom to the carbon and the simultaneous extrusion of an N_2_ molecule.

**Scheme 2:**
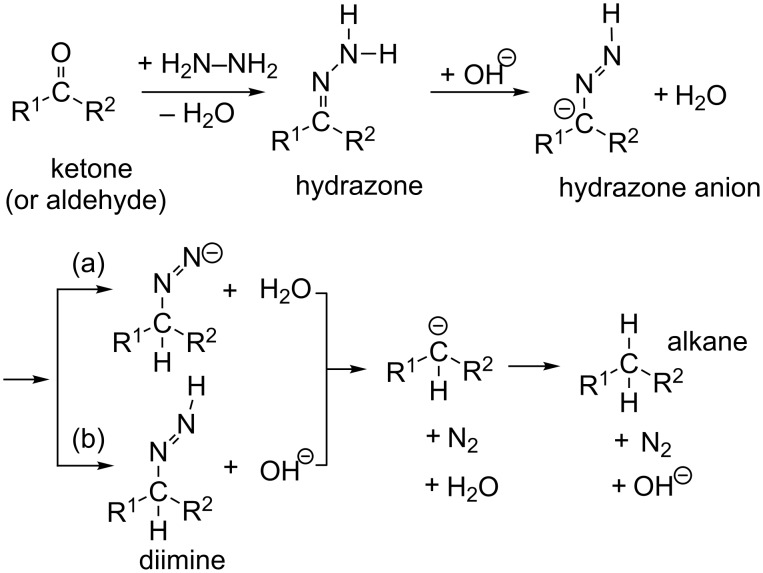
Mechanism of the Wolff–Kishner reduction. The route (a) is taken from ref. [6] and (b) from refs. [5,7–8].

The Huang-Minglon modification is an alternative and convenient method of the W-K reduction [4–5]. The method involves heating R^1^(R^2^)C=O, KOH and hydrazine hydrate (H_2_N–NH_2_·H_2_O) together in diethylene glycol (DEG in Scheme 1). This method gives the products from steroid ketones in a one-pot reaction and with a high yield. Scheme 2 shows the reaction mechanism.

From hydrazone, the hydrazone anion is formed, which was first suggested by Seibert [6–7]. From the anion, there are two routes (a) and (b). In (a), the proton is [1,3]-shifted to give an isomer anion, R^1^(R^2^)HC–N=N(−). In (b), a diimine intermediate R^1^(R^2^)HC–N=N–H is generated [6–8]. Both routes lead to a carbanion R^1^(R^2^)HC^−^. Protonation of the carbanion affords the product alkane. It is believed that the hydrazone anion is involved in the rate-determining step of the W-K reaction [9].

W-K reactions of hydrazones in aprotic solvents were investigated. Cram et al. obtained diphenylmethane H_2_C(Ph)_2_ in 85% yield from benzophenone hydrazone Ph_2_C=N–NH_2_ and potassium *tert*-butoxide KO*t*-Bu in DMSO [10]. Grundon et al. obtained the product also in 85% yield in toluene [11]. These non-aqueous experimental results indicate that the *tert*-butoxide ion directly participates in the proton shifts.

**Scheme 3:**
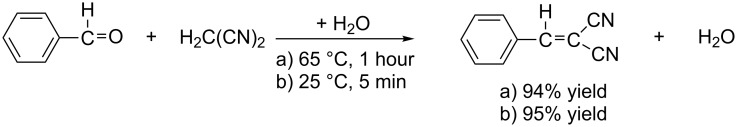
An uncatalyzed (without base) Knoevenagel condensation in water. Experimental conditions and yields are taken from a) ref. [13] and b) ref. [14], respectively.

As a (C=O → C=C, alkene) conversion reaction, the Knoevenagel condensation [12] was utilized. While the condensation is traditionally base-catalyzed, it was found to proceed readily in water even without any catalyst [13–14]. Not only can this synthesis be considered as clean (green chemistry), but also is this base-free reaction remarkably efficient and of high yield even under mild and simple conditions (Scheme 3). In fact, the neutral condensation was confirmed as probable by DFT calculations of a reaction system composed of Ph–CHO, H_2_C(CN)_2_ and (H_2_O)_12_ [15].

In spite of its fundamental character (e.g., from acetone to propane), the W-K reduction has not been studied theoretically yet. Several issues of the mechanism remain and have not been addressed yet.

1. The role of the base in the step of the hydrazone formation in Scheme 1 is unclear. Is the base required or not for the C=O → C=N–NH_2_ conversion? An investigation about the role of the proton transfers along the hydrogen bonds may be of interest.
2. Is a base-free W-K reduction comparable to the Knoevenagel condensation possible?
3. Which path (a) or (b) in Scheme 2 is more favorable?
4. Is the carbanion R^1^(R^2^)HC^−^ a probable intermediate?
5. Is the mechanism variant or invariant with respect to the substrate, ketone or aldehyde?

We addressed these questions by DFT calculations, which were carried out for two substrates, acetone and acetophenone. Reaction paths including proton transfers [16–36] were carefully examined. It is shown that acetophenone undergoes a remarkable elementary process, an [1,5]hydrogen shift.

## Results and Discussion

The sum of electronic energy (E_T_) and zero-point vibrational energy (ZPE) was used for the discussion in this section.

**Scheme 4:**
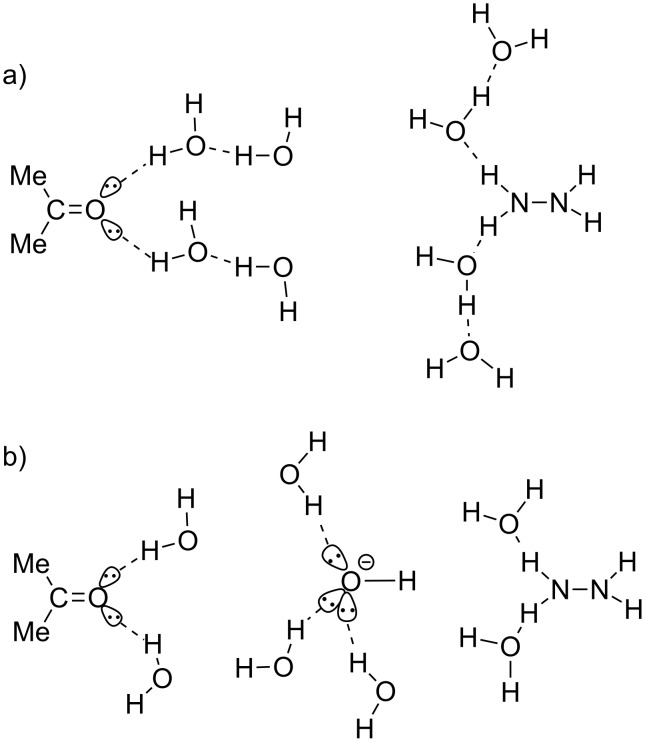
Reaction models of neutral (a) and anionic (b) systems. Water molecules are linked to oxygen lone-pair orbitals and to H–N bonds of hydrazine. In the Huang-Minglon method, hydrazine hydrate (H_2_N–NH_2_·H_2_O) was used [4–5]. Consequently, seven water molecules among eight do model the diol solvent molecules. It is necessary to apply this approximation, because it is too difficult to explicitly include these (e.g., DEG with the molecular formula C_4_H_10_O_3_).

**A neutral reaction of acetone.** In relation to issue 2, a base-free W-K reaction was examined. A model used for geometry optimizations is shown in Scheme 4a.

**Figure 1:**
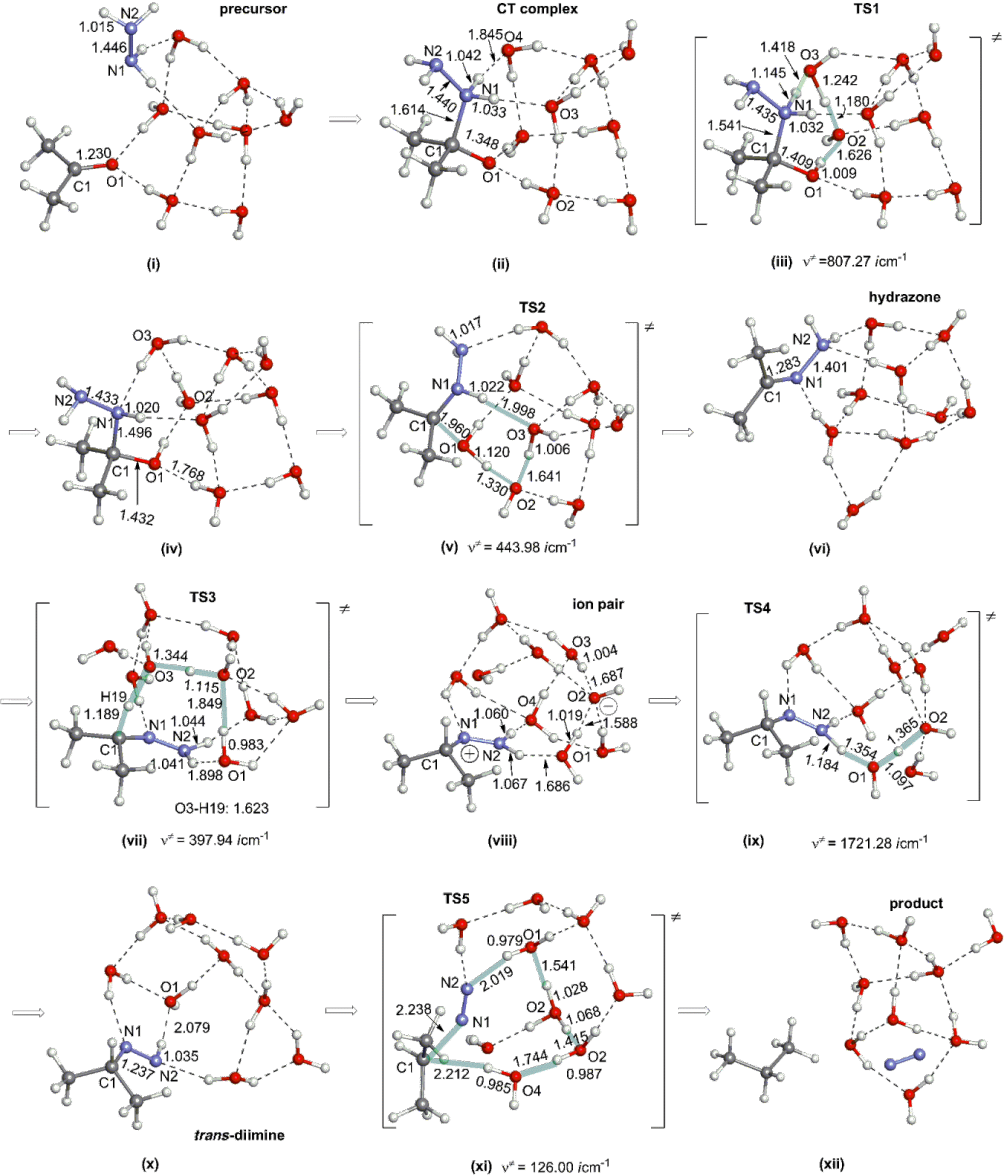
Geometric changes of the neutral Wolff–Kishner reduction reaction. The employed model is shown in Scheme 4a and is composed of acetone, hydrazine and (H_2_O)_8_. From (x) (trans-diimine and (H_2_O)_9_, there are two routes to the (xii) (product). One is (x) → (xi) → (xii) in this Figure. The other one is shown in Figure S1 (Supporting Information File 1). Distances are in Å.

The model of Scheme 4a is composed of acetone, hydrazine and (H_2_O)_8_ with the SCRF = PCM external field of ethylene glycol. Water dimers are linked to two lone-pair orbitals of the carbonyl oxygen and two N–H bonds. The model is based on Huang-Minglon conditions using H_2_N–NH_2_·H_2_O and diethylene glycol (DEG, bi-protic solvent). The DEG solvent was approximated by ethylene glycol. Figure 1 shows geometric changes of the neutral W-K reaction.

**Scheme 5:**
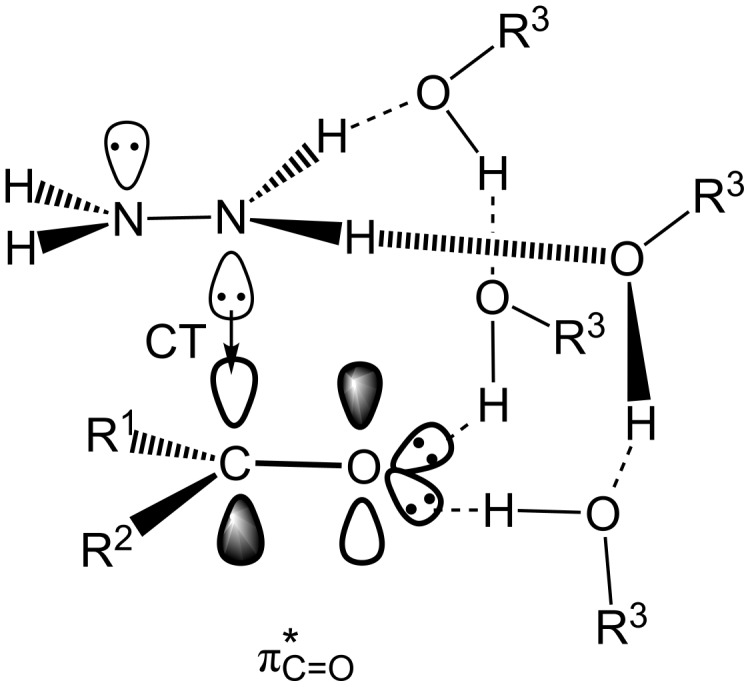
A CT complex between R^1^R^2^C=O and H_2_N–NH_2_ assisted by two hydrogen networks. R^3^–OH is an alcohol molecule.

In (i) of Figure 1, acetone and hydrazine molecules are distant. When they are close to each other, the geometry of the Me_2_(O=C)C....NH_2_–NH_2_ form was calculated in (ii). It is regarded as a Mulliken charge-transfer (CT) complex, because the C···N distance, 1.614 Å, is larger than the standard C–N bond length of 1.47 Å. The CT complex may be supported by two hydrogen-bond networks. The CT complex is probable even by protic solvents as shown in Scheme 5.

The CT complex assisted by hydrogen bonds was also obtained in the benzoic acid–ethylamine system by our recent study [37]. In (iii) of Figure 1, a proton transfer **TS1** was obtained along the first hydrogen bond. After this, an intermediate of (Me)_2_C(OH)–NH–NH_2_ is formed (iv). From the intermediate, the second proton transfer takes place along the second hydrogen bond (**TS2**, in (v)). After the transfer, acetone hydrazone, Me_2_C=N–NH_2_ [with (H_2_O)_9_], is afforded in (vi).

To the hydrazone carbon, a proton is transferred by **TS3** in (vii) of Figure 1. After **TS3**, an ion pair Me_2_CH–N=NH_2_(+) and OH(−) is generated in (viii).One proton is removed from the Me_2_CH–N=NH_2_(+) cation by **TS4** in (ix), which leads to a *trans*-diimine, Me_2_C(H)–N=NH [with (H_2_O)_9_], in (x). From the diimine, many TS searches of diimine + (H_2_O)_9_ → C_3_H_7_^–^ + N_2_ + H_3_O^+^(H_2_O)_8_ were attempted but failed. Instead, **TS5** with concomitant proton transfers involving four water molecules was obtained in (xi). **TS5** leads to the propane product without intervention of the isopropyl anion C_3_H_7_^−^. The product is shown in (xii) of Figure 1.

If the concerted process (without C_3_H_7_^−^) is likely, the *cis*-diimine would undergo proton transfers more readily than the *trans*-diimine. Then, a route of *trans*-diimine → *cis*-diimine → product was investigated. The transition state of the trans–cis isomerization, **TS6,** was obtained and is shown in Figure S1(xiii) in Supporting Information File 1. The resultant *cis*-diimine is shown in Figure S1(xiv). From the *cis*-diimine, two TSs, **TS7a** in Figure S1(xv) and **TS7b** in Figure S1(xvi), were determined. They involve (H_2_O)_2_ and (H_2_O)_3_ for proton transfers, respectively. After both TSs, the propane product (xii) was yielded. The superiority or inferiority of **TS5**, **TS7a** and **TS7b** will be examined in terms of their activation energies. So far, the reaction channel of the base-free W-K reduction was calculated.

**Figure 2:**
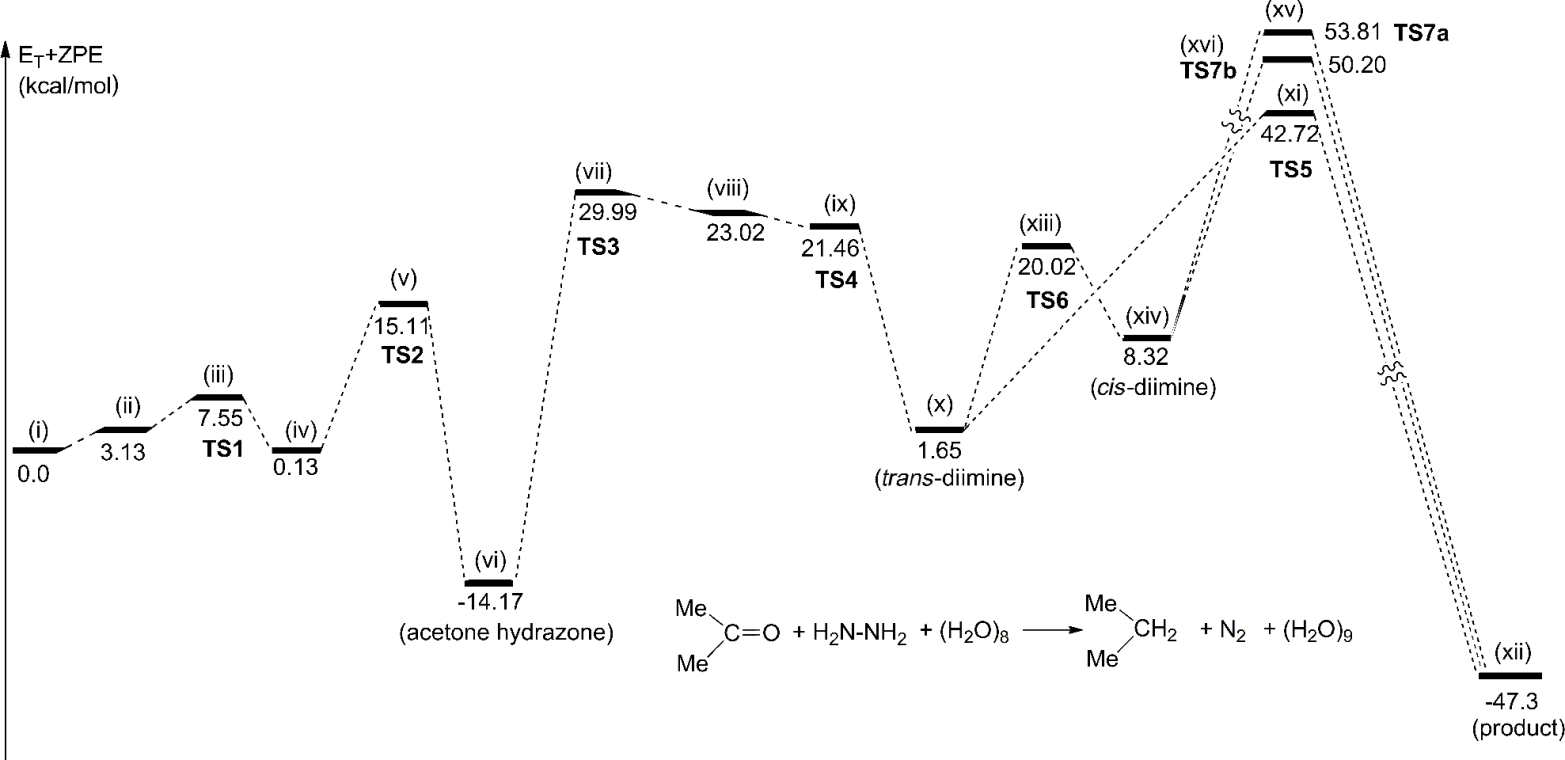
Energy changes of the neutral W-K reaction of acetone. Geometric changes are shown in Figure 1 and Figure S1 (Supporting Information File 1).

Figure 2 shows the energy changes along paths obtained in Figure 1 and Figure S1. From the starting-point precursor (i) to the acetone hydrazone (vi), **TS2** has the largest energy (+15.11 kcal/mol). However, this value means rather a ready process. Therefore, in the former process of the W-K reduction (ketone to hydrazone), the base catalyst is not required. In contrast, the latter process includes high-energy transition states, **TS5** with +42.72 kcal/mol, **TS7a** with +53.81 kcal/mol and **TS7b** with +50.20 kcal/mol. The conversion of the acetone hydrazone to propane without the base catalyst is clearly improbable.

**Figure 3:**
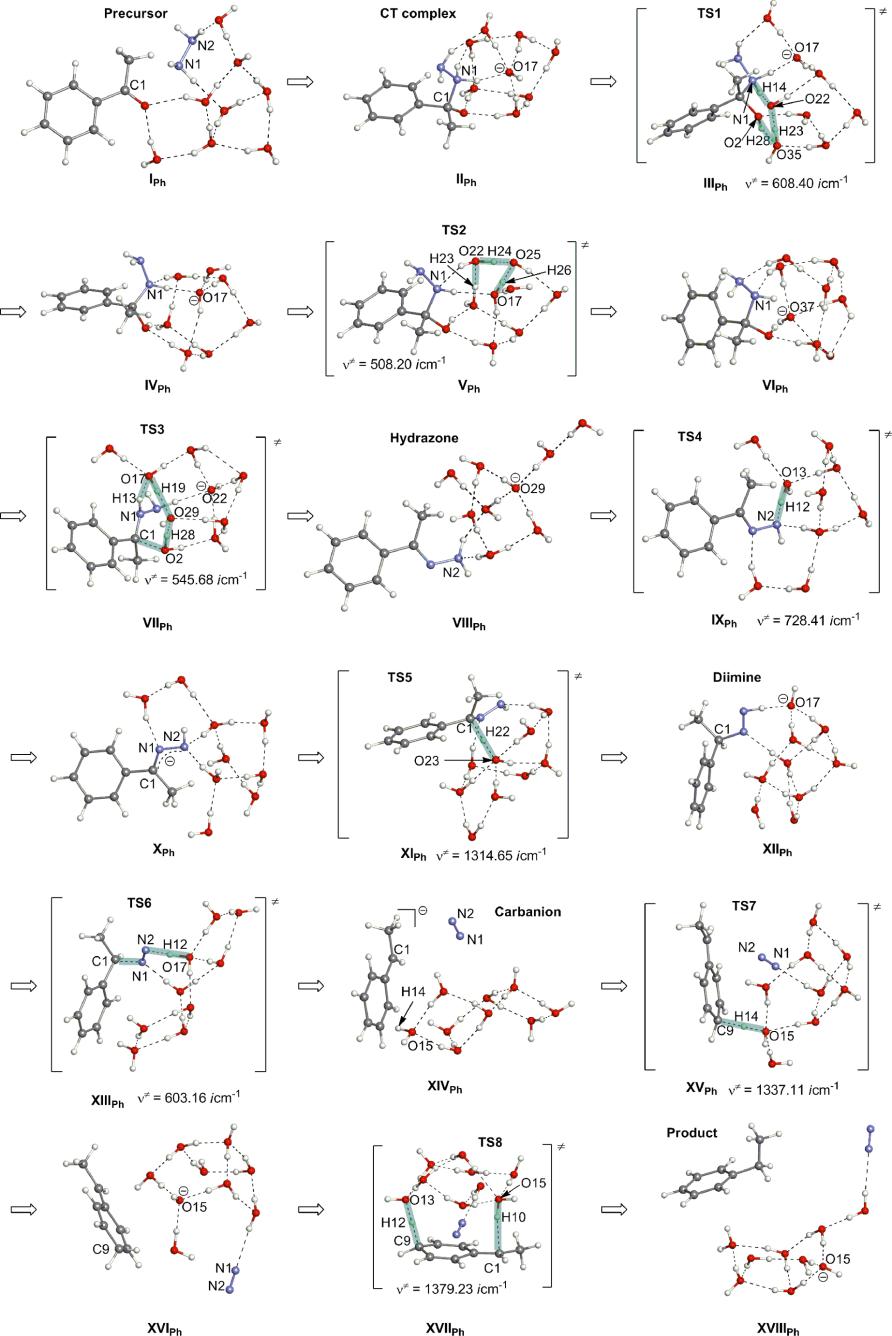
Geometric changes of the base-promoted Wolff–Kishner reduction reaction. The model employed is shown in Scheme 4b and is composed of acetone, hydrazine and OH^−^(H_2_O)_7_.

**A base-promoted reaction of acetone.** The reaction model adopted here is shown in Scheme 4b and consists of acetone, hydrazine and OH^−^(H_2_O)_7_. The model is iso-electronic with the neutral one in Scheme 4a. Figure 3 exhibits geometric changes of the OH^−^-containing W-K reaction. The process of precursor (**I_Me_**) → CT complex (**II_Me_**) → **TS1** (**III_Me_**) → Me_2_C(OH)–NH–NH_2_ (**IV_Me_**) is similar to that in Figure 1. However, at **IV_Me_** in Figure 3, the [O(17)–H(19)]^–^ ion blocks the second proton transfer. Its position is switched via **TS2** (**V_Me_**) to O(35)H(36)^–^, and a hydrogen bond circuit at the H_2_N–NH–C(OH)Me_2_(H_2_O)_2_ moiety is acquired (**VI_Me_**). Along the circuit, the proton transfer is brought about (**TS3**, **VII_Me_**). Then, the acetone hydrazone Me_2_C=N–NH_2_ is formed (**VIII_Me_**) in Figure 3. Routes of the conversion of the hydrazone to the *trans*-diimine were obtained in the sequence, hydrazone (**VIII_Me_**) → **TS4** (**IX_Me_**) → hydrazone anion (**X_Me_**) → **TS5** (**XI_Me_**) → *trans*-diimine (**XII_Me_**) in Figure 3.

**Figure 4:**
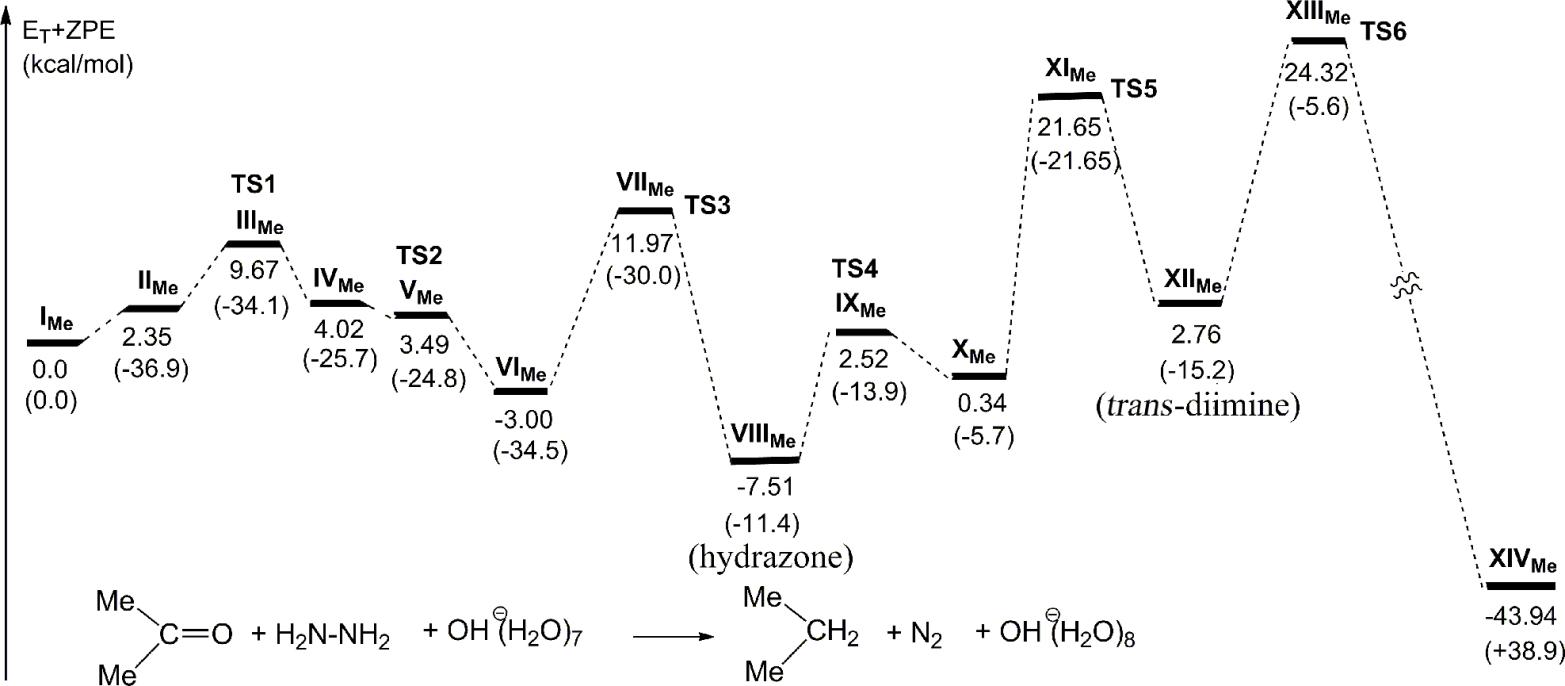
Energy changes of the OH^−^ containing W-K reaction of acetone calculated by B3LYP/6-311+G**. Geometric changes are shown in Figure 3. Values in parentheses are entropy changes Δ*S*^0^ in cal/(mol∙K).

From the diimine, an elimination TS (**TS6** in **XIII_Me_**) was obtained in Figure 3. In **TS6**, the N(2)–H(12) and N(1)–C(1) bonds are cleaved in a trans direction, where the dihedral angle H(12)–N(2)–N(1)–C(1) is 178°. The direction follows the pattern of the bimolecular nucleophilic elimination (E2). At **TS6**, the component of the C(1)···H(26) bond formation is also included, which means that the C_3_H_7_^−^ intermediate does not intervene at the N_2_ extrusion step. The proton H(26) shifts toward C(1) by the aid of the H(26)···N(1) attraction. After **TS6**, the propane product is formed, which is shown in **XIV_Me_**. The number of water molecules participating in bond interchanges is 2 in **TS1**, 3 in **TS2**, 3 in **TS3**, 1 in **TS4** or 4 in **TS5**. Then, eight water molecules are classified as reactants and catalysts. The catalytic water molecules may be replaced by protic solvents. In addition, while one O–H bond of the water molecules acting as a reactant is concerned with the interchange, almost all of the other water molecules may be O–R^3^ groups (see Scheme 5). Thus, the result in Figure 3 is applicable to the diol-solvent reaction and is considered to meet the the Huang-Minglon conditions. Figure 4 shows the energy change of the OHˉ containing reaction.

**Scheme 6:**
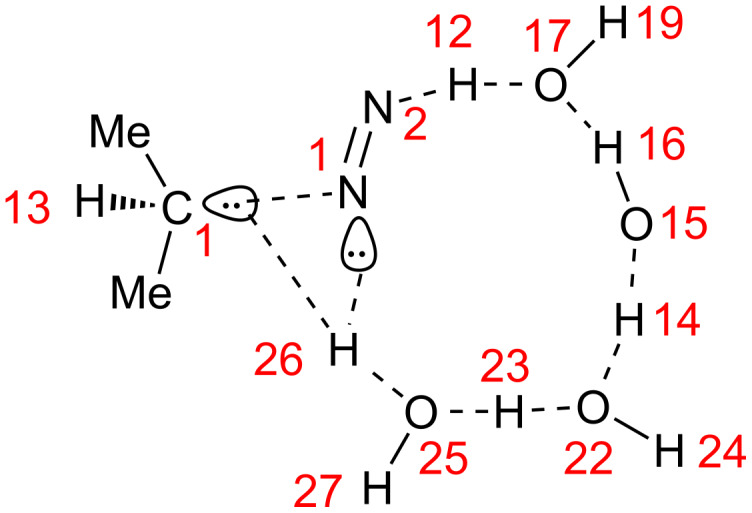
The main part of **TS6**. The N1···H26 hydrogen bond is converted into the C1–H26 covalent bond.

**TS5** and **TS6** have large activation energies, +21.65 and +24.32 kcal/mol, respectively, and the latter is the rate-determining step. The shape of **TS6** may be illustrated as in Scheme 6.

The energies in Figure 4 are much smaller than the energy of **TS5** of the neutral reaction shown in Figure 2 (+42.72 kcal/mol). The need of the base catalyst OH^−^ to promote the W-K reduction, particularly the nucleophilic elimination of the diimine, was revealed.

In parentheses, entropy changes are shown. In the first step, **I_Me_** → **II_Me_**, the large entropy decrease (−36.9 e.u.) is noticeable. The decrease originates from the formation of the CT complex with an ion-pair character, which results in the surrounding water cluster to adopt a more compact form. In the last step, **XIII_Me_** → **XIV_Me_**, the large entropy increase (+38.9 e.u.) is shown, which corresponds to the separated form of C_3_H_8_, N_2_ and OH^−^(H_2_O)_8_.

**Figure 5:**
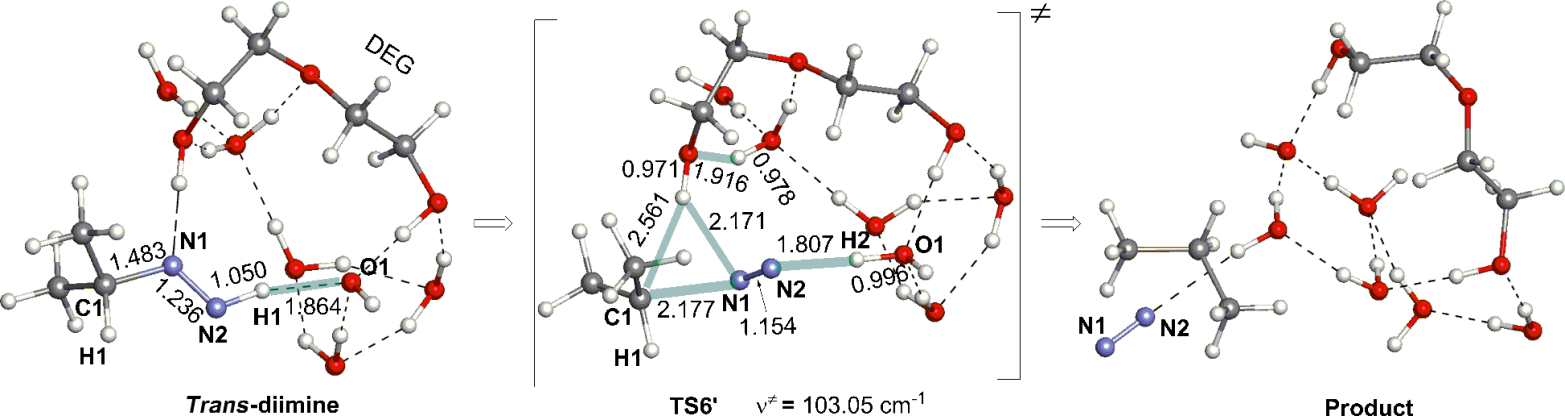
A *trans*-diimine → propane conversion step corresponding to **TS6** in Figure 3. The system is composed of *trans*-diimine (isopropyl–N=N–H), DEG (HO–C_2_H_4_–O–C_2_H_4_–OH) and OH^−^(H_2_O)_5_.

Diethylene glycol (DEG) is a solvent which has been most frequently used in the W-K reduction as shown in Scheme 1. The DEG molecule might explicitly participate in the bond interchange of **TS6** depicted in Scheme 6. Figure 5 shows the geometric change of the (diimine → product propane) elementary step.

A bond interchange similar to that shown in Scheme 6 was calculated in Figure 5. The energy barrier of the step is +24.06 kcal/mol, which is also similar to +24.32 kcal/mol in Figure 4. The similarity suggests that the water cluster in Scheme 4 may be a model of diol solvents such as DEG, ethylene glycol and triethylene glycol.

**Figure 6:**
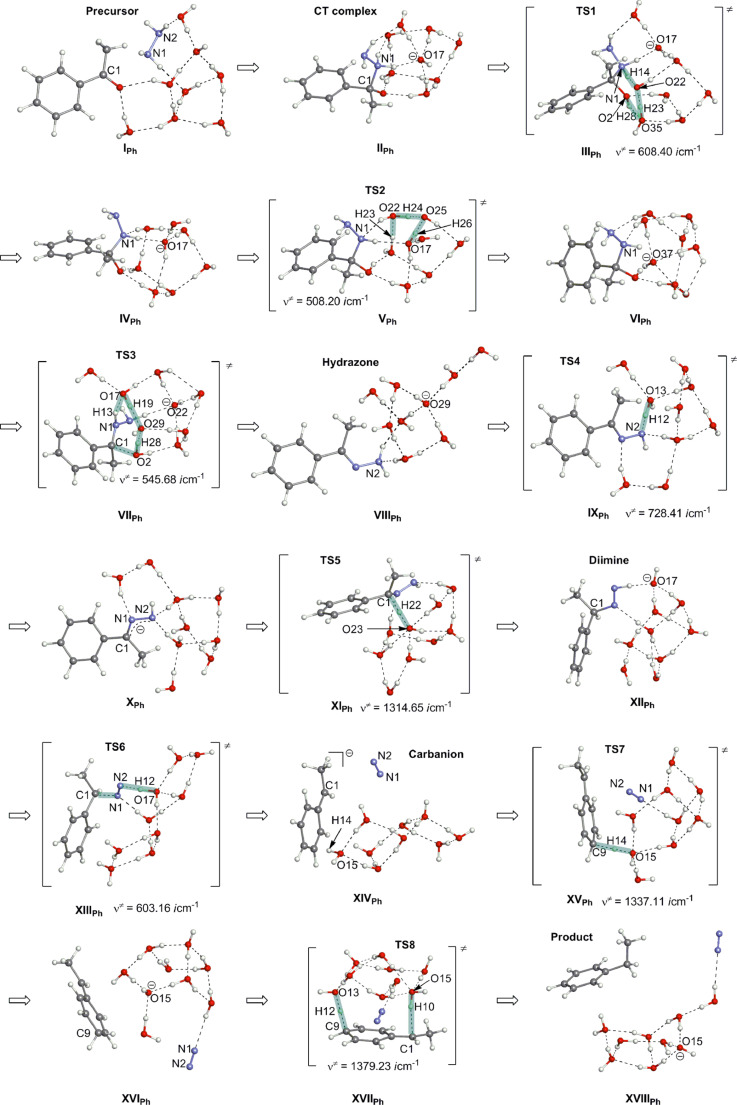
Geometric changes of the base-promoted Wolff–Kishner reduction reaction of acetophenone [Me–C(=O)–Ph], H_2_N–NH_2_ and OH^−^(H_2_O)_7_.

**A base-promoted reaction of acetophenone.** Figure 6 exhibits geometric changes.

Routes from the precursor (**I_Ph_**) to the *trans*-diimine (**XII_Ph_**) in Figure 6 are similar to those from the precursor (**I_Me_**) to the diimine (**XII_Me_**). However, **TS6** in **XIII_Ph_** is quite different from that in **XIII_Me_**, and the former is a typical E2 reaction. The 1-phenylethyl anion is the leaving group at the trans nucleophilic elimination. Thus, the carbanion is formed owing to the delocalization of the negative charge into the phenyl ring. The intermediate is shown in **XIV_Ph_** (Figure 6). It is noteworthy that the H(14)–O(15) bond of one water molecule is coordinated to the para position of the ring. In contrast, the carbanion center C(1) is not surrounded by water molecules just after the C–N_2_ cleavage. The region around C(1) would be transiently hydrophobic. By the frequency factor of the Arrhenius equation, the subsequent protonation of the para position rather than C(1) is conceivable. Indeed, **TS7** is obtained and is shown in **XV_Ph_**. After **TS7**, a 3-ethylidene-1,4-cyclohexadiene intermediate is afforded (**XVI_Ph_**). To recover the phenyl ring from it, a [1,5]-hydrogen rearrangement (**TS8**) is provoked by **XVII_Ph_**. After **TS8**, the ethylbenzene product was obtained and this is shown in **XVIII_Ph_** of Figure 6.

**Figure 7:**
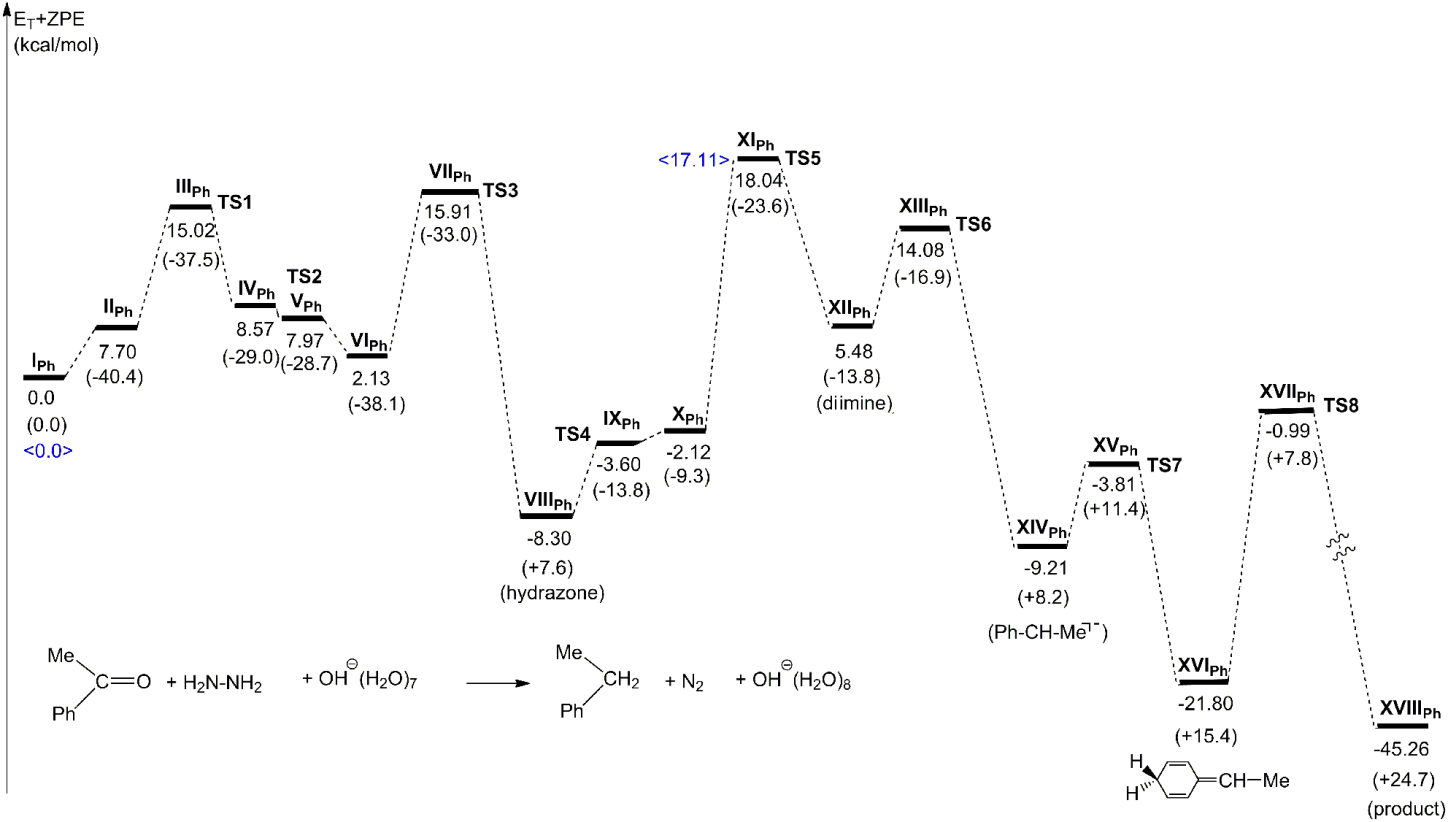
Energy changes of the OHˉ containing W-K reaction of acetophenone. Geometric changes are shown in Figure 6. <17.11 kcal/mol> of **XI_Ph_** is calculated by wB97D/6-311+G**.

Figure 7 shows energy changes of the acetophenone reaction. The rate-determining step was found to be **TS5** with the activation energy, +18.04 kcal/mol. The protonation of the anion Ph–C(−)Me–N–N–H in **X_Ph_** is difficult, because the anion character is delocalized in the phenyl ring by canonical resonance structures. The activation energy relative to the hydrazone is +26.34 (= + 18.04 − (−8.30)) kcal/mol, which is somewhat larger yet comparable to the experimental one, +22.9 kcal/mol in the solvent butyl carbitol, *n*-C_4_H_9_(OCH_2_CH_2_)_2_OH [9].

Reactions from the benzophenone hydrazone to the diphenylmethane in aprotic solvents such DMSO [10] and toluene [11] are discussed. Even if proton sources are absent, paths similar to **VIII_ph_** (hydrazone) → **IX_Ph_**(**TS4**) → **X_Ph_** → **XI_Ph_**(**TS5**) → **XII_Ph_** (diimine) → **XIII_Ph_**(**TS6**) →**XIV_Ph_** (carbanion) may be taken. However, the HO-*t*-Bu species formed by elimination (**TS6**) is far from the para positions of the phenyl rings. In addition, the anion character in the carbanion is delocalized, which leads to the low proton affinity of the para carbon atoms. Thus, in the aprotic solvent the carbanion center would be subject to the proton transfer directly from HO-*t*-Bu (without the **XVI_Ph_** type intermediate).

## Conclusion

**Scheme 7:**
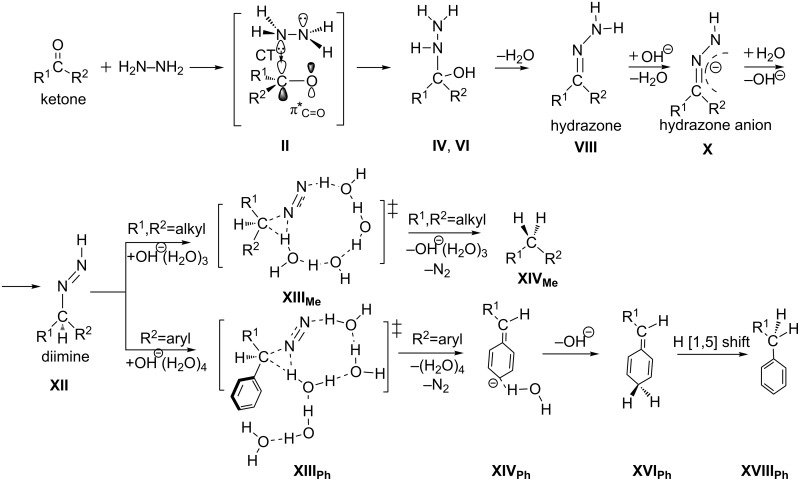
Elementary processes of the W-K reduction obtained by DFT calculations. From the diimine intermediate **XII**, two channels are depicted.

In this study, the W-K reduction was investigated by DFT calculations. First, the base-free reaction was examined by the use of acetone, hydrazine and (H_2_O)_8_. While the reaction channels were obtained, it was found to be unlikely because of the very large activation energy at the N_2_ extrusion step **TS5**, (xi). Second, elementary steps of OH^−^ containing two reactions were traced, where ketones are acetone and acetophenone. These results are summarized in Scheme 7.

From the ketone to the hydrazone, two hydrogen-bond circuits in Scheme 5 promote proton transfers for bond interchanges. At this stage, the OH^−^ ion works merely as a catalyst and does not participate in the reaction center. Paths of hydrazone → hydrazone anion → diimine were obtained. The Me_2_C(H)–N=N–H diimine in **XII_Me_** was found to undergo a concerted path to give the propane. The carbanion with alkyl groups does not intervene at the N2 extrusion step, and a proton assisted by an N···H hydrogen bond is migrated to the central carbon in **XIII_Me_**. In contrast, the PhMeC(H)–N=N–H diimine is converted to the carbanion at **XIV_Ph_**. To the para position of the phenyl ring of the anion, a proton is added, which leads to the cyclohexadiene intermediate. The subsequent [1,5]-hydrogen shift gives the product.

In the Introduction, five questions 1 to 5 have been raised. They may be addressed by means of the computed data.

1. At the ketone → hydrazone conversion step, the base catalyst is unnecessary as shown by the small activation energy (15.11 kcal/mol) of **TS2** of Figure 2 for the OH^−^-free reaction. In the Mulliken CT complex, two hydrogen bond circuits are formed. They promote proton transfers, leading to the conversion.
2. The base-free W-K reduction is unlikely owing to the large activation energy at the concerted N_2_ extrusion step.
3. The path (b) in Scheme 2 was obtained, which involves the diimine intermediate.
4. The presence or absence of the carbanion depends on the substituents. While the acetone substrate does not give the anion, the acetophenone yields the anion due to the anion delocalization in the phenyl ring.
5. In relation to 4, the conversion path from the diimine to the alkane varies with the ketone substrate. In contrast, the path form the ketone to the diimine is invariant.

## Computational details

The reacting systems were investigated by density functional theory calculations. The B3LYP [38–39] method was used. The basis set employed was 6-311+G(d,p). Geometry optimizations and vibrational analyses were carried out in the solution phase. Here, 1,2-ethanediol (ethylene glycol, HO–CH_2_–CH_2_–OH) was used as the solvent. The solvent effect was considered by the polarizable continuum model (PCM) [40–42]. Geometries of transition states (TSs) were determined by the input data consisting of two steps. For the first step, the initial geometry containing average and assumed distances between covalent and intermolecular interactions at the reaction center was prepared by the use of Z matrices. For instance, in the gas phase S_N_2 reaction Cl^−^ + H_3_C–Br → Cl–CH_3_ + Br^−^, Cl···C and C···Br intermediate distances were assumed. The geometry was optimized under the constraint of the fixed Cl–C and C–Br distances. After the partial optimization in the first step, a full optimization of the TS was carried out by the use of the Hessian force constant matrices in the second step. To the Cl···C and C···Br distances, negative force constants were specified to obtain the TS geometry of the bond interchange C–Br → Cl–C. TSs were characterized by vibrational analyses, which checked whether the obtained geometries have single imaginary frequencies (ν^≠^). From TSs, reaction paths were traced by the intrinsic reaction coordinate (IRC) method [43–44] to obtain the energy-minimum geometries. For the W-K reaction of acetophenone, wB97XD/6-311+G* calculations [45] were carried out on the precursor and the rate-determining step. All the calculations were carried out using the GAUSSIAN 09 program package [46]. The computations were performed at the Research Center for Computational Science, Okazaki, Japan.

## Supporting Information

File 1 Full citation of reference [46], Figure S1 for the neutral reaction, and Cartesian coordinates of optimized geometries in Figure 1, Figure 3, Figure 5 and Figure 6.
