# Supplementary material for: Substrate dependent reaction channels of the Wolff–Kishner reduction reaction: A theoretical study
Source: Beilstein J Org Chem. 2014 Jan 23;10:259–70. doi: 10.3762/bjoc.10.21 (PMC3943666; doi:10.3762/bjoc.10.21)
Supplement: File 1 — Full citation of reference [46], Figure S1 for the neutral reaction, and Cartesian coordinates of optimized geometries in Figure 1, Figure 3, Figure 5 and Figure 6. [file Beilstein_J_Org_Chem-10-259-s001.pdf]

Supporting Information

for

Substrate dependent reaction channels of the Wolff–Kishner reduction reaction: A theoretical study

Shinichi Yamabe,* Guixiang Zeng, Wei Guan and Shigeyoshi Sakaki

Address: Fukui Institute for Fundamental Chemistry, Kyoto University, Takano-Nishihiraki-cho 34-4, Sakyo-ku, Kyoto 606-8103, JAPAN. Phone: +81-075-711-7907.

Email: Shinichi Yamabe - yamabes@fukui.kyoto-u.ac.jp.

*corresponding author

Full citation of reference [46], Figure S1 for the neutral reaction, and Cartesian coordinates of optimized geometries in Figures 1, 3, 5, and 6.

Complete form of Reference 46

Gaussian 09, Revision B.01, Frisch, M. J.; Trucks, G. W.; Schlegel, H. B.; Scuseria, G. E.; Robb, M. A.; Cheeseman, J. R.; Scalmani, G.; Barone, V.; Mennucci, B.; Petersson, G. A.; Nakatsuji, H.; Caricato, M.; Li, X.; Hratchian, H. P.; Izmaylov, A. F.; Bloino, J.; Zheng, G.; Sonnenberg, J. L.; Hada,M.; Ehara, M.; Toyota, K.; Fukuda, R.; Hasegawa, J.; Ishida, M.; Nakajima, T.; Honda, Y.; Kitao, O.; Nakai, H.; Vreven, T.; Montgomery, Jr., J. A.; Peralta, J. E.; Ogliaro, F.; Bearpark, M.; Heyd, J. J.; Brothers, E.; Kudin, K. N.; Staroverov, V. N.; Kobayashi, R.; Normand, J.; Raghavachari, K.; Rendell, A.; Burant, J. C.; Iyengar, S. S.; Tomasi, J.; Cossi, M.; Rega, N.; Millam, N. J.; Klene, M.; Knox, J. E.; Cross, J. B.; Bakken, V.; Adamo, C.; Jaramillo, J.; Gomperts, R.; Stratmann, R. E.; Yazyev, O.; Austin, A. J.; Cammi, R.; Pomelli, C.; Ochterski, J. W.; Martin, R.L.; Morokuma, K.; Zakrzewski, V. G.; Voth, G. A.; Salvador, P.; Dannenberg, J. J.; Dapprich, S.; Daniels, A. D.; Farkas, O.; Foresman, J. B.; Ortiz, J. V.; Cioslowski, J.; Fox, D. J. Gaussian, Inc., Wallingford CT, 2010.

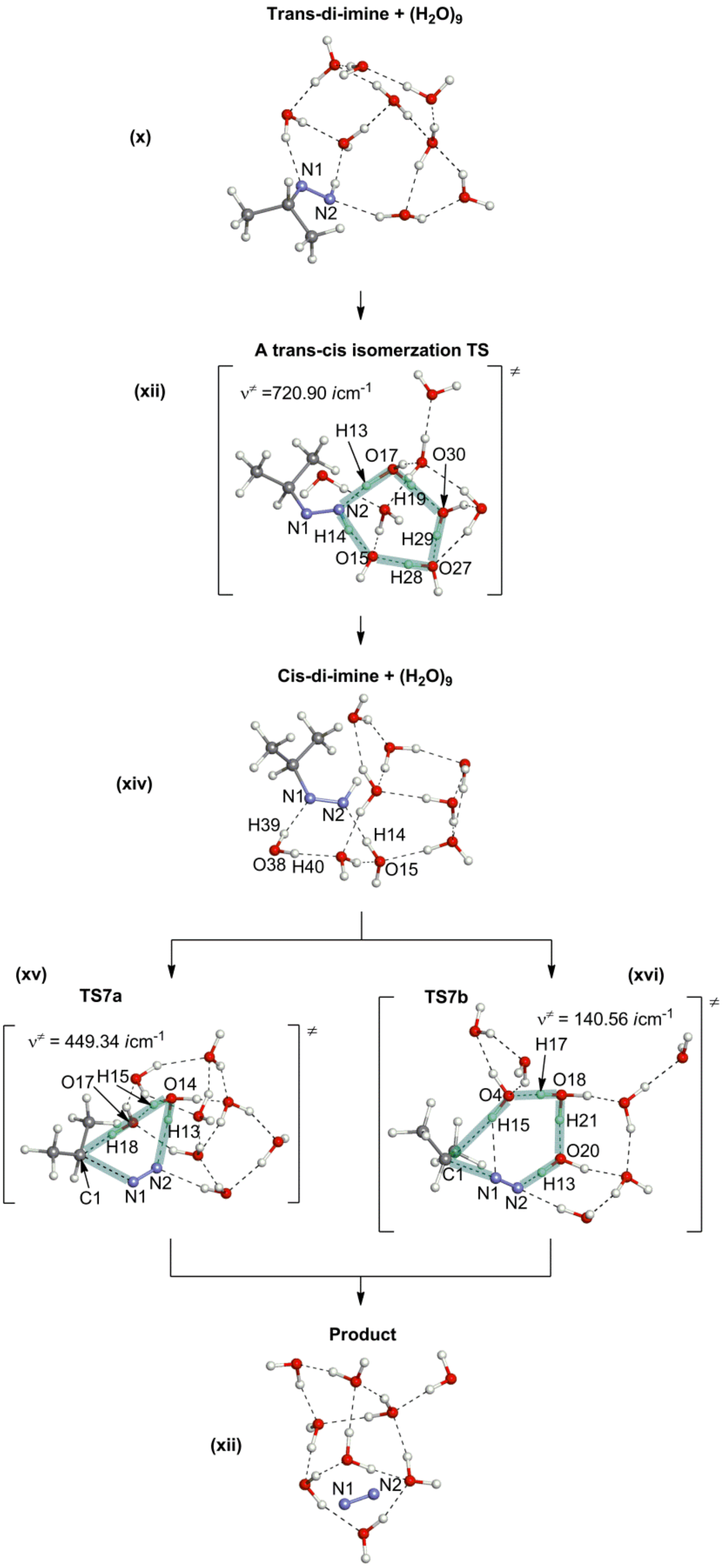

Figure S1: The second route from the trans-di-imine (x) to the product (xii).

Geometries of (x) and (xii) are also shown in Figure 1.

[1] **Geometries and energies in Figure 1**

(i) Precursor(weak complex between acetone and hydrazine)

wk00a.high.chk

Stoichiometry C3H26N2O9

Standard orientation:

Center Number	Atomic Number	Atomic Type	Coordinates (Angstroms) X	Y	Z
1	6	0	3.467917	-0.019992	-0.337174
2	8	0	2.355244	-0.484952	-0.094124
3	6	0	4.336207	0.539211	0.752712
4	6	0	4.015055	0.007391	-1.735753
5	1	0	3.960649	0.273539	1.739040
6	1	0	5.366939	0.197391	0.633625
7	1	0	4.350843	1.630322	0.655684
8	1	0	4.526114	0.952112	-1.932605
9	1	0	4.766219	-0.785443	-1.824076
10	1	0	3.232929	-0.160332	-2.474014
11	7	0	-0.406454	3.047721	-0.466227
12	7	0	-1.127513	4.278497	-0.700049
13	1	0	-0.486439	2.755906	0.508744
14	1	0	-0.850164	2.324440	-1.025912
15	1	0	-0.506164	5.046509	-0.469060

16	1	0	-1.932104	4.356555	-0.079986
17	8	0	-1.661792	0.344659	-1.707376
18	1	0	-2.523124	-0.009392	-1.987211
19	1	0	-0.998588	-0.353712	-1.865694
20	8	0	-4.371618	-0.556386	-1.373345
21	1	0	-4.111009	-0.377770	-0.449977
22	1	0	-5.174199	-0.045503	-1.524492
23	8	0	-0.941421	1.695923	2.421875
24	1	0	-0.247193	1.017022	2.478258
25	1	0	-1.683563	1.227578	1.996322
26	8	0	-2.733495	0.035131	0.884185
27	1	0	-2.203929	0.203876	0.077694
28	1	0	-2.361260	-0.799097	1.253787
29	1	0	1.154422	-1.204721	-1.276785
30	8	0	0.422378	-1.590036	-1.805014
31	1	0	0.799621	-1.814640	-2.664050
32	1	0	-0.329692	-3.018591	-0.930431
33	8	0	-0.802490	-3.668195	-0.371194
34	1	0	-0.228664	-4.440534	-0.321581
35	1	0	1.484671	-0.538203	1.549578
36	8	0	0.909152	-0.561010	2.341499
37	1	0	1.487124	-0.758831	3.087641
38	1	0	-0.571383	-1.721573	2.118386
39	8	0	-1.412862	-2.168490	1.909081
40	1	0	-1.204122	-2.773235	1.167647

Standard basis: 6-311+G(d,p) (6D, 7F)

478 basis functions

65 alpha electrons 65 beta electrons

nuclear repulsion energy 1092.9875639764 Hartrees.

NAtoms= 40 NActive= 40

Polarizable Continuum Model (PCM)

==================================

Model : PCM.

Solvent: 1,2-EthaneDiol, Eps= 40.245000 Eps(inf)= 2.050051

SCF Done: E(RB3LYP) = -916.960014188 A.U. after 1 cycles

Convg = 0.4906D-08 -V/T = 2.0047

Zero-point correction= 0.338830 (a.u.)

Thermal correction to Energy= 0.370956

Thermal correction to Enthalpy= 0.371900

Thermal correction to Gibbs Free Energy= 0.271395

Sum of electronic and zero-point Energies= -916.621184

Sum of electronic and thermal Energies= -916.589059

Sum of electronic and thermal Enthalpies= -916.588114

Sum of electronic and thermal Free Energies= -916.688619

	E (Thermal)	CV	S
	KCal/Mol	Cal/Mol-Kelvin	Cal/Mol-Kelvin
Total	232.778	104.148	211.531

(ii) Mulliken CT complex

Stoichiometry   C3H26N2O9

Standard orientation:

Center Number	Atomic Number	Atomic Type	Coordinates (Angstroms) X	Y	Z
1	6	0	2.243321	-0.363873	-0.459809
2	8	0	1.143582	-1.141851	-0.509478
3	6	0	3.339785	-0.918474	0.456009
4	6	0	2.789647	0.012123	-1.841831
5	1	0	2.944011	-1.125685	1.452704
6	1	0	3.694188	-1.858323	0.028471
7	1	0	4.199038	-0.248781	0.543887
8	1	0	2.016214	0.484096	-2.452347
9	1	0	3.657039	0.674647	-1.790109
10	1	0	3.103989	-0.906370	-2.340962
11	7	0	1.740223	1.010977	0.220028
12	7	0	2.612435	2.139023	0.421612
13	1	0	0.934603	1.338490	-0.336950
14	1	0	1.335508	0.765800	1.148826
15	1	0	3.386882	1.832187	1.003854
16	1	0	2.995013	2.404665	-0.481836
17	8	0	-0.784369	1.654211	-1.115945

18	1	0	-1.522090	2.281751	-1.217421
19	1	0	-0.935590	0.893255	-1.714685
20	8	0	-3.165142	3.033084	-0.431478
21	1	0	-3.083306	2.514961	0.389443
22	1	0	-3.298007	3.946775	-0.156361
23	8	0	0.384994	0.403640	2.688364
24	1	0	0.291095	-0.562155	2.567380
25	1	0	-0.494161	0.752618	2.441952
26	8	0	-2.072462	1.079387	1.370715
27	1	0	-1.545877	1.142914	0.550124
28	1	0	-2.416851	0.155356	1.365104
29	1	0	0.072054	-0.998189	-1.631316
30	8	0	-0.709667	-0.843823	-2.272010
31	1	0	-0.388293	-1.023995	-3.162412
32	1	0	-2.194979	-1.705980	-1.817388
33	8	0	-3.023180	-2.090525	-1.450156
34	1	0	-3.060361	-2.996379	-1.775559
35	1	0	0.539062	-1.842103	0.789301
36	8	0	0.062613	-2.167170	1.623221
37	1	0	0.443982	-3.018957	1.861082
38	1	0	-1.765113	-1.937055	1.436270
39	8	0	-2.678988	-1.619898	1.287567
40	1	0	-2.872223	-1.848646	0.354857

Standard basis: 6-311+G(d,p) (6D, 7F)

478 basis functions

65 alpha electrons 65 beta electrons

nuclear repulsion energy 1208.2413493062 Hartrees.

NAtoms= 40 NActive= 40

Polarizable Continuum Model (PCM)

=====================================

Model : PCM.

Atomic radii : UFF (Universal Force Field).

Polarization charges : Total charges.

Solvent: 1,2-EthaneDiol, Eps= 40.245000 Eps(inf)= 2.050051

SCF Done: E(RB3LYP) = -916.960102892 A.U. after 1 cycles

Convg = 0.1482D-08 -V/T = 2.0047

Zero-point correction= 0.343906 (a.u.)

Thermal correction to Energy= 0.371762

Thermal correction to Enthalpy= 0.372706

Thermal correction to Gibbs Free Energy= 0.287766

Sum of electronic and zero-point Energies= -916.616197

Sum of electronic and thermal Energies= -916.588341

Sum of electronic and thermal Enthalpies= -916.587397

Sum of electronic and thermal Free Energies= -916.672337

E (Thermal) CV S

KCal/Mol Cal/Mol-Kelvin Cal/Mol-Kelvin

Total	233.284	97.338	178.772

(iii) TS1

wk02b.high.log

Stoichiometry C3H26N2O9

Standard orientation:

Center Number	Atomic Number	Atomic Type	Coordinates (Angstroms) X	Y	Z
1	6	0	2.284305	-0.275392	-0.485700
2	8	0	1.187564	-1.139107	-0.675651
3	6	0	3.357472	-0.903771	0.398553
4	6	0	2.814772	0.116948	-1.858668
5	1	0	2.956188	-1.170796	1.377576
6	1	0	3.722928	-1.809143	-0.087822
7	1	0	4.205968	-0.230859	0.535242
8	1	0	2.038059	0.597239	-2.457411
9	1	0	3.672191	0.787575	-1.784224
10	1	0	3.143451	-0.784996	-2.376547
11	7	0	1.698908	0.959576	0.226646
12	7	0	2.540944	2.090862	0.489401
13	1	0	0.896175	1.298328	-0.325489
14	1	0	1.255104	0.643239	1.233009

15	1	0	3.222032	1.825249	1.194099
16	1	0	3.039864	2.350562	-0.356836
17	8	0	-0.829912	1.712409	-1.069052
18	1	0	-1.590227	2.323791	-1.042733
19	1	0	-1.057538	0.990737	-1.684532
20	8	0	-3.230954	2.986754	-0.294795
21	1	0	-3.148235	2.407391	0.488583
22	1	0	-3.342214	3.881370	0.044989
23	8	0	0.709619	0.183123	2.457957
24	1	0	0.416593	-0.973017	2.113164
25	1	0	-0.123620	0.649345	2.607330
26	8	0	-2.195626	1.071297	1.465282
27	1	0	-1.562286	1.121609	0.729503
28	1	0	-2.439669	0.117063	1.498121
29	1	0	-0.120269	-0.984289	-1.883640
30	8	0	-0.932239	-0.772774	-2.397400
31	1	0	-0.710537	-0.901509	-3.327097
32	1	0	-2.369176	-1.722984	-1.777868
33	8	0	-3.121901	-2.147088	-1.317012
34	1	0	-3.157736	-3.049990	-1.651020
35	1	0	0.869624	-1.553890	0.187684
36	8	0	0.170360	-1.999110	1.586011
37	1	0	0.561085	-2.731610	2.073952
38	1	0	-1.601577	-1.911474	1.490915
39	8	0	-2.555833	-1.675591	1.413034
40	1	0	-2.803060	-1.908014	0.496622

Standard basis: 6-311+G(d,p) (6D, 7F)

478 basis functions

65 alpha electrons 65 beta electrons

nuclear repulsion energy 1211.2934890941 Hartrees.

NAtoms= 40 NActive= 40

Polarizable Continuum Model (PCM)

=================================

Model : PCM.

Atomic radii : UFF (Universal Force Field).

Polarization charges : Total charges.

Solvent : 1,2-EthaneDiol, Eps= 40.245000 Eps(inf)= 2.050051

SCF Done: E(RB3LYP) = -916.947243097 A.U. after 1 cycles

Convg = 0.2026D-08 -V/T = 2.0047

Harmonic frequencies (cm**-1), IR intensities (KM/Mole)

activities (A**4/AMU), depolarization ratios for plane

incident light, reduced masses (AMU), force constants

and normal coordinates:

	1	2	3
	A	A	A
Frequencies --	-807.2679	35.6371	40.6865
Red. masses --	1.1439	5.1102	5.4189
Frc consts --	0.4392	0.0038	0.0053
IR Inten --	3847.8941	1.4317	4.2304

Zero-point correction= 0.338094 (a.u.)

Thermal correction to Energy= 0.365263

Thermal correction to Enthalpy= 0.366207

Thermal correction to Gibbs Free Energy= 0.282100

Sum of electronic and zero-point Energies= -916.609149

Sum of electronic and thermal Energies= -916.581980

Sum of electronic and thermal Enthalpies= -916.581036

Sum of electronic and thermal Free Energies= -916.665143

	E (Thermal)	CV	S
	KCal/Mol	Cal/Mol-Kelvin	Cal/Mol-Kelvin
Total	229.206	95.521	177.019

(iv) Me2C(OH)-NH-NH2 +(H2O)8

wk02b.for.high.log

Stoichiometry C3H26N2O9

Standard orientation:

Center Number	Atomic Number	Atomic Type	Coordinates (Angstroms) X	Y	Z
1	6	0	2.388595	-0.237036	-0.455175
2	8	0	1.303051	-1.168337	-0.528851
3	6	0	3.496799	-0.781256	0.444582
4	6	0	2.869289	-0.039599	-1.894055

5	1	0	3.125304	-0.943005	1.459060
6	1	0	3.856798	-1.731208	0.045954
7	1	0	4.342745	-0.092846	0.492229
8	1	0	2.056737	0.330910	-2.523508
9	1	0	3.697904	0.669754	-1.942613
10	1	0	3.223335	-0.988350	-2.301653
11	7	0	1.809441	1.006588	0.141803
12	7	0	2.671355	2.145863	0.248712
13	1	0	1.016484	1.290329	-0.433249
14	1	0	0.949768	0.891605	1.804814
15	1	0	3.274673	2.021399	1.054283
16	1	0	3.264449	2.252680	-0.572684
17	8	0	-1.072708	1.599516	-1.302488
18	1	0	-1.904994	2.086849	-1.430113
19	1	0	-1.130501	0.782784	-1.832428
20	8	0	-3.603029	2.724040	-0.567211
21	1	0	-3.349208	2.305807	0.276606
22	1	0	-3.802798	3.642705	-0.356830
23	8	0	0.400184	0.745186	2.615079
24	1	0	0.301154	-0.972617	2.416373
25	1	0	-0.494482	1.029076	2.343167
26	8	0	-2.113080	1.143485	1.285072
27	1	0	-1.623404	1.189417	0.438114
28	1	0	-2.392738	0.201893	1.357992
29	1	0	0.010332	-1.141387	-1.735028
30	8	0	-0.783151	-1.027149	-2.308576

31	1	0	-0.505688	-1.242609	-3.206651
32	1	0	-2.210049	-1.959541	-1.679099
33	8	0	-2.974800	-2.354680	-1.210588
34	1	0	-2.984818	-3.285562	-1.458488
35	1	0	1.012512	-1.437163	0.368912
36	8	0	0.208109	-1.861700	1.986137
37	1	0	0.608414	-2.509004	2.577899
38	1	0	-1.675533	-1.863672	1.652175
39	8	0	-2.589034	-1.590542	1.448201
40	1	0	-2.764399	-1.926620	0.544845

Standard basis: 6-311+G(d,p) (6D, 7F)

478 basis functions

65 alpha electrons 65 beta electrons

nuclear repulsion energy 1190.2161542088 Hartrees.

NAtoms= 40 NActive= 40

Polarizable Continuum Model (PCM)

=================================

Model : PCM.

Atomic radii : UFF (Universal Force Field).

Polarization charges : Total charges.

Charge compensation : None.

Solvent : 1,2-EthaneDiol, Eps= 40.245000 Eps(inf)= 2.050051

SCF Done: E(RB3LYP) = -916.965132491 A.U. after 1 cycles

Convg = 0.1554D-08 -V/T = 2.0046

Zero-point correction= 0.344163 (.a.u)

Thermal correction to Energy= 0.372488

Thermal correction to Enthalpy= 0.373432

Thermal correction to Gibbs Free Energy= 0.286579

Sum of electronic and zero-point Energies= -916.620969

Sum of electronic and thermal Energies= -916.592644

Sum of electronic and thermal Enthalpies= -916.591700

Sum of electronic and thermal Free Energies= -916.678553

	E (Thermal)	CV	S
	KCal/Mol	Cal/Mol-Kelvin	Cal/Mol-Kelvin
Total	233.740	98.641	182.798

(v) TS2

Stoichiometry C3H26N2O9

Standard orientation:

Center Number	Atomic Number	Atomic Type	Coordinates (Angstroms) X	Y	Z
1	6	0	-2.631454	-0.398358	0.425859
2	8	0	-1.527380	-1.017358	-1.069979
3	6	0	-3.867581	-0.080101	-0.367329

4	6	0	-2.639741	-1.690531	1.196533
5	1	0	-3.703150	0.681424	-1.127700
6	1	0	-4.233720	-0.981961	-0.852241
7	1	0	-4.635414	0.285009	0.322780
8	1	0	-1.700228	-1.854280	1.721318
9	1	0	-3.446335	-1.630653	1.933305
10	1	0	-2.841515	-2.529635	0.533555
11	7	0	-1.964359	0.603869	1.014090
12	7	0	-1.975009	1.939663	0.540179
13	1	0	-1.114131	0.362956	1.526470
14	1	0	-0.203963	2.871765	0.249517
15	1	0	-2.079092	1.948341	-0.471844
16	1	0	-2.775464	2.424293	0.938089
17	8	0	0.690157	-0.469110	1.735313
18	1	0	1.361989	-0.668218	2.405347
19	1	0	0.635011	-1.252327	1.106023
20	8	0	3.369222	-0.119457	2.899371
21	1	0	3.371272	0.498811	2.145890
22	1	0	3.693537	0.385416	3.652804
23	8	0	0.553717	3.102749	-0.320919
24	1	0	0.140862	2.025940	-1.675548
25	1	0	1.317115	2.623787	0.066067
26	8	0	2.490660	1.281815	0.562983
27	1	0	1.774288	0.689921	0.887367
28	1	0	2.703467	0.933711	-0.333228
29	1	0	-0.681135	-1.624325	-0.658099

30	8	0	0.346492	-2.287111	-0.134260
31	1	0	0.058848	-3.160292	0.153362
32	1	0	1.646573	-2.429256	-1.101400
33	8	0	2.472579	-2.409286	-1.676899
34	1	0	2.283213	-2.967519	-2.438220
35	1	0	-1.118270	-0.242919	-1.501106
36	8	0	-0.072496	1.267261	-2.272109
37	1	0	-0.372902	1.650618	-3.104390
38	1	0	1.760327	0.563263	-2.289017
39	8	0	2.666614	0.310224	-2.037765
40	1	0	2.650525	-0.672464	-1.985262

%chk=wk02c.high.chk

Standard basis: 6-311+G(d,p) (6D, 7F)

478 basis functions

65 alpha electrons 65 beta electrons

nuclear repulsion energy 1196.4006644199 Hartrees.

NAtoms= 40 NActive= 40

Polarizable Continuum Model (PCM)

==================================

Model : PCM.

Atomic radii : UFF (Universal Force Field).

Polarization charges : Total charges.

Solvent : 1,2-EthaneDiol, Eps= 40.245000 Eps(inf)= 2.050051

SCF Done: E(RB3LYP) = -916.935947503 A.U. after 1 cycles

Convg = 0.2206D-08 -V/T = 2.0046

	1	2	3
	A	A	A
Frequencies --	-443.9822	35.4112	40.6367

Zero-point correction= 0.338840 (a.u.)

Thermal correction to Energy= 0.366480

Thermal correction to Enthalpy= 0.367424

Thermal correction to Gibbs Free Energy= 0.282168

Sum of electronic and zero-point Energies= -916.597108

Sum of electronic and thermal Energies= -916.569468

Sum of electronic and thermal Enthalpies= -916.568523

Sum of electronic and thermal Free Energies= -916.653780

	E (Thermal)	CV	S
	KCal/Mol	Cal/Mol-Kelvin	Cal/Mol-Kelvin
Total	229.970	96.326	179.437

(vi) acetone hydrazone

Stoichiometry C3H26N2O9

Standard orientation:

Center Atomic Atomic Coordinates (Angstroms)

Number Number Type X Y Z

1	6	0	-3.548084	-0.034289	0.291888
2	8	0	0.894567	-1.947678	1.738051
3	6	0	-4.489193	-1.142685	-0.103842
4	6	0	-4.104103	1.353683	0.431442
5	1	0	-4.561283	-1.902345	0.683102
6	1	0	-5.491328	-0.757040	-0.286224
7	1	0	-4.139586	-1.645272	-1.011435
8	1	0	-3.334331	2.063762	0.732939
9	1	0	-4.542477	1.684195	-0.516123
10	1	0	-4.907992	1.366610	1.174545
11	7	0	-2.296913	-0.212444	0.510880
12	7	0	-1.751804	-1.489863	0.325245
13	1	0	-1.009005	1.047214	0.663646
14	1	0	-0.782787	-1.608334	-1.358140
15	1	0	-0.926881	-1.591382	0.922672
16	1	0	-2.407625	-2.243681	0.522301
17	8	0	-0.181869	1.597050	0.688777
18	1	0	-0.354109	2.433717	0.220810
19	1	0	1.024580	1.177315	1.864978
20	8	0	0.044336	3.560081	-1.353746
21	1	0	0.468029	2.818631	-1.824138
22	1	0	-0.617255	3.913630	-1.958248
23	8	0	-0.120039	-1.622571	-2.083788
24	1	0	1.287003	-2.370251	-1.346425
25	1	0	0.203515	-0.700548	-2.138061

26	8	0	1.082056	0.944915	-1.764595
27	1	0	0.758295	1.010309	-0.844192
28	1	0	2.016209	0.643050	-1.680620
29	1	0	1.256627	-1.092533	2.031421
30	8	0	1.719528	0.735905	2.408281
31	1	0	1.611547	1.060129	3.309716
32	1	0	3.382107	0.844617	1.691724
33	8	0	4.219438	0.870014	1.182285
34	1	0	4.880196	0.443587	1.738699
35	1	0	1.410431	-2.198059	0.949925
36	8	0	2.090745	-2.580502	-0.807672
37	1	0	2.362371	-3.473699	-1.046970
38	1	0	3.219437	-1.091532	-1.134443
39	8	0	3.564840	-0.197246	-1.317378
40	1	0	3.874799	0.144071	-0.452991

%chk=wk02c.rev.high.chk

Standard basis: 6-311+G(d,p) (6D, 7F)

478 basis functions

65 alpha electrons 65 beta electrons

nuclear repulsion energy 1133.2968333000 Hartrees.

NAtoms= 40 NActive= 40

Polarizable Continuum Model (PCM)

=================================

Model : PCM.

Atomic radii : UFF (Universal Force Field).

Polarization charges : Total charges.

Solvent: 1,2-EthaneDiol, Eps= 40.245000 Eps(inf)= 2.050051

SCF Done: E(RB3LYP) = -916.983908188 A.U. after 1 cycles

Convg = 0.7942D-08 -V/T = 2.0046

Zero-point correction= 0.340146 (a.u.)

Thermal correction to Energy= 0.369928

Thermal correction to Enthalpy= 0.370872

Thermal correction to Gibbs Free Energy= 0.279553

Sum of electronic and zero-point Energies= -916.643762

Sum of electronic and thermal Energies= -916.613981

Sum of electronic and thermal Enthalpies= -916.613036

Sum of electronic and thermal Free Energies= -916.704355

	E (Thermal)	CV	S
	KCal/Mol	Cal/Mol-Kelvin	Cal/Mol-Kelvin
Total	232.133	101.521	192.197

(vii) TS3

wk03d.high.log

Stoichiometry C3H26N2O9

Standard orientation:

Center Atomic Atomic Coordinates (Angstroms)

Number	Number	Type	X	Y	Z
1	6	0	-1.838062	-0.261716	-1.243066
2	6	0	-1.820336	-1.661035	-1.860579
3	6	0	-3.176688	0.463490	-1.367355
4	1	0	-1.869328	-1.598849	-2.952777
5	1	0	-0.945165	-2.247937	-1.582167
6	1	0	-2.700471	-2.203399	-1.513514
7	1	0	-3.134199	1.457312	-0.919541
8	1	0	-3.471280	0.565898	-2.415747
9	1	0	-3.950007	-0.109925	-0.853780
10	7	0	-0.762290	0.601921	-1.527105
11	7	0	0.361070	0.132865	-1.813561
12	1	0	0.560867	-0.886877	-1.866703
13	8	0	1.447508	-2.443129	-1.238390
14	1	0	1.182718	0.774424	-1.751566
15	1	0	1.389113	-2.241574	-0.277835
16	1	0	2.388670	-2.325585	-1.428184
17	8	0	1.407870	-1.353989	1.343755
18	1	0	1.553158	-1.929216	2.104102
19	1	0	-1.576153	-0.400794	-0.091313
20	8	0	-0.964966	-0.708014	1.380025
21	1	0	-1.017862	0.180267	1.765256
22	1	0	0.330298	-1.067864	1.371316
23	1	0	-0.945721	2.555429	-0.955130
24	8	0	-1.124654	3.344604	-0.412129

25	1	0	-0.511705	4.021613	-0.721264
26	8	0	2.644473	1.508818	-1.044645
27	1	0	3.240136	0.742275	-0.944912
28	1	0	2.381244	1.686097	-0.114097
29	8	0	3.782391	-0.965258	-0.202793
30	1	0	3.093810	-1.091488	0.480506
31	1	0	4.629081	-1.083236	0.241844
32	1	0	1.832285	0.497082	1.691547
33	8	0	1.948236	1.466682	1.666296
34	1	0	1.070924	1.822619	1.908828
35	8	0	-2.492686	-2.484040	2.703917
36	1	0	-3.347502	-2.492917	2.262033
37	1	0	-1.950440	-1.810442	2.210966
38	8	0	-0.705751	2.285008	2.108388
39	1	0	-0.922036	2.775243	1.285469
40	1	0	-1.030754	2.818442	2.841934

Standard basis: 6-311+G(d,p) (6D, 7F)

478 basis functions

65 alpha electrons   65 beta electrons

nuclear repulsion energy   1183.2780130350 Hartrees.

NAtoms=   40 NActive=   40

Polarizable Continuum Model (PCM)

=================================

Model   : PCM.

Atomic radii : UFF (Universal Force Field).

Solvent: 1,2-EthaneDiol, Eps= 40.245000 Eps(inf)= 2.050051

SCF Done: E(RB3LYP) = -916.905870515 A.U. after 1 cycles

Convg = 0.1494D-08 -V/T = 2.0047

	1	2	3
	A	A	A
Frequencies --	-397.9365	26.0689	27.8844

Zero-point correction= 0.332482 (a.u.)

Thermal correction to Energy= 0.361923

Thermal correction to Enthalpy= 0.362868

Thermal correction to Gibbs Free Energy= 0.272496

Sum of electronic and zero-point Energies= -916.573389

Sum of electronic and thermal Energies= -916.543947

Sum of electronic and thermal Enthalpies= -916.543003

Sum of electronic and thermal Free Energies= -916.633374

	E (Thermal)	CV	S
	KCal/Mol	Cal/Mol-Kelvin	Cal/Mol-Kelvin
Total	227.110	99.733	190.203

(viii) ion pair

Stoichiometry C3H26N2O9

Standard orientation:

Center Number	Atomic Number	Atomic Type	Coordinates (Angstroms) X	Y	Z
1	6	0	-2.919522	-0.108292	-0.384801
2	6	0	-3.148346	-1.601304	-0.160608
3	6	0	-4.002056	0.562885	-1.238242
4	1	0	-3.099260	-2.150924	-1.105360
5	1	0	-2.438850	-2.036171	0.543421
6	1	0	-4.148096	-1.735589	0.254763
7	1	0	-3.798148	1.627470	-1.362110
8	1	0	-4.062179	0.098547	-2.225442
9	1	0	-4.968611	0.449301	-0.745607
10	7	0	-1.625443	0.264706	-0.958840
11	7	0	-0.677814	-0.517563	-0.887607
12	1	0	-0.667744	-1.458572	-0.385757
13	8	0	-0.005992	-2.641461	0.617466
14	1	0	0.243568	-0.187006	-1.295019
15	1	0	0.900583	-2.240368	0.853280
16	1	0	0.162707	-3.510379	0.237217
17	8	0	2.224812	-1.398416	1.093546
18	1	0	2.764367	-1.830313	1.765684
19	1	0	-2.870300	0.396599	0.596452
20	8	0	0.649768	0.455706	2.398855
21	1	0	0.865111	1.295119	1.956308
22	1	0	1.207295	-0.235810	1.957930

23	1	0	-0.695248	2.296382	-1.526404
24	8	0	0.154913	2.755437	-1.597260
25	1	0	0.715909	2.138972	-2.093196
26	8	0	1.718176	0.267183	-1.990452
27	1	0	2.101288	-0.621416	-2.147848
28	1	0	2.313663	0.608728	-1.273510
29	8	0	2.814559	-2.220175	-1.420436
30	1	0	2.684514	-1.978997	-0.459767
31	1	0	3.755158	-2.397225	-1.526493
32	1	0	2.971444	0.062066	0.590471
33	8	0	3.288129	0.889755	0.133441
34	1	0	2.823613	1.631039	0.557274
35	8	0	-2.082634	0.490383	2.714869
36	1	0	-2.339757	-0.129562	3.405113
37	1	0	-1.106338	0.400156	2.631090
38	8	0	1.353675	2.839493	0.930187
39	1	0	0.893435	2.934059	0.067646
40	1	0	1.410964	3.720928	1.315171

%chk=wk03e.highfor1.chk

Standard basis: 6-311+G(d,p) (6D, 7F)

478 basis functions

65 alpha electrons 65 beta electrons

nuclear repulsion energy 1179.4472353378 Hartrees.

NAtoms= 40 NActive= 40

Polarizable Continuum Model (PCM)

=====================================

Model : PCM.

Atomic radii : UFF (Universal Force Field).

Solvent : 1,2-EthaneDiol, Eps= 40.245000 Eps(inf)= 2.050051

SCF Done: E(RB3LYP) = -916.921757471 A.U. after 1 cycles

Convg = 0.3501D-08 -V/T = 2.0047

Zero-point correction= 0.337265 (a.u.)

Thermal correction to Energy= 0.366773

Thermal correction to Enthalpy= 0.367718

Thermal correction to Gibbs Free Energy= 0.277247

Sum of electronic and zero-point Energies= -916.584492

Sum of electronic and thermal Energies= -916.554984

Sum of electronic and thermal Enthalpies= -916.554040

Sum of electronic and thermal Free Energies= -916.644511

	E (Thermal)	CV	S
	KCal/Mol	Cal/Mol-Kelvin	Cal/Mol-Kelvin
Total	230.154	100.011	190.412

(ix) TS4

Stoichiometry C3H26N2O9

Standard orientation:

Center Number	Atomic Number	Atomic Type	Coordinates (Angstroms) X	Y	Z
1	6	0	-3.094947	-1.086315	0.134709
2	6	0	-2.868934	-1.805229	-1.187167
3	6	0	-4.532815	-0.565341	0.312965
4	1	0	-3.074557	-1.151282	-2.038689
5	1	0	-1.853150	-2.190214	-1.279205
6	1	0	-3.556336	-2.651096	-1.241916
7	1	0	-4.656963	-0.074064	1.278831
8	1	0	-4.784816	0.142812	-0.479677
9	1	0	-5.222569	-1.408525	0.257935
10	7	0	-2.215240	0.050187	0.436477
11	7	0	-1.343696	0.381587	-0.368480
12	1	0	-0.961779	-0.088596	-1.386087
13	8	0	-0.177798	-0.453363	-2.428077
14	1	0	-0.782757	1.208116	-0.055101
15	1	0	0.839773	-0.156151	-2.144148
16	1	0	-0.412256	-0.008822	-3.250858
17	8	0	2.024363	0.264523	-1.611239
18	1	0	2.760895	-0.023526	-2.162448
19	1	0	-2.886320	-1.762257	0.975028
20	8	0	1.657047	-1.796409	0.261079
21	1	0	1.541642	-1.342616	1.114223

22	1	0	1.819425	-1.085865	-0.401873
23	1	0	-1.615786	1.001150	2.375989
24	8	0	-0.869147	1.451978	2.799880
25	1	0	-0.637843	2.158204	2.173907
26	8	0	0.194166	2.681089	0.397621
27	1	0	0.330318	3.031775	-0.504762
28	1	0	1.097893	2.349218	0.622898
29	8	0	1.161252	2.808088	-2.227902
30	1	0	1.578958	1.923526	-2.067923
31	1	0	1.874815	3.394507	-2.500628
32	1	0	2.546130	1.027971	-0.093084
33	8	0	2.650882	1.528350	0.753082
34	1	0	2.447569	0.897295	1.465153
35	8	0	3.490234	-3.866691	0.184979
36	1	0	4.311740	-3.481256	0.505946
37	1	0	2.839365	-3.131700	0.212053
38	8	0	1.381252	-0.203271	2.653158
39	1	0	0.566812	0.326099	2.806334
40	1	0	1.684531	-0.500160	3.518431

%chk=wk03e.high.chk

Standard basis: 6-311+G(d,p) (6D, 7F)

478 basis functions

65 alpha electrons 65 beta electrons

nuclear repulsion energy 1150.9845402492 Hartrees.

NAtoms= 40

Polarizable Continuum Model (PCM)

=====================================

Model : PCM.

Atomic radii : UFF (Universal Force Field).

Polarization charges : Total charges.

Solvent : 1,2-EthaneDiol, Eps= 40.245000 Eps(inf)= 2.050051

SCF Done: E(RB3LYP) = -916.919942551 A.U. after 1 cycles

Convg = 0.4258D-08 -V/T = 2.0047

	1	2	3
	A	A	A
Frequencies --	-721.2777	11.9011	28.7607

Zero-point correction= 0.332962 (a.u.)

Thermal correction to Energy= 0.362080

Thermal correction to Enthalpy= 0.363024

Thermal correction to Gibbs Free Energy= 0.271355

Sum of electronic and zero-point Energies= -916.586981

Sum of electronic and thermal Energies= -916.557863

Sum of electronic and thermal Enthalpies= -916.556919

Sum of electronic and thermal Free Energies= -916.648588

	E (Thermal)	CV	S
	KCal/Mol	Cal/Mol-Kelvin	Cal/Mol-Kelvin
Total	227.208	98.571	192.934

(x) trans-di-imine

Stoichiometry C3H26N2O9

Standard orientation:

Center Number	Atomic Number	Atomic Type	Coordinates (Angstroms) X	Y	Z
1	6	0	-3.109576	0.182367	-1.043895
2	6	0	-3.046978	1.580115	-1.647095
3	6	0	-4.541729	-0.272433	-0.726059
4	1	0	-3.450015	2.327891	-0.959714
5	1	0	-2.026195	1.866796	-1.904218
6	1	0	-3.644746	1.598300	-2.561169
7	1	0	-4.549406	-1.276885	-0.298326
8	1	0	-5.013390	0.411828	-0.015433
9	1	0	-5.136457	-0.281953	-1.641337
10	7	0	-2.317444	-0.026909	0.190361
11	7	0	-1.703363	0.949703	0.637063
12	1	0	-0.544825	2.416725	-0.122111
13	8	0	0.239008	2.821226	-0.536619
14	1	0	-1.213193	0.647188	1.496736
15	1	0	1.640913	1.580161	-0.602613
16	1	0	0.703508	3.319571	0.157691
17	8	0	2.490621	1.168691	-0.342432

18	1	0	2.832732	0.698286	-1.134305
19	1	0	-2.677296	-0.544685	-1.743286
20	8	0	1.816803	-2.658736	-2.191615
21	1	0	1.482264	-2.818256	-1.283272
22	1	0	1.070172	-2.809922	-2.781200
23	1	0	-1.828456	-1.765778	0.999299
24	8	0	-1.328783	-2.394092	1.560707
25	1	0	-0.988186	-1.816913	2.272286
26	8	0	-0.136364	-0.308904	2.996104
27	1	0	-0.049598	-0.060484	3.922560
28	1	0	0.778568	-0.385440	2.626421
29	8	0	2.439992	3.573830	1.140328
30	1	0	2.826872	2.810450	0.674902
31	1	0	3.038767	4.311143	0.979729
32	1	0	2.311488	-0.058110	0.971429
33	8	0	2.198192	-0.724702	1.683772
34	1	0	1.963883	-1.555779	1.230519
35	8	0	3.261183	-0.338058	-2.519914
36	1	0	4.184692	-0.569566	-2.665880
37	1	0	2.776625	-1.189475	-2.433249
38	8	0	1.065757	-3.066546	0.452920
39	1	0	0.151299	-2.900672	0.798212
40	1	0	1.324063	-3.935953	0.780644

%chk=wk03e.for.high.chk

Standard basis: 6-311+G(d,p) (6D, 7F)

478 basis functions

65 alpha electrons 65 beta electrons

nuclear repulsion energy 1101.6784447336 Hartrees.

NAtoms= 40 NActive= 40

Polarizable Continuum Model (PCM)

==================================

Model : PCM.

Atomic radii : UFF (Universal Force Field).

Solvent : 1,2-EthaneDiol, Eps= 40.245000 Eps(inf)= 2.050051

SCF Done: E(RB3LYP) = -916.956840726 A.U. after 1 cycles

Convg = 0.1852D-08 -V/T = 2.0046

Zero-point correction= 0.338291 (a.u.)

Thermal correction to Energy= 0.369066

Thermal correction to Enthalpy= 0.370010

Thermal correction to Gibbs Free Energy= 0.273966

Sum of electronic and zero-point Energies= -916.618550

Sum of electronic and thermal Energies= -916.587775

Sum of electronic and thermal Enthalpies= -916.586831

Sum of electronic and thermal Free Energies= -916.682875

	E (Thermal)	CV	S
	KCal/Mol	Cal/Mol-Kelvin	Cal/Mol-Kelvin
Total	231.592	102.725	202.141

(xi) TS5

Stoichiometry C3H26N2O9

Standard orientation:

Center Number	Atomic Number	Atomic Type	Coordinates (Angstroms) X	Y	Z
1	6	0	-3.305691	1.108338	0.048221
2	6	0	-2.718684	2.308171	0.722368
3	6	0	-4.403948	0.351185	0.767925
4	1	0	-2.677625	2.182920	1.811236
5	1	0	-1.678633	2.464484	0.367599
6	1	0	-3.238984	3.260260	0.527802
7	1	0	-4.594780	-0.628617	0.310368
8	1	0	-4.164880	0.179180	1.824914
9	1	0	-5.374018	0.882010	0.766319
10	7	0	-1.536434	0.144578	-0.925567
11	7	0	-0.661872	0.877187	-1.050824
12	1	0	0.219261	2.629262	-0.863132
13	8	0	0.852389	3.373873	-0.907845
14	1	0	1.017811	-0.025724	-1.713275
15	1	0	2.403554	3.066566	-0.134536
16	1	0	0.363131	4.154087	-0.624105
17	8	0	3.285226	2.824275	0.231133
18	1	0	3.300649	3.159451	1.134397

19	1	0	-3.586890	1.309071	-0.988600
20	1	0	-1.840085	-1.585358	-1.569258
21	8	0	-1.751020	-2.541497	-1.795587
22	1	0	-2.005306	-2.632421	-2.721534
23	8	0	1.744910	-0.668249	-1.843431
24	1	0	1.178592	-2.025656	-1.385370
25	1	0	2.481415	-0.359518	-1.270003
26	8	0	0.710882	-2.866786	-1.025598
27	1	0	0.644370	-2.741011	0.033529
28	1	0	-0.231632	-2.850024	-1.392248
29	8	0	0.550637	-2.494434	1.423368
30	1	0	-0.194240	-1.872612	1.601700
31	1	0	1.377943	-2.060318	1.742945
32	8	0	-1.524602	-0.773221	1.856594
33	1	0	-1.982336	-0.266022	1.147108
34	1	0	-2.211259	-1.038163	2.477603
35	8	0	3.727906	0.189586	-0.087495
36	1	0	4.637013	0.058730	-0.382439
37	1	0	3.616311	1.166048	0.050707
38	8	0	2.862292	-1.251195	2.141617
39	1	0	3.582221	-1.764231	2.524615
40	1	0	3.247518	-0.759728	1.385262

----

wk03ff.high.chk

Standard basis: 6-311+G(d,p) (6D, 7F)

478 basis functions

65 alpha electrons 65 beta electrons

nuclear repulsion energy 1115.3072930843 Hartrees.

NAtoms= 40 NActive= 40

Polarizable Continuum Model (PCM)

=====================================

Model : PCM.

Atomic radii : UFF (Universal Force Field).

Solvent: 1,2-EthaneDiol, Eps= 40.245000 Eps(inf)= 2.050051

SCF Done: E(RB3LYP) = -916.883907334 A.U. after 1 cycles

Convg = 0.5147D-08 -V/T = 2.0047

	1	2	3
	A	A	A
Frequencies --	-126.0049	20.8467	26.0072

Zero-point correction= 0.330805 (a.u.)

Thermal correction to Energy= 0.360711

Thermal correction to Enthalpy= 0.361656

Thermal correction to Gibbs Free Energy= 0.269279

Sum of electronic and zero-point Energies= -916.553103

Sum of electronic and thermal Energies= -916.523196

Sum of electronic and thermal Enthalpies= -916.522252

Sum of electronic and thermal Free Energies= -916.614629

E (Thermal) CV S

	KCal/Mol	Cal/Mol-Kelvin	Cal/Mol-Kelvin
Total	226.350	100.666	194.423

(xii) propane product

Stoichiometry C3H26N2O9

Standard orientation:

Center Number	Atomic Number	Atomic Type	Coordinates (Angstroms) X	Y	Z
1	6	0	7.984412	-0.123793	0.375987
2	6	0	7.409374	0.439126	-0.927361
3	6	0	9.058295	-1.191042	0.143465
4	1	0	6.949320	-0.352184	-1.528103
5	1	0	6.645638	1.197743	-0.734015
6	1	0	8.193439	0.902115	-1.535275
7	1	0	9.452032	-1.575978	1.088467
8	1	0	8.654747	-2.038624	-0.419842
9	1	0	9.899319	-0.784638	-0.427570
10	7	0	-0.465282	4.564339	-0.761253
11	7	0	0.563724	4.380257	-0.434567
12	1	0	0.104819	0.374122	-1.223828
13	8	0	-0.514952	-0.276683	-0.875160
14	1	0	-2.357588	0.001823	-0.985317
15	1	0	-0.523613	-1.961063	-1.564123

16	1	0	-0.351875	-0.339798	0.104953
17	8	0	-0.780191	-2.864165	-1.848737
18	1	0	-0.061151	-3.444995	-1.576625
19	1	0	8.407520	0.694251	0.969838
20	1	0	-4.559294	1.012853	-3.713398
21	8	0	-4.836046	1.314220	-2.841954
22	1	0	-4.289953	0.804594	-2.211796
23	8	0	-3.330746	-0.096077	-0.918748
24	1	0	-3.813388	0.389894	0.737106
25	1	0	-3.467263	-1.065666	-0.914041
26	8	0	-3.937133	0.477968	1.710910
27	1	0	-2.533716	1.371202	2.497408
28	1	0	-4.843125	0.776659	1.849149
29	8	0	-1.646803	1.668179	2.787331
30	1	0	-0.643255	0.222163	2.214722
31	1	0	-1.721956	1.843706	3.731652
32	8	0	-0.330161	-0.601504	1.787532
33	1	0	7.173164	-0.549382	0.977340
34	1	0	-1.064882	-1.234681	1.936541
35	8	0	-3.209160	-2.900040	-0.615423
36	1	0	-3.835226	-3.570170	-0.911779
37	1	0	-2.360650	-3.066107	-1.090195
38	8	0	-2.699665	-2.112584	2.088495
39	1	0	-2.903848	-2.514419	1.223180
40	1	0	-3.271932	-1.325844	2.129770

%chk=wk03ff.rev.high.chk

Standard basis: 6-311+G(d,p) (6D, 7F)

478 basis functions

65 alpha electrons 65 beta electrons

nuclear repulsion energy 936.8190732642 Hartrees.

NAtoms= 40 NActive= 40

Polarizable Continuum Model (PCM)

==================================

Model : PCM.

Solvent: 1,2-EthaneDiol, Eps= 40.245000 Eps(inf)= 2.050051

SCF Done: E(RB3LYP) = -917.028921394 A.U. after 1 cycles

Convg = 0.6372D-08 -V/T = 2.0047

Zero-point correction= 0.332356 (a.u.)

Thermal correction to Energy= 0.367060

Thermal correction to Enthalpy= 0.368004

Thermal correction to Gibbs Free Energy= 0.243486

Sum of electronic and zero-point Energies= -916.696565

Sum of electronic and thermal Energies= -916.661861

Sum of electronic and thermal Enthalpies= -916.660917

Sum of electronic and thermal Free Energies= -916.785436

	E (Thermal)	CV	S
	KCal/Mol	Cal/Mol-Kelvin	Cal/Mol-Kelvin
Total	230.334	107.192	262.071

[2] Geometries and energies in Figure 3

I(Me) Precursor

Stoichiometry C3H25N2O9(1-)

Standard orientation:

Center Number	Atomic Number	Atomic Type	Coordinates (Angstroms) X	Y	Z
1	6	0	3.752382	0.512195	-0.373585
2	8	0	2.799271	-0.258636	-0.356215
3	6	0	5.017975	0.216046	0.385248
4	6	0	3.702149	1.795906	-1.156335
5	1	0	5.029564	-0.811646	0.745026
6	1	0	5.894004	0.409139	-0.238506
7	1	0	5.080279	0.897588	1.240360
8	1	0	2.694100	1.994523	-1.516293
9	1	0	4.059504	2.630193	-0.547531
10	1	0	4.382752	1.712228	-2.010262
11	7	0	-0.489664	1.829392	1.324504
12	7	0	-1.034429	3.079908	1.818884
13	1	0	-0.997424	1.075438	1.782988
14	1	0	-0.698669	1.730898	0.330337

15	1	0	-1.802468	3.374077	1.212251
16	1	0	-0.303179	3.778710	1.734197
17	8	0	-1.898229	1.104680	-1.568985
18	1	0	-2.624704	2.405895	-0.919750
19	1	0	-2.058024	1.204199	-2.514342
20	8	0	-3.051185	3.218362	-0.496018
21	1	0	-3.977175	2.986997	-0.372315
22	8	0	-2.121079	-0.871300	2.048599
23	1	0	-1.460016	-1.461555	1.641933
24	1	0	-2.726755	-0.688596	1.299282
25	8	0	-3.580855	-0.574017	-0.355037
26	1	0	-2.969244	0.029871	-0.874682
27	1	0	-3.390293	-1.481033	-0.650619
28	1	0	-0.635226	0.123736	-1.506530
29	8	0	0.088769	-0.588328	-1.482389
30	1	0	0.919891	-0.180739	-1.197367
31	1	0	-0.253001	-1.777283	-0.319603
32	8	0	-0.432443	-2.479469	0.361471
33	1	0	0.456793	-2.776343	0.633031
34	1	0	2.602043	-1.918030	0.501149
35	8	0	2.339995	-2.791595	0.853133
36	1	0	2.689398	-2.824333	1.750446
37	1	0	-3.254696	-4.000790	-0.329778
38	8	0	-2.759163	-3.347449	-0.835047
39	1	0	-1.904502	-3.241116	-0.365203

%chk=wk02hb.high.chk

Standard basis: 6-311+G(d,p) (6D, 7F)

472 basis functions

65 alpha electrons 65 beta electrons

nuclear repulsion energy 1075.6351834992 Hartrees.

NAtoms= 39 NActive= 39

Polarizable Continuum Model (PCM)

=================================

Model : PCM.

Atomic radii : UFF (Universal Force Field).

Solvent : 1,2-EthaneDiol, Eps= 40.245000 Eps(inf)= 2.050051

SCF Done: E(RB3LYP) = -916.483613380 A.U. after 1 cycles

Convg = 0.3124D-08 -V/T = 2.0047

Zero-point correction= 0.324237 (a.u.)

Thermal correction to Energy= 0.355383

Thermal correction to Enthalpy= 0.356328

Thermal correction to Gibbs Free Energy= 0.256964

Sum of electronic and zero-point Energies= -916.159376

Sum of electronic and thermal Energies= -916.128230

Sum of electronic and thermal Enthalpies= -916.127286

Sum of electronic and thermal Free Energies= -916.226649

E (Thermal) CV S

	KCal/Mol	Cal/Mol-Kelvin	Cal/Mol-Kelvin
Total	223.006	100.419	209.127

II(Me) Mulliken CT complex

Stoichiometry C3H25N2O9(1-)

Standard orientation:

Center Number	Atomic Number	Atomic Type	Coordinates (Angstroms) X	Y	Z
1	6	0	-2.124677	-0.971899	0.144954
2	8	0	-1.026637	-1.717194	0.391157
3	6	0	-2.900247	-1.424682	-1.100120
4	6	0	-3.041667	-0.858485	1.372512
5	1	0	-2.235750	-1.464777	-1.966821
6	1	0	-3.279512	-2.431861	-0.914629
7	1	0	-3.741578	-0.769337	-1.330767
8	1	0	-2.489714	-0.455820	2.225439
9	1	0	-3.929949	-0.246157	1.195109
10	1	0	-3.379258	-1.863778	1.631496
11	7	0	-1.590876	0.491583	-0.159902
12	7	0	-2.588358	1.495925	-0.481683
13	1	0	-0.972429	0.813805	0.625755
14	1	0	-0.961393	0.412413	-0.981502
15	1	0	-3.156470	1.612658	0.354351

16	1	0	-2.062333	2.371063	-0.583946
17	8	0	0.357056	1.732296	1.404916
18	1	0	-0.173535	2.998118	0.458632
19	1	0	0.398550	2.155610	2.269445
20	8	0	-0.576662	3.689332	-0.148835
21	1	0	0.111267	3.892893	-0.791290
22	8	0	0.479982	0.367005	-2.200526
23	1	0	0.702542	-0.575989	-2.074179
24	1	0	1.152888	0.866609	-1.682747
25	8	0	2.201227	1.793141	-0.546636
26	1	0	1.636320	1.782354	0.273444
27	1	0	3.003619	1.275509	-0.343612
28	1	0	0.783974	0.090923	1.928362
29	8	0	0.855389	-0.876434	2.131361
30	1	0	0.109243	-1.250170	1.591747
31	1	0	2.184458	-1.600135	1.220456
32	8	0	2.810591	-2.008254	0.572667
33	1	0	2.227806	-2.220365	-0.183759
34	1	0	-0.022236	-2.129358	-0.710718
35	8	0	0.757849	-2.303874	-1.353993
36	1	0	0.548028	-3.100643	-1.852372
37	1	0	5.045203	-0.094544	-0.688119
38	8	0	4.421920	0.107956	0.017477
39	1	0	3.908727	-0.722960	0.176539

%chk=wk02qx.high.chk

Standard basis: 6-311+G(d,p) (6D, 7F)

472 basis functions

65 alpha electrons 65 beta electrons

nuclear repulsion energy 1206.1691776630 Hartrees.

NAtoms= 39 NActive= 39

Polarizable Continuum Model (PCM)

=================================

Model : PCM.

Atomic radii : UFF (Universal Force Field).

Solvent: 1,2-EthaneDiol, Eps= 40.245000 Eps(inf)= 2.050051

SCF Done: E(RB3LYP) = -916.486500375 A.U. after 1 cycles

Convg = 0.1470D-08 -V/T = 2.0046

Zero-point correction= 0.330873 (a.u.)

Thermal correction to Energy= 0.357376

Thermal correction to Enthalpy= 0.358320

Thermal correction to Gibbs Free Energy= 0.276490

Sum of electronic and zero-point Energies= -916.155627

Sum of electronic and thermal Energies= -916.129124

Sum of electronic and thermal Enthalpies= -916.128180

Sum of electronic and thermal Free Energies= -916.210010

E (Thermal)	CV	S
KCal/Mol	Cal/Mol-Kelvin	Cal/Mol-Kelvin

Total	224.257	93.406	172.225

III(Me) TS1

Stoichiometry C3H25N2O9(1-)

Standard orientation:

Center Number	Atomic Number	Atomic Type	Coordinates (Angstroms) X	Y	Z
1	6	0	-1.785470	-1.090291	0.204359
2	8	0	-0.622290	-1.486135	0.920811
3	6	0	-2.333025	-2.257836	-0.617039
4	6	0	-2.789465	-0.585794	1.233964
5	1	0	-1.586954	-2.613922	-1.330887
6	1	0	-2.596720	-3.077693	0.053157
7	1	0	-3.233404	-1.974900	-1.166991
8	1	0	-2.369102	0.244719	1.803539
9	1	0	-3.712053	-0.253042	0.756480
10	1	0	-3.035869	-1.397341	1.920770
11	7	0	-1.335867	0.021990	-0.719017
12	7	0	-2.340301	0.729904	-1.465337
13	1	0	-0.825523	0.778193	-0.147559
14	1	0	-0.494666	-0.340127	-1.488050
15	1	0	-2.950440	0.065523	-1.930297
16	1	0	-2.883733	1.307062	-0.824684

17	8	0	-0.094903	2.070077	0.483929
18	1	0	-1.534117	2.747481	0.652446
19	1	0	0.306513	1.794891	1.316792
20	8	0	-2.496080	3.069174	0.685718
21	1	0	-2.488312	3.948877	0.295627
22	8	0	0.538979	-0.653531	-2.272138
23	1	0	1.126254	-1.610949	-1.437040
24	1	0	1.062739	0.164226	-2.219550
25	8	0	1.739187	1.993208	-1.494370
26	1	0	1.089294	2.055001	-0.736040
27	1	0	2.593947	1.769115	-1.086070
28	1	0	0.347926	-0.537854	2.133432
29	8	0	1.052460	-0.097963	2.654761
30	1	0	0.909764	-0.364861	3.569542
31	1	0	2.568825	-0.715150	1.722747
32	8	0	3.214398	-1.047363	1.068891
33	1	0	2.666164	-1.554892	0.429848
34	1	0	0.043268	-1.913271	0.325069
35	8	0	1.436914	-2.292558	-0.660496
36	1	0	1.564235	-3.166150	-1.045002
37	1	0	4.943680	0.900275	-0.775348
38	8	0	4.201894	1.163180	-0.220326
39	1	0	3.904746	0.340354	0.236595

%chk=wk02qq.high.chk

Standard basis: 6-311+G(d,p) (6D, 7F)

472 basis functions

65 alpha electrons 65 beta electrons

nuclear repulsion energy 1203.6046158942 Hartrees.

NAtoms= 39 NActive= 39

Polarizable Continuum Model (PCM)

=====================================

Model : PCM.

Atomic radii : UFF (Universal Force Field).

Solvent : 1,2-EthaneDiol, Eps= 40.245000 Eps(inf)= 2.050051

SCF Done: E(RB3LYP) = -916.467237904 A.U. after 1 cycles

Convg = 0.2125D-08 -V/T = 2.0047

	1	2	3
	A	A	A
Frequencies --	-688.3267	37.9183	44.9599

Zero-point correction= 0.323276 (a.u.)

Thermal correction to Energy= 0.349969

Thermal correction to Enthalpy= 0.350913

Thermal correction to Gibbs Free Energy= 0.267748

Sum of electronic and zero-point Energies= -916.143962

Sum of electronic and thermal Energies= -916.117269

Sum of electronic and thermal Enthalpies= -916.116325

Sum of electronic and thermal Free Energies= -916.199489

	E (Thermal)	CV	S
	KCal/Mol	Cal/Mol-Kelvin	Cal/Mol-Kelvin
Total	219.609	93.262	175.035

IV(Me) (Me)2C(OH)-NH-NH2 intermediate

%chk=wk02qq.for.high.chk

Stoichiometry C3H25N2O9(1-)

Standard orientation:

Center Number	Atomic Number	Atomic Type	Coordinates (Angstroms) X	Y	Z
1	6	0	-2.277311	-0.530279	0.315595
2	8	0	-1.207579	-1.232877	0.962956
3	6	0	-3.238522	-1.530194	-0.328898
4	6	0	-2.960510	0.282681	1.418527
5	1	0	-2.721705	-2.115442	-1.093834
6	1	0	-3.626177	-2.209491	0.432668
7	1	0	-4.087660	-1.026190	-0.795188
8	1	0	-2.245814	0.965018	1.884041
9	1	0	-3.793476	0.867119	1.021470
10	1	0	-3.361286	-0.384455	2.184734
11	7	0	-1.636460	0.318276	-0.725134
12	7	0	-2.504139	1.171071	-1.482669

13	1	0	-0.927583	0.929216	-0.278828
14	1	0	-0.411434	-0.409059	-1.929354
15	1	0	-2.993650	0.612754	-2.173781
16	1	0	-3.205999	1.617816	-0.893870
17	8	0	0.551493	1.989048	0.286045
18	1	0	0.500589	3.534654	0.471318
19	1	0	0.772348	1.545377	1.113894
20	8	0	0.435413	4.552953	0.567158
21	1	0	1.293303	4.889646	0.291152
22	8	0	0.374462	-0.755805	-2.423235
23	1	0	0.594834	-2.087911	-1.423993
24	1	0	1.081272	-0.096272	-2.207181
25	8	0	2.148196	1.149771	-1.580576
26	1	0	1.553969	1.493599	-0.819064
27	1	0	2.918979	0.737284	-1.152497
28	1	0	0.031222	-0.590057	2.095026
29	8	0	0.845968	-0.354115	2.590231
30	1	0	0.639915	-0.489294	3.521638
31	1	0	2.101093	-1.432849	1.750917
32	8	0	2.641919	-1.981551	1.147535
33	1	0	1.994539	-2.339007	0.508438
34	1	0	-0.739914	-1.810915	0.326094
35	8	0	0.588489	-2.726608	-0.656426
36	1	0	0.454180	-3.609268	-1.018983
37	1	0	4.953913	-0.722622	-0.682459
38	8	0	4.285889	-0.230953	-0.193169

39 1 0 3.752805 -0.907237 0.286762

Standard basis: 6-311+G(d,p) (6D, 7F)

472 basis functions

65 alpha electrons 65 beta electrons

nuclear repulsion energy 1168.8828322706 Hartrees.

NAtoms= 39 NActive= 39

Polarizable Continuum Model (PCM)

=================================

Model : PCM.

Atomic radii : UFF (Universal Force Field).

Solvent : 1,2-EthaneDiol, Eps= 40.245000 Eps(inf)= 2.050051

SCF Done: E(RB3LYP) = -916.481088780 A.U. after 1 cycles

Convg = 0.5180D-08 -V/T = 2.0047

Zero-point correction= 0.328121 (a.u.)

Thermal correction to Energy= 0.356053

Thermal correction to Enthalpy= 0.356997

Thermal correction to Gibbs Free Energy= 0.269639

Sum of electronic and zero-point Energies= -916.152967

Sum of electronic and thermal Energies= -916.125036

Sum of electronic and thermal Enthalpies= -916.124091

Sum of electronic and thermal Free Energies= -916.211450

E (Thermal) CV S

	KCal/Mol	Cal/Mol-Kelvin	Cal/Mol-Kelvin
Total	223.427	95.848	183.861

V(Me) TS2

%chk=wk02qr.high.chk

Stoichiometry C3H25N2O9(1-)

Standard orientation:

Center Number	Atomic Number	Atomic Type	Coordinates (Angstroms) X	Y	Z
1	6	0	-2.472442	-0.217928	0.347182
2	8	0	-1.454838	-0.922229	1.068358
3	6	0	-3.502148	-1.204990	-0.203083
4	6	0	-3.097019	0.753982	1.352066
5	1	0	-3.024944	-1.904898	-0.893328
6	1	0	-3.945687	-1.767693	0.620263
7	1	0	-4.306163	-0.689991	-0.733016
8	1	0	-2.333663	1.422297	1.757619
9	1	0	-3.877751	1.358648	0.885328
10	1	0	-3.554038	0.203206	2.176818
11	7	0	-1.772736	0.465610	-0.774047
12	7	0	-2.561949	1.296707	-1.629655
13	1	0	-1.028044	1.049065	-0.390149

14	1	0	-0.399880	-0.552979	-1.973941
15	1	0	-3.048013	0.708160	-2.297116
16	1	0	-3.262614	1.828660	-1.114503
17	8	0	1.006028	1.932710	0.078224
18	1	0	1.509936	3.594019	0.231502
19	1	0	1.031818	1.420411	0.903262
20	8	0	1.742060	4.549636	0.318699
21	1	0	2.702673	4.579443	0.268133
22	8	0	0.389262	-1.050467	-2.255265
23	1	0	0.311445	-2.108199	-1.170437
24	1	0	1.275717	-0.336871	-2.019655
25	8	0	2.201867	0.492121	-1.734044
26	1	0	1.505176	1.374422	-0.614958
27	1	0	2.936095	0.006077	-1.332453
28	1	0	-0.138241	-0.198133	2.007156
29	8	0	0.731796	0.115755	2.345701
30	1	0	0.599474	0.353422	3.270247
31	1	0	1.856468	-1.341124	1.855223
32	8	0	2.273279	-2.102723	1.410131
33	1	0	1.575703	-2.425744	0.800498
34	1	0	-1.026608	-1.598203	0.495202
35	8	0	0.176204	-2.697442	-0.329340
36	1	0	-0.073688	-3.579620	-0.624283
37	1	0	4.791920	-1.854351	-0.553977
38	8	0	4.317885	-1.111198	-0.166583
39	1	0	3.627009	-1.511930	0.408436

Standard basis: 6-311+G(d,p) (6D, 7F)

472 basis functions

65 alpha electrons 65 beta electrons

nuclear repulsion energy 1162.3598604709 Hartrees.

NAtoms= 39 NActive= 39

Polarizable Continuum Model (PCM)

=================================

Model : PCM.

Atomic radii : UFF (Universal Force Field).

Solvent: 1,2-EthaneDiol, Eps= 40.245000 Eps(inf)= 2.050051

SCF Done: E(RB3LYP) = -916.477669633 A.U. after 1 cycles

Convg = 0.1980D-08 -V/T = 2.0047

	1	2	3
	A	A	A
Frequencies --	-637.2256	17.5979	30.9423

Zero-point correction= 0.323863 (a.u.)

Thermal correction to Energy= 0.351440

Thermal correction to Enthalpy= 0.352384

Thermal correction to Gibbs Free Energy= 0.264788

Sum of electronic and zero-point Energies= -916.153807

Sum of electronic and thermal Energies= -916.126230

Sum of electronic and thermal Enthalpies= -916.125285

Sum of electronic and thermal Free Energies= -916.212881

	E (Thermal)	CV	S
	KCal/Mol	Cal/Mol-Kelvin	Cal/Mol-Kelvin
Total	220.532	94.333	184.361

IV(Me) (Me)2C(OH)-NH-NH2 with the different OH- position

%chk=wk02q.rev.high.chk

C3H25N2O9-

Standard orientation:

Center	Atomic	Atomic	Coordinates (Angstroms)		
Number	Number	Type	X	Y	Z
1	6	0	-1.724976	-1.328577	-0.032941
2	8	0	-0.442190	-1.610814	0.504250
3	6	0	-1.999357	-2.155345	-1.288970
4	6	0	-2.735316	-1.639009	1.076173
5	1	0	-1.234571	-1.968582	-2.044808
6	1	0	-1.997963	-3.218033	-1.039149
7	1	0	-2.975429	-1.908910	-1.713849
8	1	0	-2.532972	-1.030320	1.961171
9	1	0	-3.763664	-1.460137	0.754238

10	1	0	-2.657877	-2.691636	1.354489
11	7	0	-1.682923	0.128058	-0.390032
12	7	0	-2.912473	0.783883	-0.720758
13	1	0	-1.296107	0.617010	0.413899
14	1	0	-0.356429	0.481903	-1.748169
15	1	0	-3.115542	0.623625	-1.701783
16	1	0	-3.697361	0.428354	-0.176534
17	8	0	0.464250	2.477981	1.337582
18	1	0	-1.216887	3.081059	0.761649
19	1	0	0.573973	1.629999	1.806036
20	8	0	-2.091618	3.373620	0.439578
21	1	0	-2.454330	2.603793	-0.040605
22	8	0	0.454928	0.514659	-2.301591
23	1	0	0.957368	-0.272407	-1.958282
24	1	0	1.450637	1.805633	-1.506347
25	8	0	2.070545	2.318493	-0.938894
26	1	0	1.080369	2.436837	0.571872
27	1	0	2.803254	1.681109	-0.809479
28	1	0	0.186096	-0.715613	1.871475
29	8	0	0.696515	-0.151592	2.502361
30	1	0	0.329734	-0.322230	3.377369
31	1	0	2.368988	-1.186259	1.991915
32	8	0	2.967726	-1.713635	1.439599
33	1	0	2.491974	-1.771154	0.575707
34	1	0	0.304671	-1.605332	-0.190369
35	8	0	1.672843	-1.540399	-1.019818

36	1	0	1.793566	-2.332223	-1.555589
37	1	0	3.047432	-0.523169	-1.002722
38	8	0	3.803857	0.099656	-0.810879
39	1	0	4.061896	-0.173076	0.080317

Standard basis: 6-311+G(d,p) (6D, 7F)

472 basis functions

65 alpha electrons 65 beta electrons

nuclear repulsion energy 1196.3614872017 Hartrees.

NAtoms= 39 NActive= 39

Polarizable Continuum Model (PCM)

=================================

Model : PCM.

Solvent : 1,2-EthaneDiol, Eps= 40.245000 Eps(inf)= 2.050051

SCF Done: E(RB3LYP) = -916.495607245 A.U. after 1 cycles

Convg = 0.2090D-08 -V/T = 2.0046

Zero-point correction= 0.331459 (a.u.)

Thermal correction to Energy= 0.358102

Thermal correction to Enthalpy= 0.359046

Thermal correction to Gibbs Free Energy= 0.276066

Sum of electronic and zero-point Energies= -916.164149

Sum of electronic and thermal Energies= -916.137506

Sum of electronic and thermal Enthalpies= -916.136562

Sum of electronic and thermal Free Energies= -916.219541

	E (Thermal)	CV	S
	KCal/Mol	Cal/Mol-Kelvin	Cal/Mol-Kelvin
Total	224.712	94.189	174.645

VII(Me) TS3

Stoichiometry C3H25N2O9(1-)

Standard orientation:

Center Number	Atomic Number	Atomic Type	Coordinates (Angstroms) X	Y	Z
1	6	0	-2.324626	-0.932000	-0.154609
2	8	0	-1.086599	-1.411411	1.241406
3	6	0	-2.821244	-2.315953	-0.468523
4	6	0	-3.211974	-0.069471	0.706956
5	1	0	-2.036021	-2.934544	-0.895751
6	1	0	-3.216422	-2.780981	0.432507
7	1	0	-3.627512	-2.234131	-1.204659
8	1	0	-2.731758	0.877533	0.953043
9	1	0	-4.131388	0.139475	0.152332
10	1	0	-3.471488	-0.590714	1.626304
11	7	0	-1.651141	-0.263573	-1.099048

12	7	0	-0.910920	-0.957465	-2.088723
13	1	0	-1.328536	0.680226	-0.856726
14	1	0	2.388433	-1.322554	-2.413865
15	1	0	0.098948	-0.873530	-1.898202
16	1	0	-1.101888	-0.508700	-2.979645
17	8	0	-0.310035	2.157884	-0.073288
18	1	0	-0.936245	3.797778	0.073734
19	1	0	-0.168019	1.655313	0.783794
20	8	0	-1.286602	4.714719	0.155735
21	1	0	-2.048779	4.744132	-0.431313
22	8	0	2.053176	-0.898304	-1.616568
23	1	0	1.656180	-1.909819	-0.521441
24	1	0	1.964441	0.738608	-1.690787
25	8	0	1.986377	1.738377	-1.573205
26	1	0	0.527636	2.072989	-0.582800
27	1	0	2.768524	1.856013	-1.017084
28	1	0	-0.638156	-0.475771	1.652313
29	8	0	-0.013326	0.629864	2.073920
30	1	0	-0.393088	0.949244	2.899097
31	1	0	1.541457	-0.101233	2.267946
32	8	0	2.371390	-0.647885	2.256465
33	1	0	2.112821	-1.422187	1.722958
34	1	0	-0.333921	-1.889787	0.832399
35	8	0	1.350871	-2.460830	0.292678
36	1	0	1.548449	-3.384094	0.104643
37	1	0	3.417662	-0.308105	-0.591372

38	8	0	4.016883	0.138771	0.053907
39	1	0	3.563083	-0.012832	0.905623

%chk=wk02qyy.high.chk

Standard basis: 6-311+G(d,p) (6D, 7F)

472 basis functions

65 alpha electrons   65 beta electrons

nuclear repulsion energy   1181.3125132961 Hartrees.

NAtoms=   39 NActive=   39

Polarizable Continuum Model (PCM)

=================================

Model   : PCM.

Solvent : 1,2-EthaneDiol, Eps= 40.245000 Eps(inf)=   2.050051

SCF Done: E(RB3LYP) = -916.464363704   A.U. after   1 cycles

Convg =   0.3162D-08   -V/T = 2.0046

	1	2	3
	A	A	A
Frequencies --	-436.2723	22.0728	33.3895

Zero-point correction=   0.324064 (a.u.)

Thermal correction to Energy=   0.351146

Thermal correction to Enthalpy=   0.352090

Thermal correction to Gibbs Free Energy=   0.266972

Sum of electronic and zero-point Energies= -916.140300

Sum of electronic and thermal Energies= -916.113217

Sum of electronic and thermal Enthalpies= -916.112273

Sum of electronic and thermal Free Energies= -916.197392

	E (Thermal)	CV	S
	KCal/Mol	Cal/Mol-Kelvin	Cal/Mol-Kelvin
Total	220.348	93.948	179.147

VIII(Me) acetone hydrazone

Stoichiometry C3H25N2O9(1-)

Standard orientation:

Center Number	Atomic Number	Atomic Type	Coordinates (Angstroms) X	Y	Z
1	6	0	-2.837004	-0.440319	-0.040661
2	8	0	-0.393380	0.099385	3.012497
3	6	0	-2.954897	-1.853272	0.463348
4	6	0	-3.408824	0.662435	0.803867
5	1	0	-3.144522	-2.540039	-0.365381
6	1	0	-2.020744	-2.168813	0.941088
7	1	0	-3.752234	-1.938411	1.202771
8	1	0	-3.275112	1.633138	0.325332
9	1	0	-4.477025	0.492865	0.976800

10	1	0	-2.920636	0.677384	1.783601
11	7	0	-2.269802	-0.120780	-1.146658
12	7	0	-1.741021	-1.173357	-1.928375
13	1	0	-1.006758	1.304993	-1.226527
14	1	0	0.931352	-3.099692	-1.281982
15	1	0	-0.988590	-1.670396	-1.432560
16	1	0	-1.308662	-0.739740	-2.736563
17	8	0	-0.136946	1.763008	-1.256068
18	1	0	0.146417	3.575515	-0.953824
19	1	0	0.567117	1.830783	0.390139
20	8	0	0.341824	4.415955	-0.496406
21	1	0	0.856782	4.936312	-1.121700
22	8	0	0.970780	-2.204730	-0.928021
23	1	0	0.924562	-2.144352	0.597325
24	1	0	1.162959	-0.960582	-1.966015
25	8	0	1.383155	-0.133315	-2.499680
26	1	0	0.456206	1.146073	-1.752938
27	1	0	2.332802	-0.038760	-2.343580
28	1	0	-0.014742	0.850043	2.524875
29	8	0	0.972541	1.989365	1.273703
30	1	0	1.069505	2.950487	1.306319
31	1	0	2.430341	0.764154	1.525038
32	8	0	2.985073	-0.027589	1.650652
33	1	0	2.334098	-0.749873	1.769612
34	1	0	-0.021518	-0.681032	2.559648
35	8	0	0.956815	-1.986410	1.619290

36	1	0	1.081510	-2.847447	2.032044
37	1	0	2.734654	-1.751083	-0.862115
38	8	0	3.620470	-1.315969	-0.838747
39	1	0	3.579702	-0.783222	-0.023259

%chk=wk02qyy.revfor.high.chk

Standard basis: 6-311+G(d,p) (6D, 7F)

472 basis functions

65 alpha electrons 65 beta electrons

nuclear repulsion energy 1143.6081095684 Hartrees.

NAtoms= 39 NActive= 39

Polarizable Continuum Model (PCM)

=====================================

Model : PCM (using non-symmetric T matrix).

Atomic radii : UFF (Universal Force Field).

Polarization charges : Total charges.

Solvent : 1,2-EthaneDiol, Eps= 40.245000 Eps(inf)= 2.050051

SCF Done: E(RB3LYP) = -916.495556649 A.U. after 1 cycles

NFock= 1 Conv=0.24D-08 -V/T= 2.0046

Zero-point correction= 0.324210 (a.u.)

Thermal correction to Energy= 0.354207

Thermal correction to Enthalpy= 0.355151

Thermal correction to Gibbs Free Energy= 0.261181

Sum of electronic and zero-point Energies= -916.171347

Sum of electronic and thermal Energies= -916.141350

Sum of electronic and thermal Enthalpies= -916.140406

Sum of electronic and thermal Free Energies= -916.234376

	E (Thermal)	CV	S
	KCal/Mol	Cal/Mol-Kelvin	Cal/Mol-Kelvin
Total	222.268	100.611	197.778

IX(Me) TS4

%chk=wk03kx.high.freq.chk

Stoichiometry C3H25N2O9(1-)

Standard orientation:

Center Number	Atomic Number	Atomic Type	Coordinates (Angstroms) X	Y	Z
1	6	0	1.715000	-1.035866	1.161097
2	6	0	0.435274	-1.799188	1.386049
3	6	0	2.791058	-1.122193	2.210657
4	1	0	0.420279	-2.259024	2.375984
5	1	0	-0.436410	-1.144361	1.292275
6	1	0	0.312810	-2.591410	0.639167

7	1	0	3.672031	-0.547700	1.919355
8	1	0	2.435955	-0.746439	3.178462
9	1	0	3.098134	-2.162577	2.375144
10	7	0	1.931079	-0.319855	0.109652
11	7	0	0.954267	-0.210271	-0.864350
12	1	0	-0.205251	-1.197035	-1.397019
13	8	0	-0.938033	-1.808227	-1.808829
14	1	0	1.395408	0.323677	-1.612270
15	1	0	-1.058483	-1.491077	-2.711893
16	1	0	0.638821	-3.033586	-2.064590
17	8	0	1.605418	-3.143480	-2.075129
18	1	0	1.889347	-2.285708	-1.724548
19	1	0	-0.123414	1.061950	-0.408810
20	8	0	-0.662149	1.892026	-0.178831
21	1	0	-1.466576	1.850460	-0.737230
22	1	0	-0.800215	1.405340	2.899330
23	8	0	-1.583353	1.555995	2.358872
24	1	0	-1.247585	1.678509	1.433846
25	1	0	-2.734645	0.195011	2.249852
26	8	0	-3.387525	-0.514427	2.050811
27	1	0	-3.208688	-1.221073	2.680492
28	1	0	-2.516271	-1.368295	-1.007165
29	8	0	-3.377068	-0.994332	-0.716560
30	1	0	-3.338798	-0.919702	0.258811
31	1	0	-3.120057	1.622037	-2.534684
32	8	0	-3.097210	1.590635	-1.572192

33	1	0	-3.280034	0.650785	-1.326697
34	1	0	3.109937	0.948758	-0.078520
35	8	0	3.634952	1.777705	-0.295000
36	1	0	3.973107	2.097046	0.548605
37	8	0	1.522290	3.474531	-1.226535
38	1	0	2.272740	2.923429	-0.930777
39	1	0	0.733872	3.021943	-0.873892

Standard basis: 6-311+G(d,p) (6D, 7F)

472 basis functions

65 alpha electrons 65 beta electrons

nuclear repulsion energy 1122.6628397580 Hartrees.

NAtoms= 39 NActive= 39

Polarizable Continuum Model (PCM)

=================================

Model : PCM.

Atomic radii : UFF (Universal Force Field).

Solvent : 1,2-EthaneDiol, Eps= 40.245000 Eps(inf)= 2.050051

SCF Done: E(RB3LYP) = -916.477329743 A.U. after 13 cycles

Convg = 0.5305D-08 -V/T = 2.0046

	1	2	3
	A	A	A
Frequencies --	-102.3240	25.4089	33.2765

Zero-point correction= 0.321970 (a.u.)

Thermal correction to Energy= 0.351597

Thermal correction to Enthalpy= 0.352541

Thermal correction to Gibbs Free Energy= 0.259774

Sum of electronic and zero-point Energies= -916.155360

Sum of electronic and thermal Energies= -916.125733

Sum of electronic and thermal Enthalpies= -916.124789

Sum of electronic and thermal Free Energies= -916.217556

	E (Thermal)	CV	S
	KCal/Mol	Cal/Mol-Kelvin	Cal/Mol-Kelvin
Total	220.630	98.976	195.244

X(Me) anion intermediate

Stoichiometry C3H25N2O9(1-)

Standard orientation:

Center Number	Atomic Number	Atomic Type	Coordinates (Angstroms) X	Y	Z
1	6	0	2.262220	-0.791910	1.154739
2	6	0	1.197755	-1.654290	1.782277
3	6	0	3.481945	-0.460501	1.972959
4	1	0	1.459928	-1.919428	2.808038

5	1	0	0.230620	-1.140088	1.791281
6	1	0	1.048581	-2.577924	1.212918
7	1	0	4.181006	0.158940	1.409002
8	1	0	3.210488	0.073149	2.892257
9	1	0	4.005695	-1.372219	2.285809
10	7	0	2.171348	-0.327195	-0.044635
11	7	0	1.049393	-0.620270	-0.806156
12	1	0	-0.467507	-1.560902	-0.664395
13	8	0	-1.273793	-2.145327	-0.755104
14	1	0	1.230569	-0.173667	-1.705397
15	1	0	-0.934463	-2.913805	-1.231236
16	1	0	1.946347	-3.738021	-1.428888
17	8	0	1.286056	-3.223343	-1.904605
18	1	0	1.289441	-2.322414	-1.476103
19	1	0	-0.073427	0.728987	-0.344556
20	8	0	-0.607334	1.554263	-0.142219
21	1	0	-1.313793	1.587601	-0.822861
22	1	0	-1.407442	1.291586	2.935312
23	8	0	-2.005775	1.452234	2.197913
24	1	0	-1.440598	1.481502	1.386320
25	1	0	-3.401087	0.378305	1.970395
26	8	0	-4.180057	-0.169524	1.721505
27	1	0	-4.215089	-0.883690	2.366927
28	1	0	-2.857464	-1.372580	-0.925652
29	8	0	-3.688898	-0.849565	-0.963737
30	1	0	-3.918692	-0.671248	-0.028789

31	1	0	-2.648969	1.501724	-2.853873
32	8	0	-2.780944	1.537805	-1.900483
33	1	0	-3.176176	0.667504	-1.644210
34	1	0	3.118380	0.951903	-0.737867
35	8	0	3.481739	1.764970	-1.205408
36	1	0	4.362850	1.909131	-0.844904
37	8	0	1.365876	3.581418	-0.620324
38	1	0	2.143432	3.029393	-0.830397
39	1	0	0.655712	2.935268	-0.443738

%chk=wk03kx.for.high.chk

Standard basis: 6-311+G(d,p) (6D, 7F)

472 basis functions

65 alpha electrons 65 beta electrons

nuclear repulsion energy 1104.2349984172 Hartrees.

NAtoms= 39 NActive= 39

Polarizable Continuum Model (PCM)

=================================

Model : PCM.

Atomic radii : UFF (Universal Force Field).

Solvent: 1,2-EthaneDiol, Eps= 40.245000 Eps(inf)= 2.050051

SCF Done: E(RB3LYP) = -916.481209059 A.U. after 1 cycles

Convg = 0.1456D-08 -V/T = 2.0046

Zero-point correction= 0.322376 (a.u.)

Thermal correction to Energy= 0.353055

Thermal correction to Enthalpy= 0.353999

Thermal correction to Gibbs Free Energy= 0.257325

Sum of electronic and zero-point Energies= -916.158833

Sum of electronic and thermal Energies= -916.128155

Sum of electronic and thermal Enthalpies= -916.127210

Sum of electronic and thermal Free Energies= -916.223884

	E (Thermal)	CV	S
	KCal/Mol	Cal/Mol-Kelvin	Cal/Mol-Kelvin
Total	221.545	101.171	203.467

XI(Me) TS5

Stoichiometry C3H25N2O9(1-)

Standard orientation:

Center Number	Atomic Number	Atomic Type	Coordinates (Angstroms) X	Y	Z
1	6	0	0.380299	1.073949	-1.631888
2	6	0	1.144692	0.352473	-2.733397
3	6	0	0.523522	2.591985	-1.669995
4	1	0	2.166608	0.736820	-2.796301
5	1	0	1.196025	-0.724785	-2.574920
6	1	0	0.667425	0.512029	-3.711472

7	1	0	-0.095436	3.068895	-0.906646
8	1	0	1.562392	2.890620	-1.497661
9	1	0	0.225641	3.004941	-2.644674
10	7	0	-0.902184	0.660621	-1.341416
11	7	0	-1.279100	-0.515392	-1.685275
12	1	0	-0.286093	-2.070635	-1.303775
13	8	0	0.144198	-2.821981	-0.825264
14	1	0	-2.210182	-0.659545	-1.279035
15	1	0	1.102629	-2.634795	-0.850264
16	1	0	-0.216484	-2.326724	0.825223
17	8	0	-0.341310	-1.842090	1.678907
18	1	0	0.037486	-2.402235	2.366042
19	1	0	-2.537607	0.364868	1.752512
20	8	0	-2.845483	-0.545816	1.963240
21	1	0	-2.027443	-1.079452	1.932244
22	1	0	0.974368	0.674773	-0.408967
23	8	0	1.525225	0.288869	0.629411
24	1	0	0.875632	-0.309714	1.042652
25	1	0	2.439467	-1.021575	-0.166263
26	8	0	2.831638	-1.863598	-0.504905
27	1	0	3.328163	-1.620994	-1.294624
28	1	0	1.084817	1.734837	1.561819
29	8	0	0.660436	2.478909	2.057164
30	1	0	1.027617	3.283174	1.673503
31	1	0	3.114708	0.110406	1.571677
32	8	0	3.965831	-0.173225	1.965950

33	1	0	4.159956	-0.988875	1.487989
34	1	0	-1.669410	1.567330	0.154378
35	8	0	-1.950255	1.907021	1.037945
36	1	0	-1.113600	2.182516	1.463488
37	8	0	-4.276157	-0.976810	-0.376572
38	1	0	-4.751888	-0.145520	-0.479937
39	1	0	-3.789852	-0.883622	0.474525

%chk=wk03ky.high.chk

Standard basis: 6-311+G(d,p) (6D, 7F)

472 basis functions

65 alpha electrons 65 beta electrons

nuclear repulsion energy 1162.5624235790 Hartrees.

NAtoms= 39 NActive= 39

Polarizable Continuum Model (PCM)

=================================

Model : PCM.

Atomic radii : UFF (Universal Force Field).

Solvent: 1,2-EthaneDiol, Eps= 40.245000 Eps(inf)= 2.050051

SCF Done: E(RB3LYP) = -916.445905911 A.U. after 1 cycles

	1	2	3
	A	A	A
Frequencies --	-1326.8271	24.0572	37.5661

Zero-point correction= 0.321031(a.u.)

Thermal correction to Energy= 0.350026

Thermal correction to Enthalpy= 0.350970

Thermal correction to Gibbs Free Energy= 0.261894

Sum of electronic and zero-point Energies= -916.124875

Sum of electronic and thermal Energies= -916.095880

Sum of electronic and thermal Enthalpies= -916.094936

Sum of electronic and thermal Free Energies= -916.184012

	E (Thermal)	CV	S
	KCal/Mol	Cal/Mol-Kelvin	Cal/Mol-Kelvin
Total	219.644	98.539	187.477

XII(Me) di-imine

%chk=wk03ky.for.high.chk

Stoichiometry C3H25N2O9(1-)

Standard orientation:

Center	Atomic	Atomic	Coordinates (Angstroms)		
Number	Number	Type	X	Y	Z
1	6	0	2.677593	-0.783738	0.095810
2	6	0	2.938515	-1.536576	-1.203456

3	6	0	3.956707	-0.528484	0.905146
4	1	0	3.421611	-2.488644	-0.972045
5	1	0	2.016239	-1.746941	-1.746343
6	1	0	3.598493	-0.967626	-1.863195
7	1	0	3.736125	0.012153	1.827728
8	1	0	4.426777	-1.478890	1.165459
9	1	0	4.670337	0.059358	0.320980
10	7	0	1.995474	0.527256	-0.041893
11	7	0	1.658103	0.858365	-1.185863
12	1	0	0.129544	-0.178386	-2.382291
13	8	0	-0.755132	-0.503011	-2.609780
14	1	0	1.225314	1.798287	-1.127794
15	1	0	-0.799165	-1.414354	-2.239094
16	1	0	-2.094973	0.481557	-1.715948
17	8	0	-2.775562	0.961253	-1.201659
18	1	0	-3.253673	0.268196	-0.696112
19	1	0	-1.003670	2.229624	1.189771
20	8	0	-1.543653	2.741196	0.550674
21	1	0	-1.997772	2.083004	-0.021644
22	1	0	1.991331	-1.361176	0.728879
23	8	0	-1.369339	-1.636004	0.876614
24	1	0	-0.981491	-0.832902	0.508213
25	1	0	-1.149767	-2.540054	-0.412733
26	8	0	-1.044992	-2.949801	-1.338909
27	1	0	-0.310702	-3.570024	-1.284294
28	1	0	-0.934830	-1.345168	2.373554

29	8	0	-0.630328	-0.978338	3.273579
30	1	0	0.011194	-1.606955	3.620331
31	1	0	-2.909450	-1.337952	0.648440
32	8	0	-3.854860	-1.027037	0.427337
33	1	0	-4.297019	-1.778653	0.019118
34	1	0	0.981415	1.199965	1.592909
35	8	0	0.290243	1.442892	2.237650
36	1	0	0.021160	0.600353	2.667414
37	8	0	0.421968	3.637071	-1.113405
38	1	0	0.900876	4.372900	-0.717672
39	1	0	-0.316597	3.429989	-0.491145

Standard basis: 6-311+G(d,p) (6D, 7F)

472 basis functions

65 alpha electrons 65 beta electrons

nuclear repulsion energy 1128.2569858066 Hartrees.

NAtoms= 39 NActive= 39

Polarizable Continuum Model (PCM)

=================================

Model : PCM.

Atomic radii : UFF (Universal Force Field).

Polarization charges : Total charges.

Solvent: 1,2-EthaneDiol, Eps= 40.245000 Eps(inf)= 2.050051

SCF Done: E(RB3LYP) = -916.479460094 A.U. after 1 cycles

Convg = 0.8501D-08 -V/T = 2.0046

Zero-point correction= 0.324485 (a.u.)

Thermal correction to Energy= 0.353822

Thermal correction to Enthalpy= 0.354766

Thermal correction to Gibbs Free Energy= 0.262608

Sum of electronic and zero-point Energies= -916.154975

Sum of electronic and thermal Energies= -916.125638

Sum of electronic and thermal Enthalpies= -916.124694

Sum of electronic and thermal Free Energies= -916.216852

	E (Thermal)	CV	S
	KCal/Mol	Cal/Mol-Kelvin	Cal/Mol-Kelvin
Total	222.027	98.085	193.962

XIII(Me) TS6

Stoichiometry C3H25N2O9(1-)

Standard orientation:

Center Number	Atomic Number	Atomic Type	Coordinates (Angstroms) X	Y	Z
1	6	0	3.822847	-0.616210	0.169788
2	6	0	4.557293	-1.747862	-0.453250
3	6	0	3.725731	-0.605228	1.671628
4	1	0	5.397827	-2.139891	0.144826

5	1	0	3.861967	-2.615968	-0.598292
6	1	0	4.952838	-1.498423	-1.444306
7	1	0	3.111247	0.227485	2.036968
8	1	0	3.255614	-1.530185	2.042826
9	1	0	4.697888	-0.533947	2.197412
10	7	0	1.726910	-1.256855	-0.282054
11	7	0	1.667383	-2.364887	-0.574717
12	1	0	-0.122729	-2.775089	-0.860329
13	1	0	3.976874	0.362163	-0.288396
14	1	0	-2.154364	0.203167	-1.526084
15	8	0	-2.629508	-0.582597	-1.864171
16	1	0	-2.050133	-1.339826	-1.658865
17	8	0	-1.102567	-2.874883	-0.959194
18	1	0	-4.092241	-1.678478	0.625513
19	1	0	-1.237154	-3.705712	-1.430445
20	8	0	-4.486490	-0.880977	0.227087
21	1	0	-3.973754	-0.769745	-0.602969
22	8	0	-1.383758	1.602485	-0.521659
23	1	0	-0.403997	1.480315	-0.578520
24	1	0	-1.671775	1.261288	0.349627
25	8	0	1.310766	1.544665	-0.821232
26	1	0	1.778048	0.759628	-0.488174
27	1	0	1.660067	2.337015	-0.370393
28	8	0	-2.523976	-2.564986	1.481035
29	1	0	-2.219993	-1.707998	1.815477
30	1	0	-1.977296	-2.733863	0.688517

31	8	0	-2.600892	0.460643	1.745373
32	1	0	-2.869507	0.995251	2.500970
33	1	0	-3.427041	0.160480	1.303276
34	1	0	2.173553	4.763059	-0.094869
35	8	0	1.631250	4.082625	0.318216
36	1	0	0.700274	4.319979	0.116584
37	1	0	-1.327616	3.407681	-0.383416
38	8	0	-1.092670	4.347519	-0.212249
39	1	0	-1.411114	4.843652	-0.974186

%chk=wk03kf.high.chk

Standard basis: 6-311+G(d,p) (6D, 7F)

472 basis functions

65 alpha electrons 65 beta electrons

nuclear repulsion energy 1051.9965000015 Hartrees.

NAtoms= 39 NActive= 39

Polarizable Continuum Model (PCM)

==================================

Model : PCM.

Atomic radii : UFF (Universal Force Field).

Polarization charges : Total charges.

Solvent: 1,2-EthaneDiol, Eps= 40.245000 Eps(inf)= 2.050051

SCF Done: E(RB3LYP) = -916.439796033 A.U. after 1 cycles

Convg = 0.9602D-09 -V/T = 2.0046

	1	2	3
	A	A	A
Frequencies --	-170.6750	16.0448	23.9896

Zero-point correction= 0.319172 (a.u.)

Thermal correction to Energy= 0.350115

Thermal correction to Enthalpy= 0.351060

Thermal correction to Gibbs Free Energy= 0.254342

Sum of electronic and zero-point Energies= -916.120624

Sum of electronic and thermal Energies= -916.089681

Sum of electronic and thermal Enthalpies= -916.088736

Sum of electronic and thermal Free Energies= -916.185454

	E (Thermal)	CV	S
	KCal/Mol	Cal/Mol-Kelvin	Cal/Mol-Kelvin
Total	219.701	102.651	203.559

XIV(Me) propane product

Stoichiometry C3H25N2O9(1-)

Standard orientation:

Center	Atomic	Atomic	Coordinates (Angstroms)		
Number	Number	Type	X	Y	Z

1	6	0	4.946230	2.189288	0.204629
2	6	0	6.119640	2.927330	0.856330
3	6	0	3.789002	3.119720	-0.171298
4	1	0	5.805084	3.431015	1.776108
5	1	0	6.931728	2.241158	1.112997
6	1	0	6.526815	3.689868	0.184387
7	1	0	2.964385	2.567035	-0.630417
8	1	0	3.395481	3.634085	0.711387
9	1	0	4.114941	3.885313	-0.882816
10	1	0	4.581190	1.413060	0.886513
11	1	0	5.298228	1.666688	-0.691933
12	1	0	-0.179704	3.156979	-0.831876
13	8	0	-0.936634	3.007392	-1.412632
14	1	0	-0.898977	3.714974	-2.068580
15	8	0	-3.032830	-1.895544	0.335843
16	1	0	-3.467428	-2.743395	0.196137
17	1	0	-3.223042	-1.395237	1.784843
18	8	0	-3.305181	-0.999237	2.729177
19	1	0	-4.157431	-1.294705	3.065351
20	1	0	-1.502466	-1.894570	-0.245479
21	8	0	-0.578475	-1.808281	-0.639004
22	1	0	-0.662105	-1.098344	-1.302274
23	1	0	-3.551207	-0.759297	-0.692661
24	8	0	-3.771594	-0.030330	-1.358789
25	1	0	-3.814294	0.796889	-0.851453
26	1	0	-3.124877	0.802865	2.707057

27	8	0	-3.014770	1.777625	2.704429
28	1	0	-3.144533	2.046654	1.779956
29	1	0	0.230250	-3.262523	-1.155876
30	8	0	0.687970	-4.085940	-1.450088
31	1	0	1.474173	-4.149405	-0.898070
32	1	0	-1.030505	1.172961	-2.201315
33	8	0	-1.275777	0.281781	-2.496901
34	1	0	-2.207184	0.175734	-2.188280
35	1	0	-2.566289	2.820263	-0.520956
36	8	0	-3.394686	2.568734	-0.066754
37	1	0	-4.005226	3.302076	-0.204333
38	7	0	3.543496	-4.496606	0.358937
39	7	0	4.465016	-4.627360	0.935544

%chk=wk03kf.for.high.chk

Standard basis: 6-311+G(d,p) (6D, 7F)

472 basis functions

65 alpha electrons 65 beta electrons

nuclear repulsion energy 918.8201917012 Hartrees.

NAtoms= 39 NActive= 39

Polarizable Continuum Model (PCM)

=================================

Model : PCM (using non-symmetric T matrix).

Atomic radii : UFF (Universal Force Field).

Polarization charges : Total charges.

Solvent: 1,2-EthaneDiol, Eps= 40.245000 Eps(inf)=   2.050051

SCF Done: E(RB3LYP) = -916.546632617   A.U. after   2 cycles

NFock= 2 Conv=0.89D-08   -V/T= 2.0047

DoSCS=F DFT=T ScalE2(SS,OS)= 1.000000 1.000000

Zero-point correction=   0.317231 (a.u.)

Thermal correction to Energy=   0.351322

Thermal correction to Enthalpy=   0.352266

Thermal correction to Gibbs Free Energy=   0.234417

Sum of electronic and zero-point Energies=   -916.229402

Sum of electronic and thermal Energies=   -916.195311

Sum of electronic and thermal Enthalpies=   -916.194367

Sum of electronic and thermal Free Energies=   -916.312215

	E (Thermal)	CV	S
	KCal/Mol	Cal/Mol-Kelvin	Cal/Mol-Kelvin
Total	220.458	104.988	248.034

[3] Geometries and energies in Figure 5

Figure 5-1) trans-di-imine and DEG

Stoichiometry   C7H29N2O9(1-)

wkdeg1.highfor.log

Standard orientation:

Center Number	Atomic Number	Atomic Type	Coordinates (Angstroms) X	Y	Z
1	6	0	-4.000571	-1.123366	-0.393185
2	6	0	-4.115175	-0.432863	-1.754797
3	6	0	-4.824283	-0.420377	0.689291
4	1	0	-3.775530	0.604827	-1.698733
5	1	0	-3.519697	-0.951975	-2.509530
6	1	0	-5.157797	-0.434106	-2.081263
7	7	0	-2.576011	-1.103227	0.019127
8	7	0	-2.067177	-2.217502	0.184236
9	1	0	-1.062871	-2.115444	0.472681
10	8	0	0.710659	-2.423430	0.956030
11	1	0	0.600552	-3.204673	1.510094
12	1	0	-4.721516	-0.927141	1.651836
13	1	0	-4.308301	-2.171521	-0.476533
14	1	0	2.019344	-1.732205	1.396755
15	8	0	2.911919	-1.279577	1.650542
16	6	0	2.787456	0.126318	1.496791
17	1	0	-4.510461	0.619992	0.809472
18	1	0	-5.880511	-0.428007	0.410069
19	6	0	1.505866	0.659474	2.134690
20	1	0	2.772078	0.400574	0.433153
21	1	0	3.669650	0.595478	1.941242
22	8	0	1.228392	1.939160	1.552122
23	1	0	0.699700	-0.045932	1.918342

24	1	0	1.605286	0.759513	3.223076
25	6	0	0.013054	2.555514	1.986175
26	6	0	-1.256443	1.764879	1.690156
27	1	0	0.053377	2.748650	3.066589
28	1	0	-0.017566	3.517306	1.469819
29	8	0	-1.385267	1.478580	0.294070
30	1	0	-1.278076	0.833379	2.262546
31	1	0	-2.101781	2.375950	2.031378
32	1	0	-1.795520	0.587387	0.192216
33	1	0	1.060953	-2.865717	-0.556976
34	8	0	1.351738	-3.077256	-1.505416
35	1	0	1.246987	-2.242718	-1.989400
36	1	0	3.144416	-2.592979	-1.141629
37	8	0	3.895390	-2.006999	-0.912960
38	1	0	3.722938	-1.787207	0.025875
39	1	0	-0.017192	1.703755	-0.915708
40	8	0	0.887213	1.886892	-1.250083
41	1	0	1.352942	2.015821	-0.403097
42	1	0	1.673806	0.378824	-2.112749
43	8	0	2.063571	-0.395156	-2.561648
44	1	0	2.810615	-0.682008	-2.007068
45	1	0	0.992562	3.318163	-2.385480
46	8	0	1.073484	4.050401	-3.029892
47	1	0	0.667711	4.808727	-2.597367

Standard basis: 6-311+G(d,p) (6D, 7F)

588 basis functions

79 alpha electrons 79 beta electrons

nuclear repulsion energy 1607.8245471377 Hartrees.

NAtoms= 47 NActive= 47

Polarizable Continuum Model (PCM)

=====================================

Model : PCM.

Atomic radii : UFF (Universal Force Field).

Solvent: 1,2-EthaneDiol, Eps= 40.245000 Eps(inf)= 2.050051

SCF Done: E(RB3LYP) = -1071.28043468 A.U. after 1 cycles

Convg = 0.2894D-08 -V/T = 2.0048

Zero-point correction= 0.398346 (a.u.)

Thermal correction to Energy= 0.429752

Thermal correction to Enthalpy= 0.430696

Thermal correction to Gibbs Free Energy= 0.332489

Sum of electronic and zero-point Energies= -1070.882089

Sum of electronic and thermal Energies= -1070.850683

Sum of electronic and thermal Enthalpies= -1070.849739

Sum of electronic and thermal Free Energies= -1070.947946

	E (Thermal)	CV	S
	KCal/Mol	Cal/Mol-Kelvin	Cal/Mol-Kelvin
Total	269.673	106.040	206.695

Figure 5-2) TS6'

Stoichiometry C7H29N2O9(1-)

Standard orientation:

Center Number	Atomic Number	Atomic Type	Coordinates (Angstroms) X	Y	Z
1	6	0	-3.998609	-1.270685	-0.981353
2	6	0	-4.406727	0.016005	-1.654095
3	6	0	-4.489807	-1.584482	0.389488
4	1	0	-4.419423	0.856392	-0.948388
5	1	0	-3.712769	0.287034	-2.465686
6	1	0	-5.411944	-0.002703	-2.116929
7	7	0	-1.846218	-1.537996	-0.793551
8	7	0	-1.571040	-2.197592	0.112795
9	1	0	0.165850	-2.653401	0.312284
10	8	0	1.134686	-2.873251	0.389229
11	1	0	1.171921	-3.771932	0.738823
12	1	0	-3.679536	-2.108418	0.953579
13	1	0	-4.023606	-2.124843	-1.663175
14	1	0	2.258500	-1.705342	1.332153
15	8	0	2.914550	-1.131606	1.778481
16	6	0	2.447142	0.217652	1.753477
17	1	0	-4.741457	-0.674604	0.949407

18	1	0	-5.366996	-2.249116	0.450868
19	6	0	1.016435	0.345859	2.267734
20	1	0	2.484776	0.614350	0.733265
21	1	0	3.135876	0.801590	2.365768
22	8	0	0.535596	1.628305	1.855624
23	1	0	0.405076	-0.451851	1.833124
24	1	0	0.970415	0.261132	3.359948
25	6	0	-0.803260	1.940413	2.262443
26	6	0	-1.885105	1.088984	1.616514
27	1	0	-0.890552	1.847211	3.351928
28	1	0	-0.944899	2.989582	1.995898
29	8	0	-1.894824	1.275503	0.195402
30	1	0	-1.748173	0.030320	1.852391
31	1	0	-2.845434	1.401381	2.045045
32	1	0	-2.315915	0.500387	-0.211500
33	1	0	1.937967	-2.664444	-1.308218
34	8	0	2.457774	-2.435944	-2.102180
35	1	0	2.191890	-1.522839	-2.320229
36	1	0	3.989597	-1.667570	-1.187078
37	8	0	4.489970	-0.985122	-0.699877
38	1	0	4.168297	-1.072305	0.214657
39	1	0	-0.353571	1.770603	-0.829337
40	8	0	0.567970	2.086078	-0.911993
41	1	0	0.832881	2.075983	0.028131
42	1	0	1.773924	0.970623	-1.860639
43	8	0	2.418742	0.402436	-2.325228

44	1	0	3.174212	0.299849	-1.722621
45	1	0	0.706567	3.752201	-1.685282
46	8	0	0.828771	4.617962	-2.123463
47	1	0	0.101038	5.162476	-1.806095

%chk=wkdeg1.high.chk

Standard basis: 6-311+G(d,p) (6D, 7F)

588 basis functions

79 alpha electrons 79 beta electrons

nuclear repulsion energy 1593.0823070876 Hartrees.

NAtoms= 47 NActive= 47

Polarizable Continuum Model (PCM)

=================================

Model : PCM.

Atomic radii : UFF (Universal Force Field).

Solvent : 1,2-EthaneDiol, Eps= 40.245000 Eps(inf)= 2.050051

SCF Done: E(RB3LYP) = -1071.23697814 A.U. after 1 cycles

Convg = 0.2469D-08 -V/T = 2.0048

	1	2	3
	A	A	A
Frequencies --	-130.0509	15.1192	20.4364

Zero-point correction= 0.393226 (a.u.)

Thermal correction to Energy= 0.426066

Thermal correction to Enthalpy= 0.427011

Thermal correction to Gibbs Free Energy= 0.325251

Sum of electronic and zero-point Energies= -1070.843752

Sum of electronic and thermal Energies= -1070.810912

Sum of electronic and thermal Enthalpies= -1070.809967

Sum of electronic and thermal Free Energies= -1070.911727

	E (Thermal)	CV	S
	KCal/Mol	Cal/Mol-Kelvin	Cal/Mol-Kelvin
Total	267.361	109.990	214.171

Figure 5-3) propane product with DEG

Stoichiometry C7H29N2O9(1-)

Standard orientation:

Center Number	Atomic Number	Atomic Type	Coordinates (Angstroms) X	Y	Z
1	7	0	-3.844194	4.141089	1.437003
2	7	0	-3.063364	3.376633	1.368392
3	1	0	2.000538	1.478347	-0.184011
4	8	0	1.323372	1.516779	-0.889818
5	1	0	0.485239	1.338662	-0.408577
6	1	0	1.977409	0.322445	-2.005980

7	8	0	2.510290	-0.317068	-2.534469
8	6	0	2.929246	-1.362083	-1.654336
9	6	0	1.739304	-2.068353	-1.011122
10	1	0	3.572656	-0.966437	-0.859314
11	1	0	3.520319	-2.062613	-2.247294
12	8	0	2.243665	-2.914153	0.015559
13	1	0	1.058659	-1.314249	-0.600389
14	1	0	1.185359	-2.657405	-1.755488
15	6	0	1.244290	-3.614646	0.757090
16	6	0	0.441149	-2.753254	1.728749
17	1	0	0.551130	-4.120643	0.068844
18	1	0	1.785564	-4.379577	1.319369
19	8	0	1.249156	-2.200017	2.755376
20	1	0	-0.091098	-1.959419	1.193388
21	1	0	-0.324363	-3.408313	2.169317
22	1	0	4.201850	3.882754	-0.300524
23	8	0	4.569158	2.992582	-0.276933
24	1	0	4.022182	2.497727	0.369853
25	1	0	4.628998	2.067236	-1.949308
26	8	0	4.695254	1.588624	-2.796027
27	1	0	3.977233	0.930631	-2.775126
28	1	0	1.200886	-1.202877	2.711721
29	8	0	1.111622	0.453020	2.599775
30	1	0	1.005315	0.845064	3.472734
31	1	0	2.295740	0.953346	1.910869
32	8	0	3.108891	1.249495	1.307081

33	1	0	3.706462	0.493528	1.298721
34	1	0	-0.104772	0.768564	1.598341
35	8	0	-0.760482	0.923719	0.841046
36	1	0	-1.340055	1.639416	1.122956
37	6	0	-6.863062	-0.954020	-1.016590
38	6	0	-7.074081	-0.525453	-2.471849
39	6	0	-7.879528	-0.329870	-0.055675
40	1	0	-6.920689	-2.046179	-0.947291
41	1	0	-5.849745	-0.681570	-0.700808
42	1	0	-6.336191	-0.984676	-3.135778
43	1	0	-8.068481	-0.814959	-2.826999
44	1	0	-6.988255	0.560807	-2.578310
45	1	0	-7.705366	-0.650583	0.975362
46	1	0	-7.823037	0.763242	-0.078988
47	1	0	-8.901881	-0.614089	-0.325372

%chk=wkdeg1.pro.high.chk

Standard basis: 6-311+G(d,p) (6D, 7F)

588 basis functions

79 alpha electrons 79 beta electrons

nuclear repulsion energy 1412.2971995113 Hartrees.

NAtoms= 47 NActive= 47

Force inversion solution in PCM.

Polarizable Continuum Model (PCM)

=================================

Model : PCM (using non-symmetric T matrix).

Atomic radii : UFF (Universal Force Field).

Polarization charges : Total charges.

Solvent : 1,2-EthaneDiol, Eps= 40.245000 Eps(inf)= 2.050051

SCF Done: E(RB3LYP) = -1071.35797737 A.U. after 1 cycles

Zero-point correction= 0.391773 (a.u.)

Thermal correction to Energy= 0.426483

Thermal correction to Enthalpy= 0.427427

Thermal correction to Gibbs Free Energy= 0.308506

Sum of electronic and zero-point Energies= -1070.966204

Sum of electronic and thermal Energies= -1070.931494

Sum of electronic and thermal Enthalpies= -1070.930550

Sum of electronic and thermal Free Energies= -1071.049471

	E (Thermal)	CV	S
	KCal/Mol	Cal/Mol-Kelvin	Cal/Mol-Kelvin
Total	267.622	110.000	250.291

[4] Geometries and energies in Figure 6

I(Ph) Precursor

Stoichiometry C8H27N2O9(1-)

Standard orientation:

Center Number	Atomic Number	Atomic Type	Coordinates (Angstroms) X	Y	Z
1	6	0	-2.260603	-0.508876	-0.635889
2	8	0	-1.404152	0.373048	-0.570875
3	6	0	-3.680480	-0.218812	-0.291057
4	6	0	-1.876843	-1.900061	-1.073274
5	6	0	-4.615152	-1.248453	-0.102053
6	6	0	-5.933087	-0.950028	0.232540
7	6	0	-4.098010	1.114905	-0.154815
8	1	0	-0.837750	-1.908604	-1.396511
9	1	0	-1.992586	-2.599485	-0.239893
10	1	0	-2.520076	-2.249648	-1.883778
11	7	0	1.700971	-2.076392	2.180513
12	7	0	2.598740	-3.199246	2.007427
13	1	0	2.270598	-1.230377	2.155507
14	1	0	1.054030	-2.015257	1.395050
15	1	0	2.796568	-3.348051	1.014718
16	1	0	2.121779	-4.024320	2.355594
17	8	0	3.286787	-0.829444	-1.919992
18	1	0	3.187095	-2.340681	-1.404688
19	1	0	3.551616	-0.831496	-2.846123
20	8	0	3.099001	-3.286149	-1.045306
21	1	0	3.921578	-3.726299	-1.282133

22	8	0	3.441230	0.520368	2.006993
23	1	0	2.837393	1.195065	1.643951
24	1	0	4.065309	0.376554	1.262913
25	8	0	5.002119	0.418910	-0.332683
26	1	0	4.382015	-0.042501	-0.979036
27	1	0	4.900734	1.372463	-0.495270
28	1	0	2.059008	0.129035	-1.743958
29	8	0	1.336351	0.837805	-1.602819
30	1	0	0.507801	0.399851	-1.359390
31	1	0	1.725356	1.833112	-0.299971
32	8	0	1.923920	2.422119	0.478594
33	1	0	1.044013	2.727278	0.767922
34	1	0	-1.147486	1.964654	0.450501
35	8	0	-0.858234	2.771168	0.916586
36	1	0	-1.250076	2.715802	1.795227
37	1	0	4.865744	3.846272	0.162268
38	8	0	4.374936	3.286667	-0.448931
39	1	0	3.487136	3.170464	-0.048259
40	6	0	-6.335829	0.378446	0.366564
41	6	0	-5.416590	1.411083	0.167824
42	1	0	-4.318679	-2.284341	-0.204015
43	1	0	-6.644586	-1.752533	0.386237
44	1	0	-3.386196	1.913818	-0.317766
45	1	0	-7.363672	0.609713	0.621371
46	1	0	-5.730744	2.443813	0.262377

%chk=wk02hbph.high.chk

Standard basis: 6-311+G(d,p) (6D, 7F)

599 basis functions

81 alpha electrons 81 beta electrons

nuclear repulsion energy 1552.4511357870 Hartrees.

NAtoms= 46 NActive= 46

Polarizable Continuum Model (PCM)

==================================

Model : PCM.

Atomic radii : UFF (Universal Force Field).

Solvent : 1,2-EthaneDiol, Eps= 40.245000 Eps(inf)= 2.050051

SCF Done: E(RB3LYP) = -1108.26958834 A.U. after 1 cycles

Zero-point correction= 0.377107 (a.u.)

Thermal correction to Energy= 0.411161

Thermal correction to Enthalpy= 0.412105

Thermal correction to Gibbs Free Energy= 0.302976

Sum of electronic and zero-point Energies= -1107.892481

Sum of electronic and thermal Energies= -1107.858428

Sum of electronic and thermal Enthalpies= -1107.857483

Sum of electronic and thermal Free Energies= -1107.966612

	E (Thermal)	CV	S
	KCal/Mol	Cal/Mol-Kelvin	Cal/Mol-Kelvin
Total	258.007	113.444	229.681

II(Ph) Mulliken CT complex

Stoichiometry   C8H27N2O9(1-)

Standard orientation:

Center Number	Atomic Number	Atomic Type	Coordinates (Angstroms) X	Y	Z
1	6	0	-1.207036	0.169552	1.011770
2	8	0	-0.416657	-0.901606	1.207225
3	6	0	-2.510342	-0.173650	0.262273
4	6	0	-1.429244	0.960343	2.311673
5	6	0	-3.774584	0.118519	0.781866
6	6	0	-4.936465	-0.265143	0.106913
7	6	0	-2.441350	-0.860464	-0.957734
8	1	0	-0.457908	1.216089	2.738128
9	1	0	-2.012639	1.875865	2.186820
10	1	0	-1.951789	0.322172	3.025433
11	7	0	-0.378018	1.152664	0.063018
12	7	0	-1.074550	2.327555	-0.426801
13	1	0	0.519058	1.421578	0.550725
14	1	0	-0.078830	0.604124	-0.762544
15	1	0	-1.348095	2.862177	0.394551
16	1	0	-0.354121	2.887670	-0.895921

17	8	0	2.172451	1.918694	0.797869
18	1	0	1.850529	2.999728	-0.448231
19	1	0	2.525544	2.512367	1.469602
20	8	0	1.543835	3.592676	-1.195901
21	1	0	2.057590	3.314226	-1.961579
22	8	0	1.003216	-0.298021	-2.096979
23	1	0	0.992353	-1.188944	-1.699549
24	1	0	1.893913	0.067371	-1.888703
25	8	0	3.423938	0.824072	-1.312904
26	1	0	3.106028	1.221289	-0.458133
27	1	0	4.056718	0.118395	-1.078091
28	1	0	2.214669	0.424182	1.753856
29	8	0	2.048838	-0.417582	2.250029
30	1	0	1.114559	-0.626637	1.989754
31	1	0	2.858457	-1.772277	1.469171
32	8	0	3.160431	-2.534073	0.915193
33	1	0	2.367686	-2.728144	0.377473
34	1	0	0.147976	-1.966987	0.168956
35	8	0	0.675243	-2.574952	-0.448065
36	1	0	0.128246	-3.348380	-0.621337
37	1	0	5.420288	-1.936944	-1.271868
38	8	0	5.100786	-1.349840	-0.578567
39	1	0	4.432499	-1.869673	-0.066787
40	6	0	-4.852933	-0.940679	-1.107335
41	6	0	-3.595862	-1.234844	-1.639416
42	1	0	-3.873329	0.646654	1.721346

43	1	0	-5.905183	-0.030844	0.534337
44	1	0	-1.477626	-1.108326	-1.384968
45	1	0	-5.753414	-1.235490	-1.634167
46	1	0	-3.514940	-1.759508	-2.585053

%chk=wk02qxph.high.chk

Standard basis: 6-311+G(d,p) (6D, 7F)

599 basis functions

81 alpha electrons 81 beta electrons

nuclear repulsion energy 1755.6479857914 Hartrees.

NAtoms= 46 NActive= 46

Polarizable Continuum Model (PCM)

=================================

Model : PCM.

Atomic radii : UFF (Universal Force Field).

Polarization charges : Total charges.

Solvent: 1,2-EthaneDiol, Eps= 40.245000 Eps(inf)= 2.050051

SCF Done: E(RB3LYP) = -1108.26444217 A.U. after 1 cycles

Convg = 0.3565D-08 -V/T = 2.0048

Zero-point correction= 0.384224 (a.u.)

Thermal correction to Energy= 0.413861

Thermal correction to Enthalpy= 0.414805

Thermal correction to Gibbs Free Energy= 0.324865

Sum of electronic and zero-point Energies= -1107.880218

Sum of electronic and thermal Energies= -1107.850581

Sum of electronic and thermal Enthalpies= -1107.849637

Sum of electronic and thermal Free Energies= -1107.939577

	E (Thermal)	CV	S
	KCal/Mol	Cal/Mol-Kelvin	Cal/Mol-Kelvin
Total	259.702	106.986	189.295

III(Ph) TS1

Stoichiometry C8H27N2O9(1-)

Standard orientation:

Center Number	Atomic Number	Atomic Type	Coordinates (Angstroms) X	Y	Z
1	6	0	-1.010953	0.507163	0.628588
2	8	0	-0.219791	-0.466892	1.303011
3	6	0	-2.360033	-0.093788	0.202751
4	6	0	-1.129205	1.689045	1.588249
5	6	0	-3.504771	0.076537	0.991050
6	6	0	-4.713533	-0.522849	0.639324
7	6	0	-2.461009	-0.881727	-0.953006
8	1	0	-0.135557	2.098258	1.774577

9	1	0	-1.762416	2.477072	1.180174
10	1	0	-1.548706	1.362341	2.539324
11	7	0	-0.212283	0.921387	-0.587600
12	7	0	-0.785493	1.934478	-1.435053
13	1	0	0.735242	1.326021	-0.255972
14	1	0	0.143503	0.024138	-1.272358
15	1	0	-1.767471	1.722025	-1.586464
16	1	0	-0.697914	2.838522	-0.970556
17	8	0	2.137266	2.034649	-0.011807
18	1	0	1.411815	3.469448	-0.057604
19	1	0	2.479295	1.808051	0.860355
20	8	0	0.827970	4.294267	-0.124696
21	1	0	1.258019	4.865400	-0.768988
22	8	0	0.695229	-0.959565	-2.022042
23	1	0	0.735500	-1.955069	-1.035848
24	1	0	1.593852	-0.614630	-2.161130
25	8	0	3.309391	0.499582	-1.899129
26	1	0	2.928860	1.080478	-1.180734
27	1	0	3.911782	-0.117961	-1.447825
28	1	0	1.284163	-0.213177	2.308485
29	8	0	2.162876	-0.256132	2.739417
30	1	0	1.987343	-0.319472	3.684698
31	1	0	2.878634	-1.735168	1.832969
32	8	0	3.103891	-2.449261	1.205045
33	1	0	2.270147	-2.597443	0.705111
34	1	0	-0.073519	-1.282161	0.760250

35	8	0	0.692122	-2.571462	-0.153097
36	1	0	0.223575	-3.385281	-0.366242
37	1	0	5.376438	-2.123315	-1.004928
38	8	0	4.972939	-1.407391	-0.502896
39	1	0	4.329679	-1.838817	0.109419
40	6	0	-4.802064	-1.302829	-0.512104
41	6	0	-3.670729	-1.478499	-1.306601
42	1	0	-3.467324	0.678850	1.888832
43	1	0	-5.585654	-0.375245	1.266111
44	1	0	-1.598199	-1.037672	-1.588473
45	1	0	-5.742367	-1.766105	-0.788218
46	1	0	-3.725651	-2.080824	-2.206345

Standard basis: 6-311+G(d,p) (6D, 7F)

599 basis functions

81 alpha electrons 81 beta electrons

nuclear repulsion energy 1754.4739234728 Hartrees.

NAtoms= 46 NActive= 46

Polarizable Continuum Model (PCM)

=================================

Model : PCM.

Atomic radii : UFF (Universal Force Field).

Solvent : 1,2-EthaneDiol, Eps= 40.245000 Eps(inf)= 2.050051

SCF Done: E(RB3LYP) = -1108.24518308 A.U. after 1 cycles

	1	2	3
	A	A	A
Frequencies --	-608.4014	19.2120	32.8316

Zero-point correction= 0.376632 (a.u.)

Thermal correction to Energy= 0.406391

Thermal correction to Enthalpy= 0.407336

Thermal correction to Gibbs Free Energy= 0.316005

Sum of electronic and zero-point Energies= -1107.868551

Sum of electronic and thermal Energies= -1107.838792

Sum of electronic and thermal Enthalpies= -1107.837848

Sum of electronic and thermal Free Energies= -1107.929178

	E (Thermal)	CV	S
	KCal/Mol	Cal/Mol-Kelvin	Cal/Mol-Kelvin
Total	255.014	106.501	192.221

IV(Ph) (Ph)(Me)C(OH)-NH-NH2

Stoichiometry C8H27N2O9(1-)

Standard orientation:

Center Number	Atomic Number	Atomic Type	Coordinates (Angstroms) X	Y	Z

1	6	0	-1.181172	0.678727	0.552360
2	8	0	-0.456969	-0.334566	1.274366
3	6	0	-2.579541	0.151252	0.193304
4	6	0	-1.194980	1.889601	1.489469
5	6	0	-3.723211	0.534337	0.902005
6	6	0	-4.973378	-0.004163	0.593051
7	6	0	-2.724815	-0.786434	-0.838966
8	1	0	-0.166777	2.213348	1.658628
9	1	0	-1.757929	2.723097	1.066143
10	1	0	-1.639417	1.636169	2.452725
11	7	0	-0.399988	0.935720	-0.685060
12	7	0	-0.929245	1.932974	-1.569642
13	1	0	0.553463	1.249181	-0.415542
14	1	0	0.357329	-0.407673	-1.774873
15	1	0	-1.743235	1.548120	-2.038939
16	1	0	-1.237965	2.762067	-1.062518
17	8	0	2.353580	1.695402	-0.110223
18	1	0	2.751478	3.207106	-0.194083
19	1	0	2.473036	1.358309	0.785896
20	8	0	2.978177	4.201515	-0.272479
21	1	0	3.899979	4.231064	-0.546190
22	8	0	0.889083	-1.121635	-2.202764
23	1	0	0.665349	-2.239215	-0.954138
24	1	0	1.817866	-0.780453	-2.139397
25	8	0	3.341579	0.000374	-1.806597
26	1	0	2.997090	0.674867	-1.115776

27	1	0	3.924181	-0.608125	-1.318443
28	1	0	1.060024	-0.094778	2.223343
29	8	0	1.950695	-0.121641	2.634979
30	1	0	1.809162	-0.011050	3.581652
31	1	0	2.614269	-1.725315	1.986423
32	8	0	2.833765	-2.536431	1.484830
33	1	0	2.034498	-2.702693	0.947206
34	1	0	-0.335894	-1.137554	0.727878
35	8	0	0.474937	-2.669418	-0.075047
36	1	0	-0.017703	-3.478086	-0.254297
37	1	0	5.255909	-2.653897	-0.609525
38	8	0	4.885741	-1.857938	-0.213515
39	1	0	4.181974	-2.167278	0.403948
40	6	0	-5.102982	-0.936764	-0.433603
41	6	0	-3.971205	-1.325588	-1.150294
42	1	0	-3.654281	1.258579	1.703183
43	1	0	-5.844939	0.309709	1.156486
44	1	0	-1.858146	-1.096195	-1.409286
45	1	0	-6.073946	-1.353955	-0.675126
46	1	0	-4.057741	-2.047315	-1.954822

%chk=wk02qqph.for.high.chk

Standard basis: 6-311+G(d,p) (6D, 7F)

599 basis functions

81 alpha electrons 81 beta electrons

nuclear repulsion energy 1715.2608935938 Hartrees.

NAtoms=   46 NActive=   46

Polarizable Continuum Model (PCM)

=================================

Model   : PCM.

Atomic radii   : UFF (Universal Force Field).

Solvent : 1,2-EthaneDiol, Eps= 40.245000 Eps(inf)=   2.050051

SCF Done: E(RB3LYP) = -1108.26021604   A.U. after   2 cycles

Zero-point correction=   0.381389 (a.u.)

Thermal correction to Energy=   0.412339

Thermal correction to Enthalpy=   0.413283

Thermal correction to Gibbs Free Energy=   0.317909

Sum of electronic and zero-point Energies=   -1107.878827

Sum of electronic and thermal Energies=   -1107.847877

Sum of electronic and thermal Enthalpies=   -1107.846933

Sum of electronic and thermal Free Energies=   -1107.942307

	E (Thermal)	CV	S
	KCal/Mol	Cal/Mol-Kelvin	Cal/Mol-Kelvin
Total	258.747	109.133	200.731

V(Ph)   TS2

%chk=wk02qrph.high.chk

Stoichiometry   C8H27N2O9(1-)

Standard orientation:

Center Number	Atomic Number	Atomic Type	Coordinates (Angstroms) X	Y	Z
1	6	0	-1.298320	0.831436	0.481829
2	8	0	-0.538246	-0.097679	1.272130
3	6	0	-2.682861	0.239961	0.175625
4	6	0	-1.338612	2.116314	1.314087
5	6	0	-3.836620	0.651410	0.850561
6	6	0	-5.070853	0.053267	0.590100
7	6	0	-2.799249	-0.786294	-0.772559
8	1	0	-0.317990	2.474790	1.461214
9	1	0	-1.914627	2.901654	0.821412
10	1	0	-1.781272	1.936801	2.294563
11	7	0	-0.542699	1.001072	-0.786349
12	7	0	-1.083112	1.927271	-1.733967
13	1	0	0.402355	1.316329	-0.559724
14	1	0	0.396312	-0.593767	-1.871819
15	1	0	-1.858153	1.478226	-2.211302
16	1	0	-1.449207	2.765359	-1.283062
17	8	0	2.579548	1.579015	-0.240480
18	1	0	3.543546	3.028099	-0.357044

19	1	0	2.526064	1.219502	0.660225
20	8	0	4.043941	3.876940	-0.418356
21	1	0	4.970350	3.618937	-0.456397
22	8	0	0.973650	-1.356486	-2.045515
23	1	0	0.630767	-2.136915	-0.766443
24	1	0	2.038319	-0.935392	-1.940518
25	8	0	3.203264	-0.397693	-1.800675
26	1	0	2.863414	0.799247	-0.839835
27	1	0	3.778515	-1.020186	-1.333588
28	1	0	1.015932	0.261504	2.073515
29	8	0	1.963324	0.322722	2.331567
30	1	0	1.986677	0.737532	3.201170
31	1	0	2.555952	-1.465974	2.061476
32	8	0	2.681471	-2.373563	1.726539
33	1	0	1.873987	-2.535429	1.193489
34	1	0	-0.404481	-0.948022	0.795611
35	8	0	0.373555	-2.495581	0.164025
36	1	0	-0.152633	-3.291969	0.034282
37	1	0	5.026945	-3.226610	-0.282394
38	8	0	4.822918	-2.322413	-0.021908
39	1	0	4.081000	-2.395442	0.620614
40	6	0	-5.172535	-0.967672	-0.352171
41	6	0	-4.029460	-1.385129	-1.034387
42	1	0	-3.789329	1.444145	1.585725
43	1	0	-5.951854	0.389959	1.124910
44	1	0	-1.919970	-1.115493	-1.312034

45 1 0 -6.131197 -1.431056 -0.555780

46 1 0 -4.095095 -2.176069 -1.773173

Standard basis: 6-311+G(d,p) (6D, 7F)

There are 599 symmetry adapted basis functions of A symmetry.

599 basis functions

81 alpha electrons 81 beta electrons

nuclear repulsion energy 1705.2489126941 Hartrees.

NAtoms= 46 NActive= 46

Polarizable Continuum Model (PCM)

=================================

Model : PCM.

Atomic radii : UFF (Universal Force Field).

Solvent: 1,2-EthaneDiol, Eps= 40.245000 Eps(inf)= 2.050051

SCF Done: E(RB3LYP) = -1108.25697459 A.U. after 2 cycles

Convg = 0.5504D-09 -V/T = 2.0048

	1	2	3
	A	A	A
Frequencies --	-508.2039	16.6401	23.2120

Zero-point correction= 0.377199 (a.u.)

Thermal correction to Energy= 0.407812

Thermal correction to Enthalpy= 0.408756

Thermal correction to Gibbs Free Energy= 0.313248

Sum of electronic and zero-point Energies= -1107.879775

Sum of electronic and thermal Energies= -1107.849163

Sum of electronic and thermal Enthalpies= -1107.848219

Sum of electronic and thermal Free Energies= -1107.943726

	E (Thermal)	CV	S
	KCal/Mol	Cal/Mol-Kelvin	Cal/Mol-Kelvin
Total	255.906	107.642	201.013

VI(Ph) (Ph)(Me)C(OH)-NH-NH2 with the different position of OH-

Stoichiometry C8H27N2O9(1-)

Standard orientation:

Center Number	Atomic Number	Atomic Type	Coordinates (Angstroms) X	Y	Z
1	6	0	-1.035801	0.658345	0.861275
2	8	0	-0.180729	-0.403545	1.256499
3	6	0	-2.316629	0.106926	0.217113
4	6	0	-1.348324	1.454746	2.137954
5	8	0	3.074042	-2.861180	-1.183002
6	1	0	3.387509	-3.100167	-0.299789
7	6	0	-3.199372	-0.652761	0.998693
8	6	0	-4.364942	-1.184360	0.453496

9	6	0	-4.676278	-0.963748	-0.889216
10	1	0	-0.427909	1.879438	2.547520
11	1	0	-2.062488	2.259622	1.954525
12	1	0	-1.783144	0.796890	2.889882
13	7	0	-0.233711	1.461479	-0.117268
14	7	0	-0.611620	2.822303	-0.359738
15	1	0	0.710475	1.485095	0.260122
16	1	0	0.358488	0.443566	-1.740246
17	1	0	-1.324811	2.845870	-1.081004
18	1	0	-1.001886	3.264681	0.470537
19	8	0	3.403111	1.526212	0.290945
20	1	0	2.575309	3.113249	-0.244150
21	1	0	3.109957	0.987017	1.048461
22	8	0	2.115267	3.917527	-0.553560
23	1	0	1.172444	3.663177	-0.595219
24	8	0	0.733627	-0.264491	-2.300988
25	1	0	0.628082	-1.060646	-1.711286
26	1	0	2.532391	-0.095572	-2.169498
27	8	0	3.481381	-0.169853	-1.919100
28	1	0	3.489753	0.901226	-0.463790
29	1	0	3.548330	-1.109645	-1.651113
30	1	0	1.343860	-0.146199	2.087865
31	8	0	2.288287	-0.119523	2.374006
32	1	0	2.287430	0.185213	3.288540
33	1	0	2.402409	-2.118698	2.065951
34	8	0	2.193250	-2.985559	1.682956

35	1	0	1.525079	-2.773806	0.987431
36	1	0	-0.002581	-1.093399	0.518975
37	8	0	0.554285	-2.217884	-0.438017
38	1	0	-0.127992	-2.871163	-0.629065
39	1	0	2.108965	-2.677388	-1.012326
40	6	0	-2.632052	0.307020	-1.129403
41	6	0	-3.803870	-0.218886	-1.677968
42	1	0	-2.970550	-0.845394	2.039832
43	1	0	-5.029611	-1.771950	1.076871
44	1	0	-5.585441	-1.373457	-1.314384
45	1	0	-1.956346	0.856849	-1.769986
46	1	0	-4.026959	-0.048215	-2.725190

%chk=wk02qph.rev.high.chk

Standard basis: 6-311+G(d,p) (6D, 7F)

599 basis functions

81 alpha electrons 81 beta electrons

nuclear repulsion energy 1750.2584100175 Hartrees.

NAtoms= 46 NActive= 46

Polarizable Continuum Model (PCM)

=================================

Model : PCM.

Atomic radii : UFF (Universal Force Field).

Solvent : 1,2-EthaneDiol, Eps= 40.245000 Eps(inf)= 2.050051

SCF Done: E(RB3LYP) = -1108.27330899   A.U. after   2 cycles

Zero-point correction=   0.384226 (a.u.)

Thermal correction to Energy=   0.413995

Thermal correction to Enthalpy=   0.414939

Thermal correction to Gibbs Free Energy=   0.323906

Sum of electronic and zero-point Energies=   -1107.889083

Sum of electronic and thermal Energies=   -1107.859314

Sum of electronic and thermal Enthalpies=   -1107.858370

Sum of electronic and thermal Free Energies=   -1107.949403

	E (Thermal)	CV	S
	KCal/Mol	Cal/Mol-Kelvin	Cal/Mol-Kelvin
Total	259.786	107.704	191.595

VII(Ph) TS3

Stoichiometry   C8H27N2O9(1-)

Standard orientation:

Center Number	Atomic Number	Atomic Type	Coordinates (Angstroms) X	Y	Z
1	6	0	-1.076616	-1.249465	-0.865631
2	8	0	-0.110365	-1.647756	0.790531

3	6	0	-1.171679	-2.669247	-1.359383
4	6	0	-2.315466	-0.666916	-0.241440
5	1	0	-0.189046	-3.076380	-1.582531
6	1	0	-1.677529	-3.288395	-0.622552
7	1	0	-1.762485	-2.672492	-2.281049
8	6	0	-2.932640	0.447788	-0.823098
9	6	0	-4.119380	0.957548	-0.296002
10	6	0	-4.709672	0.355228	0.811915
11	7	0	-0.331471	-0.383484	-1.558518
12	7	0	0.721331	-0.839392	-2.389664
13	1	0	-0.240103	0.568393	-1.180652
14	1	0	4.018348	-0.683455	-1.972763
15	1	0	1.632511	-0.633587	-1.951049
16	1	0	0.666794	-0.311518	-3.256120
17	8	0	0.342777	2.083898	-0.147377
18	1	0	-0.670063	3.529331	-0.108742
19	1	0	0.374152	1.543866	0.696502
20	8	0	-1.230805	4.338652	-0.101632
21	1	0	-2.135831	4.014148	-0.147924
22	8	0	3.452628	-0.394433	-1.248498
23	1	0	3.005368	-1.561755	-0.336521
24	1	0	3.126925	1.214001	-1.236459
25	8	0	2.960084	2.189919	-1.052977
26	1	0	1.276015	2.192262	-0.440932
27	1	0	3.560956	2.359443	-0.314658
28	1	0	0.080549	-0.674256	1.346149

29	8	0	0.404634	0.441721	1.942335
30	1	0	-0.208281	0.627095	2.661466
31	1	0	1.979350	-0.075749	2.458972
32	8	0	2.867339	-0.494533	2.605647
33	1	0	2.862575	-1.239143	1.975499
34	1	0	0.778529	-1.968146	0.531409
35	8	0	2.632351	-2.231004	0.348709
36	1	0	3.032948	-3.085900	0.160639
37	1	0	4.441177	0.274429	0.105068
38	8	0	4.804720	0.732761	0.900599
39	1	0	4.206485	0.432375	1.612082
40	6	0	-2.924103	-1.274552	0.865469
41	6	0	-4.108238	-0.764916	1.388648
42	1	0	-2.499088	0.910034	-1.700616
43	1	0	-4.583137	1.820343	-0.760155
44	1	0	-5.632440	0.750171	1.221223
45	1	0	-2.452710	-2.130222	1.328849
46	1	0	-4.561126	-1.241366	2.250574

%chk=wk02qyyph.higha.chk

Standard basis: 6-311+G(d,p) (6D, 7F)

599 basis functions

81 alpha electrons 81 beta electrons

nuclear repulsion energy 1739.9172538678 Hartrees.

NAtoms= 46 NActive= 46

Polarizable Continuum Model (PCM)

=====================================

Model : PCM.

Atomic radii : UFF (Universal Force Field).

Solvent: 1,2-EthaneDiol, Eps= 40.245000 Eps(inf)= 2.050051

SCF Done: E(RB3LYP) = -1108.24347037 A.U. after 1 cycles

	1	2	3
	A	A	A
Frequencies --	-545.6756	18.5490	22.3175

Zero-point correction= 0.376349 (a.u.)

Thermal correction to Energy= 0.406631

Thermal correction to Enthalpy= 0.407575

Thermal correction to Gibbs Free Energy= 0.314126

Sum of electronic and zero-point Energies= -1107.867122

Sum of electronic and thermal Energies= -1107.836839

Sum of electronic and thermal Enthalpies= -1107.835895

Sum of electronic and thermal Free Energies= -1107.929345

	E (Thermal)	CV	S
	KCal/Mol	Cal/Mol-Kelvin	Cal/Mol-Kelvin
Total	255.165	107.553	196.681

VIII(Ph) acetophenone hydrazone

%chk=wk03kxyph.rev.high.chk

Stoichiometry C8H27N2O9(1-)

Standard orientation:

Center Number	Atomic Number	Atomic Type	Coordinates (Angstroms) X	Y	Z
1	6	0	2.336694	-0.115675	-0.333390
2	6	0	1.295197	-0.602040	0.645367
3	6	0	3.780077	-0.167735	0.025306
4	1	0	1.702250	-0.708586	1.649098
5	1	0	0.464722	0.108078	0.687950
6	1	0	0.875692	-1.567029	0.342923
7	6	0	4.264469	-1.117102	0.938061
8	6	0	5.621950	-1.180887	1.252465
9	6	0	6.520990	-0.289442	0.670491
10	7	0	2.048635	0.361093	-1.493020
11	7	0	0.708057	0.471629	-1.848371
12	1	0	0.090411	-0.293442	-1.551554
13	8	0	-1.549005	-1.524741	-1.410337
14	1	0	0.659107	0.565871	-2.856093
15	1	0	-1.892149	-1.675223	-2.298183
16	1	0	-1.109573	-3.067045	-0.648430
17	8	0	-0.873748	-3.916961	-0.211011

18	1	0	-0.038618	-4.181084	-0.610175
19	1	0	-0.222465	2.079761	-1.221853
20	8	0	-0.765729	2.838858	-0.920728
21	1	0	-1.688399	2.607088	-1.182678
22	1	0	-0.016797	3.100276	2.158513
23	8	0	-0.891595	2.928762	1.794182
24	1	0	-0.782952	2.919772	0.809908
25	1	0	-1.794210	1.460610	2.320201
26	8	0	-2.360678	0.685843	2.526161
27	1	0	-1.782673	0.060007	2.975105
28	1	0	-2.286067	-0.986549	-0.908222
29	8	0	-3.354657	-0.187134	-0.213280
30	1	0	-3.065201	0.025044	0.687862
31	1	0	-3.614986	1.914099	-2.371111
32	8	0	-3.343732	2.073338	-1.461254
33	1	0	-3.384754	1.166761	-0.983244
34	1	0	-4.695893	-0.943240	-0.190021
35	8	0	-5.601594	-1.428125	-0.220733
36	1	0	-5.413297	-2.360087	-0.071007
37	1	0	-6.955318	-0.772243	0.684849
38	8	0	-7.721976	-0.419515	1.193171
39	1	0	-8.260287	0.044907	0.544486
40	6	0	4.698005	0.725832	-0.552713
41	6	0	6.050339	0.666585	-0.233043
42	1	0	3.585783	-1.825923	1.396926
43	1	0	5.974250	-1.929573	1.953113

44	1	0	7.574919	-0.334156	0.920091
45	1	0	4.335542	1.470963	-1.249379
46	1	0	6.739505	1.372058	-0.683971

Standard basis: 6-311+G(d,p) (6D, 7F)

599 basis functions

81 alpha electrons 81 beta electrons

nuclear repulsion energy 1532.5150451063 Hartrees.

NAtoms= 46 NActive= 46

Polarizable Continuum Model (PCM)

==================================

Model : PCM (using non-symmetric T matrix).

Atomic radii : UFF (Universal Force Field).

Polarization charges : Total charges.

Solvent : 1,2-EthaneDiol, Eps= 40.245000 Eps(inf)= 2.050051

SCF Done: E(RB3LYP) = -1108.27831742 A.U. after 1 cycles

Zero-point correction= 0.372602 (a.u.)

Thermal correction to Energy= 0.407624

Thermal correction to Enthalpy= 0.408568

Thermal correction to Gibbs Free Energy= 0.295817

Sum of electronic and zero-point Energies= -1107.905715

Sum of electronic and thermal Energies= -1107.870694

Sum of electronic and thermal Enthalpies= -1107.869750

Sum of electronic and thermal Free Energies= -1107.982501

	E (Thermal)	CV	S
	KCal/Mol	Cal/Mol-Kelvin	Cal/Mol-Kelvin
Total	255.788	115.296	237.304

IX(Ph) TS4

Stoichiometry C8H27N2O9(1-)

Standard orientation:

Center Number	Atomic Number	Atomic Type	Coordinates (Angstroms) X	Y	Z
1	6	0	1.265878	-0.467840	-0.125200
2	6	0	0.376181	-1.503222	0.518682
3	6	0	2.716173	-0.458617	0.179567
4	1	0	0.813978	-1.885947	1.441107
5	1	0	-0.594866	-1.060418	0.750441
6	1	0	0.185280	-2.353370	-0.144300
7	6	0	3.379326	-1.646318	0.538485
8	6	0	4.746919	-1.656956	0.810032
9	6	0	5.489577	-0.479153	0.740520
10	7	0	0.809875	0.425852	-0.947374
11	7	0	-0.530474	0.454632	-1.241265

12	1	0	-1.266448	-0.634575	-1.598290
13	8	0	-1.984760	-1.479265	-1.935616
14	1	0	-0.632431	1.161240	-1.968141
15	1	0	-2.204435	-1.293137	-2.855973
16	1	0	-1.494659	-3.150383	-1.873082
17	8	0	-1.272386	-4.113541	-1.837927
18	1	0	-0.420291	-4.190300	-2.278874
19	1	0	-1.419260	1.343336	0.014450
20	8	0	-1.910390	1.940367	0.654906
21	1	0	-2.834905	1.947067	0.325113
22	1	0	-1.341367	0.556014	3.481419
23	8	0	-2.189148	0.614394	3.027970
24	1	0	-2.021143	1.124739	2.197559
25	1	0	-3.038915	-0.896255	2.609646
26	8	0	-3.581645	-1.653303	2.292999
27	1	0	-3.068185	-2.444425	2.488774
28	1	0	-3.426410	-1.268924	-0.949394
29	8	0	-4.229570	-1.097388	-0.397923
30	1	0	-3.994369	-1.367473	0.512457
31	1	0	-4.863925	1.961193	-1.049167
32	8	0	-4.543188	1.597373	-0.217129
33	1	0	-4.498112	0.615833	-0.343793
34	1	0	1.378825	2.002835	-1.614817
35	8	0	1.358822	2.940292	-1.944617
36	1	0	2.267847	3.174791	-2.157863
37	8	0	-0.350397	4.265627	-0.044390

38	1	0	0.264871	3.875450	-0.691851
39	1	0	-0.920521	3.523739	0.231844
40	6	0	3.479844	0.722446	0.127249
41	6	0	4.844166	0.712010	0.399821
42	1	0	2.827358	-2.577356	0.589780
43	1	0	5.231859	-2.590446	1.074109
44	1	0	6.551858	-0.485825	0.956350
45	1	0	2.990889	1.658812	-0.107536
46	1	0	5.404584	1.639844	0.359377

%chk=wk03kxph.high.chk

Standard basis: 6-311+G(d,p) (6D, 7F)

599 basis functions

81 alpha electrons 81 beta electrons

nuclear repulsion energy 1621.1790549560 Hartrees.

NAtoms= 46 NActive= 46

Polarizable Continuum Model (PCM)

=================================

Model : PCM.

Atomic radii : UFF (Universal Force Field).

Solvent: 1,2-EthaneDiol, Eps= 40.245000 Eps(inf)= 2.050051

SCF Done: E(RB3LYP) = -1108.27030694 A.U. after 2 cycles

1 2 3

	A	A	A
Frequencies --	-728.4069	15.0843	20.3828

Zero-point correction= 0.372097 (a.u.)

Thermal correction to Energy= 0.405057

Thermal correction to Enthalpy= 0.406002

Thermal correction to Gibbs Free Energy= 0.303440

Sum of electronic and zero-point Energies= -1107.898210

Sum of electronic and thermal Energies= -1107.865250

Sum of electronic and thermal Enthalpies= -1107.864305

Sum of electronic and thermal Free Energies= -1107.966867

	E (Thermal)	CV	S
	KCal/Mol	Cal/Mol-Kelvin	Cal/Mol-Kelvin
Total	254.177	112.895	215.858

X(Ph) anion intermediate

Stoichiometry C8H27N2O9(1-)

Standard orientation:

Center Number	Atomic Number	Atomic Type	Coordinates (Angstroms) X	Y	Z
1	6	0	1.304518	-0.512990	-0.175408
2	6	0	0.384643	-1.553244	0.415524

3	6	0	2.747000	-0.534478	0.146833
4	1	0	0.811157	-2.007243	1.310349
5	1	0	-0.570416	-1.094242	0.680916
6	1	0	0.159944	-2.356014	-0.296131
7	6	0	3.386372	-1.736235	0.509204
8	6	0	4.750028	-1.774982	0.796814
9	6	0	5.518789	-0.613122	0.739457
10	7	0	0.866827	0.405559	-0.988006
11	7	0	-0.459551	0.453922	-1.298225
12	1	0	-1.459928	-0.716385	-1.675885
13	8	0	-2.205234	-1.396069	-1.954343
14	1	0	-0.540424	1.191758	-1.997936
15	1	0	-2.416380	-1.189603	-2.872356
16	1	0	-1.886810	-3.167699	-1.834141
17	8	0	-1.787396	-4.142368	-1.771251
18	1	0	-0.944441	-4.334832	-2.194417
19	1	0	-1.309733	1.330305	-0.021083
20	8	0	-1.755996	1.935543	0.647924
21	1	0	-2.684338	2.013661	0.340267
22	1	0	-1.208955	0.482025	3.439720
23	8	0	-2.063297	0.614747	3.015200
24	1	0	-1.880884	1.114174	2.180558
25	1	0	-3.059031	-0.806853	2.633143
26	8	0	-3.681683	-1.509318	2.337009
27	1	0	-3.232895	-2.345455	2.502260
28	1	0	-3.640168	-1.098595	-0.878957

29	8	0	-4.403453	-0.878285	-0.301524
30	1	0	-4.150331	-1.170597	0.598494
31	1	0	-4.752665	2.231568	-0.957706
32	8	0	-4.443048	1.843402	-0.132439
33	1	0	-4.508791	0.863174	-0.247604
34	1	0	1.485221	1.950162	-1.633819
35	8	0	1.512811	2.892436	-1.958669
36	1	0	2.434681	3.086208	-2.156791
37	8	0	-0.122179	4.219859	-0.000411
38	1	0	0.471561	3.832243	-0.669489
39	1	0	-0.709917	3.486027	0.261158
40	6	0	3.538826	0.630768	0.109310
41	6	0	4.899646	0.591306	0.395299
42	1	0	2.817743	-2.657717	0.549042
43	1	0	5.212328	-2.719548	1.062872
44	1	0	6.578236	-0.641951	0.967073
45	1	0	3.072729	1.578985	-0.124209
46	1	0	5.478442	1.508371	0.364127

%chk=wk03kxph.for.high.chk

Standard basis: 6-311+G(d,p) (6D, 7F)

599 basis functions

81 alpha electrons 81 beta electrons

nuclear repulsion energy 1613.1198358480 Hartrees.

NAtoms= 46 NActive= 46

Polarizable Continuum Model (PCM)

=================================

Model : PCM.

Atomic radii : UFF (Universal Force Field).

Solvent: 1,2-EthaneDiol, Eps= 40.245000 Eps(inf)= 2.050051

SCF Done: E(RB3LYP) = -1108.27093351 A.U. after 1 cycles

Zero-point correction= 0.375079 (a,u.)

Thermal correction to Energy= 0.408698

Thermal correction to Enthalpy= 0.409643

Thermal correction to Gibbs Free Energy= 0.304954

Sum of electronic and zero-point Energies= -1107.895855

Sum of electronic and thermal Energies= -1107.862235

Sum of electronic and thermal Enthalpies= -1107.861291

Sum of electronic and thermal Free Energies= -1107.965979

	E (Thermal)	CV	S
	KCal/Mol	Cal/Mol-Kelvin	Cal/Mol-Kelvin
Total	256.462	114.368	220.335

XI(Ph) TS5

Stoichiometry C8H27N2O9(1-)

Standard orientation:

Center Number	Atomic Number	Atomic Type	Coordinates (Angstroms) X	Y	Z
1	6	0	-0.502817	-1.355157	-0.668882
2	6	0	-0.243423	-1.955940	-2.051379
3	6	0	-1.918033	-0.904063	-0.431454
4	1	0	-0.720294	-1.353296	-2.825546
5	1	0	0.819622	-2.015259	-2.280761
6	1	0	-0.652132	-2.973089	-2.112715
7	6	0	-2.468672	0.118881	-1.224111
8	6	0	-3.775041	0.562933	-1.029316
9	6	0	-4.570219	-0.003503	-0.031128
10	7	0	0.053956	-1.996357	0.431128
11	7	0	1.039939	-2.779351	0.249009
12	1	0	2.611988	-2.331710	-0.783394
13	8	0	3.445479	-1.906932	-1.085307
14	1	0	1.359013	-3.073968	1.177951
15	1	0	3.181601	-1.325751	-1.826425
16	1	0	3.623449	-0.640221	0.154069
17	8	0	3.503555	0.114832	0.778361
18	1	0	4.253313	0.703146	0.634801
19	1	0	1.691155	-0.425671	3.331720
20	8	0	2.659549	-0.421216	3.467613

21	1	0	3.019708	-0.253077	2.579055
22	1	0	0.246308	-0.201588	-0.631671
23	8	0	0.940825	0.871031	-0.644850
24	1	0	1.715622	0.689968	-0.082283
25	1	0	1.885910	0.469548	-2.150278
26	8	0	2.548590	0.147271	-2.805663
27	1	0	2.068674	0.030662	-3.632649
28	1	0	-0.128128	1.562754	0.573726
29	8	0	-0.700905	1.896501	1.312671
30	1	0	-1.604543	1.767384	0.997969
31	1	0	1.008895	2.510292	-1.296360
32	8	0	0.986181	3.451975	-1.594348
33	1	0	1.854138	3.625355	-1.974074
34	1	0	-0.158159	-1.074103	2.129142
35	8	0	-0.113041	-0.460520	2.895635
36	1	0	-0.356271	0.401629	2.509562
37	1	0	0.428946	4.399337	-0.045769
38	8	0	0.056597	4.655029	0.818757
39	1	0	-0.265008	3.805682	1.167637
40	6	0	-2.736182	-1.470323	0.559357
41	6	0	-4.042385	-1.022973	0.760348
42	1	0	-1.862714	0.580256	-1.996645
43	1	0	-4.169960	1.357525	-1.653070
44	1	0	-5.585978	0.342325	0.123347
45	1	0	-2.345077	-2.270970	1.175086
46	1	0	-4.651355	-1.480104	1.533058

%chk=wk03kyph.higha.chk

Standard basis: 6-311+G(d,p) (6D, 7F)

599 basis functions

81 alpha electrons 81 beta electrons

nuclear repulsion energy 1715.7245643786 Hartrees.

NAtoms= 46 NActive= 46

Polarizable Continuum Model (PCM)

==================================

Model : PCM.

Atomic radii : UFF (Universal Force Field).

Solvent : 1,2-EthaneDiol, Eps= 40.245000 Eps(inf)= 2.050051

SCF Done: E(RB3LYP) = -1108.23755267 A.U. after 2 cycles

	1	2	3
	A	A	A
Frequencies --	-1314.6546	17.9406	27.5998

Zero-point correction= 0.373826 (a.u.)

Thermal correction to Energy= 0.405986

Thermal correction to Enthalpy= 0.406930

Thermal correction to Gibbs Free Energy= 0.309003

Sum of electronic and zero-point Energies= -1107.863727

Sum of electronic and thermal Energies= -1107.831567

Sum of electronic and thermal Enthalpies= -1107.830623

Sum of electronic and thermal Free Energies= -1107.928549

	E (Thermal)	CV	S
	KCal/Mol	Cal/Mol-Kelvin	Cal/Mol-Kelvin
Total	254.760	112.426	206.104

XII(Ph) trans-di-imine

Stoichiometry C8H27N2O9(1-)

Standard orientation:

Center Number	Atomic Number	Atomic Type	Coordinates (Angstroms) X	Y	Z
1	6	0	-2.202569	-2.051724	0.140746
2	6	0	-3.094072	-2.948826	-0.716058
3	6	0	-2.624997	-0.587640	0.127440
4	1	0	-3.102531	-2.636287	-1.760801
5	1	0	-2.750753	-3.984862	-0.675246
6	1	0	-4.116035	-2.905833	-0.332917
7	6	0	-2.599075	0.148819	-1.064790
8	6	0	-2.993693	1.485954	-1.083086
9	6	0	-3.417520	2.108133	0.093178

10	7	0	-0.744275	-2.083674	-0.199598
11	7	0	-0.446044	-2.614562	-1.274527
12	1	0	0.595921	-2.523090	-1.406589
13	1	0	-2.234792	-2.392829	1.180070
14	1	0	1.309003	-0.262905	1.651879
15	8	0	1.077438	-1.184455	1.878059
16	1	0	0.442411	-1.465572	1.181183
17	8	0	2.306385	-2.157460	-1.732892
18	1	0	3.090583	-2.140006	-0.360147
19	1	0	2.701409	-2.718085	-2.408825
20	8	0	3.518190	-2.003190	0.556046
21	1	0	2.751935	-1.887250	1.147576
22	8	0	1.912936	1.526060	1.434853
23	1	0	1.412889	1.774835	0.630432
24	1	0	2.819514	1.326945	1.122216
25	8	0	0.648274	1.886770	-1.060499
26	1	0	-0.211543	1.446894	-1.083348
27	1	0	1.298165	1.279198	-1.520092
28	8	0	2.585611	0.383745	-2.104976
29	1	0	3.298847	0.582813	-1.476537
30	1	0	2.464779	-0.631265	-2.024585
31	8	0	4.274001	0.547642	0.296575
32	1	0	5.199069	0.724816	0.497401
33	1	0	4.121158	-0.429892	0.435215
34	1	0	0.609670	3.608008	-1.674212
35	8	0	0.589386	4.527834	-2.007604

36	1	0	0.764710	5.075939	-1.235819
37	1	0	1.252192	1.360860	3.243777
38	8	0	0.870802	0.961601	4.045005
39	1	0	0.694679	0.053962	3.761208
40	6	0	-3.046971	0.045884	1.298927
41	6	0	-3.443959	1.384230	1.283302
42	1	0	-2.273015	-0.324401	-1.984802
43	1	0	-2.971962	2.041211	-2.014145
44	1	0	-3.723437	3.147871	0.079452
45	1	0	-3.067431	-0.509859	2.230358
46	1	0	-3.770250	1.859069	2.201687

%chk=wk03kfph.for.high.chk

Standard basis: 6-311+G(d,p) (6D, 7F)

599 basis functions

81 alpha electrons 81 beta electrons

nuclear repulsion energy 1667.8856144937 Hartrees.

NAtoms= 46 NActive= 46

Polarizable Continuum Model (PCM)

=================================

Model : PCM (using non-symmetric T matrix).

Atomic radii : UFF (Universal Force Field).

Polarization charges : Total charges.

Solvent : 1,2-EthaneDiol, Eps= 40.245000 Eps(inf)= 2.050051

SCF Done: E(RB3LYP) = -1108.26069137 A.U. after 1 cycles

Zero-point correction= 0.376935(a.u.)

Thermal correction to Energy= 0.409684

Thermal correction to Enthalpy= 0.410628

Thermal correction to Gibbs Free Energy= 0.308072

Sum of electronic and zero-point Energies= -1107.883756

Sum of electronic and thermal Energies= -1107.851008

Sum of electronic and thermal Enthalpies= -1107.850063

Sum of electronic and thermal Free Energies= -1107.952619

	E (Thermal)	CV	S
	KCal/Mol	Cal/Mol-Kelvin	Cal/Mol-Kelvin
Total	257.080	112.021	215.846

XIII(Ph) TS6

Stoichiometry C8H27N2O9(1-)

Standard orientation:

Center Number	Atomic Number	Atomic Type	Coordinates (Angstroms) X	Y	Z
1	6	0	1.022494	2.490136	-0.099964
2	6	0	0.953661	3.442494	-1.280050

3	6	0	2.293779	1.704189	0.037948
4	1	0	0.972330	2.908798	-2.231658
5	1	0	0.022263	4.013451	-1.250355
6	1	0	1.790534	4.145539	-1.255025
7	6	0	2.969032	1.181610	-1.077606
8	6	0	4.105054	0.389064	-0.922827
9	6	0	4.590955	0.090171	0.353590
10	7	0	-0.176635	1.426023	-0.098012
11	7	0	-0.813415	1.297849	-1.126662
12	1	0	-1.817392	0.324488	-0.975946
13	1	0	0.844241	3.031086	0.833367
14	1	0	3.078127	-1.799919	0.340926
15	8	0	2.568810	-2.625991	0.336260
16	1	0	3.178143	-3.301283	0.659850
17	8	0	-2.654055	-0.439253	-0.874876
18	1	0	-4.166720	-0.028380	-1.434385
19	1	0	-2.261429	-1.327337	-0.971627
20	8	0	-5.104310	0.267518	-1.612202
21	1	0	-5.062471	0.845917	-2.380864
22	1	0	-0.580196	-3.118481	-1.356014
23	8	0	-1.366848	-3.023328	-0.787524
24	1	0	-0.985327	-2.899599	0.107810
25	8	0	-0.798427	0.036407	2.291834
26	1	0	-0.539775	0.547892	1.478952
27	1	0	-1.775808	0.022917	2.263583
28	8	0	-3.527546	-0.254435	1.704881

29	1	0	-4.328243	0.295467	1.639405
30	1	0	-3.233663	-0.361965	0.764727
31	8	0	-5.849226	1.230418	0.860358
32	1	0	-6.731775	0.918386	1.086816
33	1	0	-5.690663	0.934394	-0.065040
34	1	0	1.743483	-3.025733	-1.304905
35	8	0	1.182827	-3.279219	-2.063212
36	1	0	1.438159	-2.690349	-2.781765
37	1	0	0.869640	-2.519786	1.334692
38	8	0	-0.046103	-2.551094	1.658105
39	1	0	-0.272536	-1.628426	1.922423
40	6	0	2.791566	1.391120	1.313191
41	6	0	3.925330	0.596485	1.473002
42	1	0	2.609239	1.396266	-2.076814
43	1	0	4.614261	0.003434	-1.799318
44	1	0	5.483356	-0.514206	0.472653
45	1	0	2.286950	1.784341	2.189535
46	1	0	4.293422	0.376198	2.468999

%chk=wk03kfphc.high.chk

Standard basis: 6-311+G(d,p) (6D, 7F)

599 basis functions

81 alpha electrons 81 beta electrons

nuclear repulsion energy 1632.4715697274 Hartrees.

NAtoms= 46 NActive= 46

Polarizable Continuum Model (PCM)

=====================================

Model : PCM.

Solvent: 1,2-EthaneDiol, Eps= 40.245000 Eps(inf)= 2.050051

SCF Done: E(RB3LYP) = -1108.24228190 A.U. after 1 cycles

	1	2	3
	A	A	A
Frequencies --	-603.1623	17.0690	21.0736

Zero-point correction= 0.372240 (a.u.)

Thermal correction to Energy= 0.405008

Thermal correction to Enthalpy= 0.405952

Thermal correction to Gibbs Free Energy= 0.304845

Sum of electronic and zero-point Energies= -1107.870042

Sum of electronic and thermal Energies= -1107.837274

Sum of electronic and thermal Enthalpies= -1107.836330

Sum of electronic and thermal Free Energies= -1107.937437

	E (Thermal)	CV	S
	KCal/Mol	Cal/Mol-Kelvin	Cal/Mol-Kelvin
Total	254.146	112.847	212.798

XIV(Ph) carbanion

%chk=wk03kfphc.highfor.chk

Stoichiometry C8H27N2O9(1-)

Standard orientation:

Center Number	Atomic Number	Atomic Type	Coordinates (Angstroms) X	Y	Z
1	6	0	2.670067	2.563539	0.605051
2	6	0	3.127480	3.418861	-0.547390
3	6	0	2.996184	1.227604	0.749050
4	1	0	2.794315	3.047658	-1.530996
5	1	0	2.742026	4.438566	-0.452814
6	1	0	4.224829	3.503234	-0.624254
7	6	0	3.799627	0.493981	-0.218033
8	6	0	4.126347	-0.837010	-0.045909
9	6	0	3.680185	-1.587629	1.065808
10	7	0	-0.395747	2.539856	-2.047597
11	7	0	0.181510	2.673675	-2.969404
12	1	0	-3.426494	0.810859	-2.086181
13	1	0	2.073587	3.037539	1.380274
14	1	0	2.231492	-2.576810	-0.042337
15	8	0	1.567854	-3.103580	-0.551189
16	1	0	1.970931	-3.972160	-0.672001

17	8	0	-3.480258	0.272628	-1.288277
18	1	0	-5.117416	0.128662	-0.535181
19	1	0	-3.000803	-0.577453	-1.489112
20	8	0	-5.910514	0.079119	0.040951
21	1	0	-6.636489	0.453432	-0.469666
22	1	0	-1.366296	-1.960298	-2.214364
23	8	0	-2.190675	-2.040304	-1.693433
24	1	0	-1.875159	-2.362021	-0.817621
25	8	0	-0.610581	-0.532582	2.105683
26	1	0	0.296140	-0.224854	1.947197
27	1	0	-1.211510	0.174077	1.775132
28	8	0	-2.426984	1.312424	1.158760
29	1	0	-3.250874	1.328209	1.686168
30	1	0	-2.714780	1.018843	0.273149
31	8	0	-4.984444	1.255630	2.360926
32	1	0	-5.139222	0.683583	3.120439
33	1	0	-5.438061	0.825249	1.603364
34	1	0	0.898661	-2.343711	-2.092224
35	8	0	0.383563	-2.009646	-2.855102
36	1	0	0.801990	-1.179376	-3.107782
37	1	0	-0.125124	-3.067284	0.330677
38	8	0	-1.015998	-2.875031	0.676499
39	1	0	-0.895528	-2.064226	1.219554
40	6	0	2.582928	0.433579	1.898061
41	6	0	2.921295	-0.895145	2.042829
42	1	0	4.164688	1.014211	-1.097384

43	1	0	4.745383	-1.322592	-0.797524
44	1	0	4.038193	-2.595230	1.246212
45	1	0	2.033383	0.934606	2.692658
46	1	0	2.601424	-1.423374	2.938273

Standard basis: 6-311+G(d,p) (6D, 7F)

599 basis functions

81 alpha electrons 81 beta electrons

nuclear repulsion energy 1554.6253685742 Hartrees.

NAtoms= 46 NActive= 46

Polarizable Continuum Model (PCM)

==================================

Model : PCM.

Atomic radii : UFF (Universal Force Field).

Solvent : 1,2-EthaneDiol, Eps= 40.245000 Eps(inf)= 2.050051

SCF Done: E(RB3LYP) = -1108.27748911 A.U. after 1 cycles

Convg = 0.1852D-08 -V/T = 2.0048

Zero-point correction= 0.370331 (a.u.)

Thermal correction to Energy= 0.406641

Thermal correction to Enthalpy= 0.407585

Thermal correction to Gibbs Free Energy= 0.294583

Sum of electronic and zero-point Energies= -1107.907158

Sum of electronic and thermal Energies= -1107.870849

Sum of electronic and thermal Enthalpies= -1107.869904

Sum of electronic and thermal Free Energies= -1107.982907

	E (Thermal)	CV	S
	KCal/Mol	Cal/Mol-Kelvin	Cal/Mol-Kelvin
Total	255.171	120.338	237.833

XV(Ph) TS7

Stoichiometry C8H27N2O9(1-)

Standard orientation:

Center Number	Atomic Number	Atomic Type	Coordinates (Angstroms) X	Y	Z
1	6	0	3.347353	-1.945726	-1.009195
2	6	0	4.038314	-3.062900	-0.270213
3	6	0	3.167131	-0.678227	-0.533420
4	1	0	3.695569	-3.156195	0.768041
5	1	0	3.852704	-4.023793	-0.757457
6	1	0	5.130635	-2.939027	-0.222896
7	6	0	3.636685	-0.233115	0.781454
8	6	0	3.393795	1.026919	1.245549
9	6	0	2.577402	1.987430	0.534234
10	7	0	-1.252593	0.718791	-4.070789

11	7	0	-0.677820	0.621627	-4.997512
12	1	0	-3.231727	0.171191	1.917719
13	1	0	2.979383	-2.169063	-2.007580
14	1	0	1.298952	2.004233	1.217827
15	8	0	0.217088	2.017857	1.757026
16	1	0	0.370269	2.373991	2.642278
17	8	0	-3.637549	-0.713333	2.116139
18	1	0	-2.220483	-1.941955	2.121198
19	1	0	-4.231368	-0.590332	2.864731
20	8	0	-1.457134	-2.518723	1.925926
21	1	0	-0.675694	-1.929158	1.994993
22	1	0	-1.541015	1.684612	1.423868
23	8	0	-2.504256	1.574920	1.269034
24	1	0	-2.628391	1.474933	0.302236
25	8	0	-3.224012	1.009513	-1.375659
26	1	0	-2.586350	0.942145	-2.095330
27	1	0	-3.556122	0.095066	-1.219761
28	8	0	-4.191800	-1.453256	-0.586099
29	1	0	-3.532262	-2.169679	-0.696340
30	1	0	-4.182616	-1.270462	0.372391
31	8	0	-2.129896	-3.353038	-0.583512
32	1	0	-2.314384	-4.298324	-0.575867
33	1	0	-1.798446	-3.133050	0.320751
34	1	0	0.393944	0.224775	2.071538
35	8	0	0.630521	-0.717378	2.225540
36	1	0	1.521115	-0.794851	1.852297

37	1	0	-0.355369	3.567445	0.903327
38	8	0	-0.648923	4.399331	0.475664
39	1	0	-1.607666	4.320447	0.429852
40	6	0	2.498201	0.358680	-1.320767
41	6	0	2.258874	1.603451	-0.834125
42	1	0	4.221999	-0.918792	1.385408
43	1	0	3.794138	1.310127	2.216863
44	1	0	2.818081	3.039629	0.699894
45	1	0	2.192299	0.103914	-2.332329
46	1	0	1.766859	2.333019	-1.473107

%chk=wk03kfphc.higha.chk

Standard basis: 6-311+G(d,p) (6D, 7F)

599 basis functions

81 alpha electrons 81 beta electrons

nuclear repulsion energy 1570.8151649738 Hartrees.

NAtoms= 46 NActive= 46

Polarizable Continuum Model (PCM)

=================================

Model : PCM.

Atomic radii : UFF (Universal Force Field).

Solvent: 1,2-EthaneDiol, Eps= 40.245000 Eps(inf)= 2.050051

SCF Done: E(RB3LYP) = -1108.26473219   A.U. after   1 cycles

	1	2	3
	A	A	A
Frequencies --	-1337.1100	4.3014	8.8429

Zero-point correction=   0.366182 (a.u.)

Thermal correction to Energy=   0.401919

Thermal correction to Enthalpy=   0.402863

Thermal correction to Gibbs Free Energy=   0.288308

Sum of electronic and zero-point Energies=   -1107.898550

Sum of electronic and thermal Energies=   -1107.862813

Sum of electronic and thermal Enthalpies=   -1107.861869

Sum of electronic and thermal Free Energies=   -1107.976424

	E (Thermal)	CV	S
	KCal/Mol	Cal/Mol-Kelvin	Cal/Mol-Kelvin
Total	252.208	117.875	241.102

XVI(Ph)   a 3-ethylidene-1,4-cyclohexadiene intermediate

Stoichiometry   C8H27N2O9(1-)

Standard orientation:

Center   Atomic   Atomic   Coordinates (Angstroms)

Number	Number	Type	X	Y	Z
1	6	0	-4.268160	-1.216959	1.263572
2	6	0	-4.899894	-2.424456	0.640875
3	6	0	-3.775064	-0.118067	0.645112
4	1	0	-4.948460	-2.376437	-0.447060
5	1	0	-4.347681	-3.331944	0.911663
6	1	0	-5.920540	-2.563602	1.016327
7	6	0	-3.780593	0.085779	-0.808923
8	6	0	-3.287611	1.195880	-1.382377
9	6	0	-2.684176	2.330466	-0.607325
10	7	0	2.797354	3.397328	1.865943
11	7	0	2.345482	4.301980	2.285514
12	1	0	3.354250	-2.004883	-1.282357
13	1	0	-4.202817	-1.238884	2.349844
14	1	0	-1.658407	2.519647	-0.957790
15	8	0	1.194793	0.378007	-2.788078
16	1	0	1.187715	0.362073	-3.751218
17	8	0	3.240204	-2.857183	-0.766319
18	1	0	1.416535	-2.979074	-0.350695
19	1	0	3.713556	-3.545809	-1.245353
20	8	0	0.503958	-2.886708	-0.015441
21	1	0	0.060815	-2.264538	-0.641315
22	1	0	2.604556	-0.130811	-2.258645
23	8	0	3.491603	-0.482712	-1.906091
24	1	0	3.732182	0.067548	-1.137892

25	8	0	4.296305	0.685424	0.589785
26	1	0	3.812085	1.415164	0.992002
27	1	0	4.078289	-0.111442	1.125107
28	8	0	3.725716	-1.743144	1.796644
29	1	0	2.820109	-1.791497	2.167210
30	1	0	3.669059	-2.263793	0.971897
31	8	0	1.010888	-1.951637	2.486667
32	1	0	0.709122	-2.594885	3.136867
33	1	0	0.717645	-2.296523	1.606688
34	1	0	0.074167	-0.548586	-2.229845
35	8	0	-0.624278	-1.184410	-1.833375
36	1	0	-1.377276	-0.639115	-1.573314
37	1	0	0.864315	1.896657	-2.283921
38	8	0	0.636250	2.823421	-1.959809
39	1	0	1.463341	3.184222	-1.625433
40	6	0	-3.187312	0.975271	1.431029
41	6	0	-2.691300	2.089280	0.874706
42	1	0	-4.204976	-0.687646	-1.438961
43	1	0	-3.321342	1.298722	-2.463193
44	1	0	-3.222989	3.262355	-0.839001
45	1	0	-3.164425	0.851865	2.510177
46	1	0	-2.267533	2.864433	1.506138

%chk=wk03kfphc.rev.high.chk

Standard basis: 6-311+G(d,p) (6D, 7F)

599 basis functions

81 alpha electrons   81 beta electrons

nuclear repulsion energy   1532.2057074485 Hartrees.

NAtoms=   46 NActive=   46

Polarizable Continuum Model (PCM)

=================================

Model   : PCM (using non-symmetric T matrix).

Atomic radii   : UFF (Universal Force Field).

Solvent : 1,2-EthaneDiol, Eps= 40.245000 Eps(inf)=   2.050051

SCF Done: E(RB3LYP) = -1108.29715879   A.U. after 1 cycles

Zero-point correction=   0.369940 (a.u.)

Thermal correction to Energy=   0.405881

Thermal correction to Enthalpy=   0.406826

Thermal correction to Gibbs Free Energy=   0.290380

Sum of electronic and zero-point Energies=   -1107.927219

Sum of electronic and thermal Energies=   -1107.891277

Sum of electronic and thermal Enthalpies=   -1107.890333

Sum of electronic and thermal Free Energies=   -1108.006779

	E (Thermal)	CV	S
	KCal/Mol	Cal/Mol-Kelvin	Cal/Mol-Kelvin
Total	254.694	116.857	245.081

XVII(Ph) TS8

Stoichiometry C8H27N2O9(1-)

Standard orientation:

Center Number	Atomic Number	Atomic Type	Coordinates (Angstroms) X	Y	Z
1	6	0	-0.966579	2.727336	1.298548
2	6	0	-2.021158	2.869923	2.368678
3	6	0	-1.200101	2.356334	-0.005054
4	1	0	-2.758313	2.061386	2.344122
5	1	0	-1.569325	2.864718	3.364593
6	1	0	-2.584115	3.811240	2.283069
7	6	0	-2.502938	1.929260	-0.504003
8	6	0	-2.671508	1.482159	-1.777750
9	6	0	-1.570157	1.317157	-2.709224
10	1	0	-0.332717	0.810171	1.706052
11	1	0	0.021677	3.117157	1.535326
12	1	0	-1.323989	-0.030003	-2.731160
13	8	0	-1.090802	-1.289894	-2.745836
14	1	0	-1.200068	-1.562957	-3.664777
15	8	0	-0.045726	-0.080975	2.033898
16	1	0	0.445496	0.091794	2.847746

17	1	0	-1.736537	-0.726678	2.752647
18	8	0	-2.602334	-1.039897	3.073191
19	1	0	-2.404307	-1.781501	3.655987
20	1	0	1.638121	-0.215114	0.785764
21	8	0	2.389408	-0.361377	0.189843
22	1	0	2.027489	-0.946056	-0.515637
23	1	0	-0.292898	-1.976915	1.219423
24	8	0	-0.348332	-2.841214	0.783741
25	1	0	-1.180827	-2.819511	0.267658
26	1	0	-3.786512	-1.453521	1.649971
27	8	0	-4.376546	-1.674220	0.907188
28	1	0	-3.778311	-1.981420	0.202319
29	1	0	3.903927	-0.909648	0.981871
30	8	0	4.723854	-1.205173	1.432551
31	1	0	5.436801	-0.719778	1.004330
32	1	0	0.431543	-1.814637	-2.042234
33	8	0	1.193598	-2.167210	-1.516782
34	1	0	0.759717	-2.580891	-0.745556
35	1	0	-2.025944	-2.152491	-1.725217
36	8	0	-2.507046	-2.675091	-1.008673
37	1	0	-2.769218	-3.509611	-1.413050
38	6	0	-0.136608	2.378008	-1.004456
39	6	0	-0.323114	1.928052	-2.270877
40	1	0	-3.357521	1.976527	0.161595
41	1	0	-3.666927	1.187898	-2.101741
42	1	0	-1.819088	1.448759	-3.765196

43	1	0	0.830553	2.771418	-0.703758
44	1	0	0.503484	1.976874	-2.975429
45	7	0	7.429735	0.323428	0.075879
46	7	0	8.320578	0.792834	-0.354002

%chk=wk07a.chk

Standard basis: 6-311+G(d,p) (6D, 7F)

599 basis functions

81 alpha electrons 81 beta electrons

nuclear repulsion energy 1568.3001356520 Hartrees.

NAtoms= 46 NActive= 46

Polarizable Continuum Model (PCM)

=================================

Model : PCM.

Atomic radii : UFF (Universal Force Field).

Solvent : 1,2-EthaneDiol, Eps= 40.245000 Eps(inf)= 2.050051

SCF Done: E(RB3LYP) = -1108.26004919 A.U. after 1 cycles

Convg = 0.3568D-08 -V/T = 2.0048

	1	2	3
	A	A	A
Frequencies --	-1379.2308	4.0956	9.6766

Zero-point correction= 0.365988 (a.u.)

Thermal correction to Energy= 0.401619

Thermal correction to Enthalpy= 0.402563

Thermal correction to Gibbs Free Energy= 0.289729

Sum of electronic and zero-point Energies= -1107.894061

Sum of electronic and thermal Energies= -1107.858431

Sum of electronic and thermal Enthalpies= -1107.857486

Sum of electronic and thermal Free Energies= -1107.970321

	E (Thermal)	CV	S
	KCal/Mol	Cal/Mol-Kelvin	Cal/Mol-Kelvin
Total	252.019	118.139	237.479

XVIII(Ph) ethylbenzene product

Stoichiometry C8H27N2O9(1-)

Standard orientation:

Center Number	Atomic Number	Atomic Type	Coordinates (Angstroms) X	Y	Z
1	6	0	-1.469402	2.318957	0.577855
2	6	0	-1.683539	3.406856	1.645154
3	6	0	-2.735118	1.553625	0.261030
4	1	0	-2.035272	2.970672	2.584072
5	1	0	-0.749443	3.937833	1.847180

6	1	0	-2.425512	4.139088	1.315189
7	6	0	-3.081074	0.403718	0.985816
8	6	0	-4.271441	-0.279897	0.725735
9	6	0	-5.137672	0.174840	-0.269343
10	1	0	-0.700873	1.621760	0.923974
11	1	0	-1.087347	2.782365	-0.336566
12	1	0	-2.244109	-1.139705	-0.735199
13	8	0	-1.825558	-1.745310	-1.366994
14	1	0	-2.506528	-1.935979	-2.024371
15	8	0	3.573334	-2.073646	0.153399
16	1	0	4.511546	-2.193610	-0.027452
17	1	0	3.221377	-2.521707	1.593753
18	8	0	2.915694	-2.801566	2.533038
19	1	0	3.508979	-3.510279	2.802094
20	1	0	3.046718	-0.612009	-0.365368
21	8	0	2.648969	0.243963	-0.718878
22	1	0	1.963720	-0.047051	-1.348785
23	1	0	2.638721	-2.901926	-0.880710
24	8	0	2.005220	-3.326548	-1.544867
25	1	0	1.264683	-3.682338	-1.026496
26	1	0	1.170353	-3.276759	2.554783
27	8	0	0.221496	-3.524396	2.581856
28	1	0	-0.031334	-3.667254	1.654661
29	1	0	3.738668	1.485698	-1.268413
30	8	0	4.357231	2.189396	-1.579063
31	1	0	4.169254	2.953588	-1.024498

32	1	0	-0.110023	-1.130676	-2.177743
33	8	0	0.796297	-1.038747	-2.513321
34	1	0	1.230110	-1.889805	-2.265949
35	1	0	-1.049544	-3.238597	-0.588287
36	8	0	-0.509213	-3.953692	-0.195447
37	1	0	-0.988693	-4.772502	-0.365689
38	6	0	-3.614267	1.995072	-0.735878
39	6	0	-4.803449	1.315315	-0.999769
40	1	0	-2.416471	0.044267	1.765450
41	1	0	-4.518794	-1.166536	1.299016
42	1	0	-6.060455	-0.355558	-0.474296
43	1	0	-3.363446	2.878716	-1.314185
44	1	0	-5.466802	1.674204	-1.778940
45	7	0	3.830679	5.007828	0.254837
46	7	0	3.683397	5.916107	0.848219

%chk=wk07a.for.chk

Standard basis: 6-311+G(d,p) (6D, 7F)

599 basis functions

81 alpha electrons 81 beta electrons

nuclear repulsion energy 1475.3246630135 Hartrees.

NAtoms= 46 NActive= 46

Polarizable Continuum Model (PCM)

=====================================

Model : PCM.

Atomic radii : UFF (Universal Force Field).

Solvent: 1,2-EthaneDiol, Eps= 40.245000 Eps(inf)= 2.050051

SCF Done: E(RB3LYP) = -1108.33542604 A.U. after 1 cycles

Convg = 0.4993D-08 -V/T = 2.0048

Zero-point correction= 0.370816 (a.u.)

Thermal correction to Energy= 0.407434

Thermal correction to Enthalpy= 0.408378

Thermal correction to Gibbs Free Energy= 0.287530

Sum of electronic and zero-point Energies= -1107.964610

Sum of electronic and thermal Energies= -1107.927992

Sum of electronic and thermal Enthalpies= -1107.927048

Sum of electronic and thermal Free Energies= -1108.047896

	E (Thermal)	CV	S
	KCal/Mol	Cal/Mol-Kelvin	Cal/Mol-Kelvin
Total	255.669	117.860	254.346

[5] Results of wB97XD/6-311+G** scrf=PCM calculations

IPh precursor in Figure 6 by wB97XD/6-311+G** scrf=PCM

Stoichiometry C8H27N2O9(1-)

Standard orientation:

Center Number	Atomic Number	Atomic Type	Coordinates (Angstroms) X	Y	Z
1	6	0	1.561031	-1.014693	-1.559188
2	8	0	0.914182	-2.021109	-1.298222
3	6	0	2.605717	-0.524472	-0.617041
4	6	0	1.273019	-0.231925	-2.807004
5	6	0	3.172982	0.744839	-0.755075
6	6	0	4.113219	1.194848	0.163195
7	6	0	2.998653	-1.339316	0.448967
8	1	0	0.672862	-0.837950	-3.484075
9	1	0	0.688394	0.651247	-2.519878
10	1	0	2.184697	0.097716	-3.306178
11	7	0	-0.222104	0.392554	1.303010
12	7	0	0.806566	1.211810	1.881063
13	1	0	-1.096998	0.615194	1.773870
14	1	0	-0.367142	0.633132	0.320817
15	1	0	0.859449	2.081121	1.349598
16	1	0	1.686157	0.725379	1.749048
17	8	0	-1.355606	1.502422	-1.458044
18	1	0	-0.455451	2.627812	-0.686117
19	1	0	-1.413381	1.836278	-2.356077
20	8	0	0.112632	3.293000	-0.198085
21	1	0	-0.501587	3.942331	0.147725
22	8	0	-3.153535	0.583411	2.246365
23	1	0	-3.113999	-0.300573	1.845754

24	1	0	-3.349766	1.150324	1.475250
25	8	0	-3.685173	1.767837	-0.208745
26	1	0	-2.827851	1.678965	-0.713049
27	1	0	-4.223413	0.995442	-0.436638
28	1	0	-1.626216	-0.065904	-1.597038
29	8	0	-1.852186	-1.047609	-1.646085
30	1	0	-1.007090	-1.510883	-1.688429
31	1	0	-2.549029	-1.519182	-0.160948
32	8	0	-2.903724	-1.833585	0.704987
33	1	0	-2.115950	-2.231725	1.115355
34	1	0	0.171177	-2.589648	0.549816
35	8	0	-0.230936	-2.373989	1.400163
36	1	0	-0.139159	-1.385654	1.435172
37	1	0	-5.994005	-0.535680	0.372158
38	8	0	-5.269136	-0.670131	-0.241736
39	1	0	-4.636432	-1.234623	0.228984
40	6	0	4.499628	0.377295	1.219661
41	6	0	3.942393	-0.892063	1.361029
42	1	0	2.873060	1.397757	-1.565228
43	1	0	4.541636	2.184039	0.055203
44	1	0	2.559040	-2.323000	0.555168
45	1	0	5.233706	0.728310	1.935741
46	1	0	4.244073	-1.529813	2.183436

Standard basis: 6-311+G(d,p) (6D, 7F)

599 basis functions

81 alpha electrons 81 beta electrons

nuclear repulsion energy 1738.7451028756 Hartrees.

NAtoms= 46 NActive= 46

Force inversion solution in PCM.

Polarizable Continuum Model (PCM)

=====================================

Model : PCM (using non-symmetric T matrix).

Atomic radii : UFF (Universal Force Field).

Polarization charges : Total charges.

Solvent : 1,2-EthaneDiol, Eps= 40.245000 Eps(inf)= 2.050051

Error on total polarization charges = 0.01973

SCF Done: E(RwB97XD) = -1107.90860583 A.U. after 1 cycles

Zero-point correction= 0.386787 (a.u.)

Thermal correction to Energy= 0.418580

Thermal correction to Enthalpy= 0.419524

Thermal correction to Gibbs Free Energy= 0.323724

Sum of electronic and zero-point Energies= -1107.521819

Sum of electronic and thermal Energies= -1107.490026

Sum of electronic and thermal Enthalpies= -1107.489082

Sum of electronic and thermal Free Energies= -1107.584882

E (Thermal) CV S

	KCal/Mol	Cal/Mol-Kelvin	Cal/Mol-Kelvin
Total	262.663	110.489	201.629

XIPh(TS5) wB97XD/6-311+G** scrf=PCM

Stoichiometry C8H27N2O9(1-)

Standard orientation:

Center Number	Atomic Number	Atomic Type	Coordinates (Angstroms) X	Y	Z
1	6	0	-0.079306	-1.311777	-0.862547
2	6	0	0.316521	-1.622224	-2.298870
3	6	0	-1.547073	-1.139297	-0.619934
4	1	0	-0.256578	-1.004427	-2.992122
5	1	0	1.374162	-1.437323	-2.484208
6	1	0	0.118087	-2.675458	-2.535339
7	6	0	-2.230328	-0.104277	-1.273794
8	6	0	-3.577939	0.133350	-1.035105
9	6	0	-4.280440	-0.659339	-0.130304
10	7	0	0.562704	-2.000808	0.151489
11	7	0	1.687310	-2.520868	-0.089139
12	1	0	3.061560	-1.556115	-1.006274
13	8	0	3.749592	-0.895050	-1.222159
14	1	0	2.038087	-2.892390	0.797107

15	1	0	3.332740	-0.287269	-1.860099
16	1	0	3.638771	0.131137	0.202713
17	8	0	3.355835	0.716174	0.940123
18	1	0	3.969950	1.453151	0.951847
19	1	0	1.679585	-0.681354	3.233959
20	8	0	2.629017	-0.527161	3.384426
21	1	0	2.933295	-0.107774	2.564741
22	1	0	0.398626	-0.069374	-0.587462
23	8	0	0.785027	1.116573	-0.373467
24	1	0	1.581462	1.052518	0.178688
25	1	0	1.708501	1.172542	-1.893586
26	8	0	2.368474	1.100724	-2.618538
27	1	0	1.862816	0.921014	-3.413517
28	1	0	-0.469288	1.298517	0.835600
29	8	0	-1.161548	1.340944	1.538931
30	1	0	-1.954021	0.997605	1.113469
31	1	0	0.295234	2.688560	-0.882632
32	8	0	-0.055245	3.571649	-1.141550
33	1	0	0.707683	4.075664	-1.430265
34	1	0	0.059242	-1.470640	1.915300
35	8	0	-0.069118	-1.003944	2.765461
36	1	0	-0.499997	-0.174048	2.501378
37	1	0	-0.938551	4.139849	0.416141
38	8	0	-1.381702	4.193900	1.279297
39	1	0	-1.391097	3.268335	1.565544
40	6	0	-2.271314	-1.938686	0.269691

41	6	0	-3.619605	-1.697449	0.516929
42	1	0	-1.688882	0.542379	-1.957073
43	1	0	-4.077514	0.949619	-1.545387
44	1	0	-5.330259	-0.471165	0.063358
45	1	0	-1.772491	-2.754864	0.779103
46	1	0	-4.156965	-2.329246	1.215987

%chk=wk03kyph.wb97xd.chk

Standard basis: 6-311+G(d,p) (6D, 7F)

599 basis functions

81 alpha electrons 81 beta electrons

nuclear repulsion energy 1749.6462242502 Hartrees.

NAtoms= 46 NActive= 46

Force inversion solution in PCM.

Polarizable Continuum Model (PCM)

=================================

Model : PCM (using non-symmetric T matrix).

Atomic radii : UFF (Universal Force Field).

Solvent: 1,2-EthaneDiol, Eps= 40.245000 Eps(inf)= 2.050051

SCF Done: E(RwB97XD) = -1107.87565365 A.U. after 2 cycles

NFock= 2 Conv=0.56D-09 -V/T= 2.0046

1 2 3

	A	A	A
Frequencies --	-1320.4273	19.5427	28.2478

Zero-point correction= 0.381098 (a.u.)

Thermal correction to Energy= 0.412207

Thermal correction to Enthalpy= 0.413151

Thermal correction to Gibbs Free Energy= 0.318594

Sum of electronic and zero-point Energies= -1107.494556

Sum of electronic and thermal Energies= -1107.463447

Sum of electronic and thermal Enthalpies= -1107.462503

Sum of electronic and thermal Free Energies= -1107.557060

	E (Thermal)	CV	S
	KCal/Mol	Cal/Mol-Kelvin	Cal/Mol-Kelvin
Total	258.664	110.611	199.012
